# Distinct neural networks of task engagement and choice response in moral, risky, and ambiguous decision-making: An ALE meta-analysis

**DOI:** 10.1162/imag_a_00277

**Published:** 2024-08-30

**Authors:** Aiste Ambrase, Veronika I. Müller, Julia A. Camilleri, Hong Yu Wong, Birgit Derntl

**Affiliations:** Department of Psychiatry and Psychotherapy, Tübingen Centre for Mental Health, University of Tübingen, Tübingen, Germany; Institute of Neuroscience and Medicine: Brain and Behaviour (INM-7), Research Centre Jülich, Jülich, Germany; Institute of Systems Neuroscience, Medical Faculty, Heinrich Heine University Düsseldorf, Düsseldorf, Germany; Werner Reichardt Centre for Integrative Neuroscience, University of Tübingen, Tübingen, Germany; Department of Philosophy, University of Tübingen, Tübingen, Germany; German Center for Mental Health (DZPG), Partner Site Tübingen, Tübingen, Germany

**Keywords:** morality, risk, ambiguity, decision-making, choice response, uncertainty, social cognition, frontoparietal network, salience, striatum, fMRI

## Abstract

Moral, risky, and ambiguous decision-making are likely to be characterized by common and distinct cognitive processes and thus show partly overlapping neural correlates. Previously, two different analysis approaches have been used to assess the neural correlates in all three domains: (a) comparing general engagement in an experimental task versus a control task (*task engagement*) or (b) comparing actual opposite choices made during the experimental task (*choice response*). Several coordinate-based activation likelihood estimation meta-analyses were performed to delineate consistent activations across experiments of the two analysis categories and the different decision-making domains. Our results show that*task engagement*and*choice response*capture different aspects of salience network involvement and reward-related striatum processing during decision-making. When assessing domains separately, we discovered that moral cues are processed in a multi-modal social cognition network, while risk and ambiguity require engagement of the salience and the frontoparietal attention networks. This is the first meta-analysis to disentangle the two analysis approaches yielding new insight into common and distinct neural correlates of different kinds of decision-making.

## 1 Introduction

Evaluating choice options and choosing between conflicting alternatives are a common occurrence in our everyday lives. Assigning value to the available choice options allows us to compare them and resolve decisional conflicts (O’Doherty, 2004;Schultz, 2006). To which of the choice options we assign higher value depends on our individual and cultural differences as well as the domain context in which a decision is made (Brosch et al., 2011;Dreher, 2013;Yates & de Oliveira, 2016). Equivalent choice dilemmas might be evaluated and resolved differently if, for example, uncertainty about the outcomes is introduced or impact of the outcomes is extended beyond oneself (Tversky & Kahneman, 1981). Brain lesions and certain mental disorders have domain-selective effects on decision-making, for example, gambling and addiction disorders affect decisions dealing with uncertainty and personal reward (Grant et al., 2011;Manes et al., 2002), while specific frontal lobe lesions and antisocial personality disorder affect decisions in social domains, but not financial or routine decisions (Ciaramelli et al., 2007;Clark & Manes, 2004;Harenski et al., 2010;Thomas et al., 2011). This all suggests that cognitive processes involved in decision-making are based on various distributed neural circuits, whose involvement in neural processing of decision-making is context dependent. On the other hand, more extensive frontal lobe injury or neurodegeneration leads to domain-general dysfunction of decision-making, suggesting that certain cognitive functions and their neural underpinnings underlie decision-making in a domain-general manner (Bechara & Van Der Linden, 2005;Ruiz-Gutiérrez et al., 2020). Therefore, comparing consistent involvement of different brain circuits in different decision-making domains might allow us to better understand how domain-specific and domain-general cognitive functions are related to brain anatomy.

It has been assumed that subjective value computation, a central cognitive function underlying decision-making, is based on a “common currency” of neural signals and shared brain circuitry to allow the decision-maker to evaluate, integrate, and compare choice options from different domains, for example, social and non-social domains, in a similar way (for a detailed review, seeRuff & Fehr, 2014). However, it has been recently shown that the morality domain, a specific subcase of the social domain, might be an exception.Ugazio et al. (2021)demonstrated that subjective value signals for choices in the morality domain are associated with a different pattern of neural activity than financial decisions. They have found that while computationally subjective values in the moral and financial domains are processed similarly, the moral domain engaged a network of activation clusters in the right temporoparietal junction (TPJ), posterior cingulate cortex (PCC), right dorsolateral prefrontal cortex (dlPFC), left inferior parietal lobule (IPL), and the anterior insula (aINS), while the financial domain engaged clusters in the left medial prefrontal cortex (mPFC), right supramarginal gyrus (SMG), and bilateral superior temporal sulcus (STS). This raises the question whether other cognitive processes, such as cognitive control, attention allocation, and conflict resolution, that is, domain-general functions and their neurobiological underpinnings, are involved in the morality domain similarly or differently than in non-social value-based decision-making.

Tasks used in human functional magnetic resonance imaging (fMRI) research of decision conflict in morality, risk, and ambiguity domains usually involve an opposition between two choice alternatives, which differ in their outcome magnitude and uncertainties attached to the alternatives (see, e.g.,Greene et al., 2004;Huettel et al., 2006). Therefore, a decision-making process in these domains might be based on the same principal structure. Animal and human studies have demonstrated that after deconstructing the decision problem, neural signals for subjective stimulus value and potential action costs are computed and associated with the prospective outcomes of every choice alternative (Padoa-Schioppa, 2007;Rangel & Hare, 2010;Rangel et al., 2008). Conflict resolution follows, a process which is based on action value computation (subjective stimulus value minus the action costs) and weighting action values for choice alternatives against each other (Basten et al., 2010;Rangel & Hare, 2010;Rangel et al., 2008). During this cost–benefit analysis, various variables such as prior knowledge, situational framing, uncertainty about outcome probabilities, temporal delay, required effort, physical and social rewards, expected hedonic experience, as well as individual preferences for action type (approach or avoidance) can influence action value computation (Ambrase et al., 2021;Khani & Rainer, 2016). Once a decision is reached, reward anticipation signals are computed, which are compared with reward prediction error upon reward reception to facilitate learning from the choice (Daw et al., 2011;Dayan, 2008). Finally, neural signals for hedonic experience upon reward reception are generated (Berridge & Kringelbach, 2015;Haber & Knutson, 2010;Peciña et al., 2006). Together, these reward- and valuation-related signals constitute a neural valuation system in the brain, comprising the subcortical brain areas such as striatum, ventral tegmental area, substantia nigra, and the cortical brain areas such as prefrontal cortex (PFC), orbitofrontal cortex (OFC), and anterior cingulate cortex (ACC) (Haber, 2016;Haber & Knutson, 2010;Pessiglione & Lebreton, 2015). This neural valuation system interacts with other extended brain circuits, which provide context and stimulus incentive (approach motivation or else, “wanting”) to produce conflict resolution and appropriate action (Basten et al., 2010;Haber & Knutson, 2010). Assumptions about the similarity of the decision-making framework as described above in the morality, risk, and ambiguity domains have already led to the development of multiple computational models for the morality domain based on computational models in financial decision-making (for a review, seeQu et al., 2022).

On the other hand, each domain—morality, risk, and ambiguity—differs in a domain-specific manner. Moral decision-making is an act of choosing a course of action or expressing a personal judgement on the moral permissibility of that action in a situation where the consequences of that action affect oneself or other individuals, based on a personal or socially shared understanding of what is morally permissible or impermissible (Ni et al., 2022). Moral decision-making has been traditionally investigated by using moral dilemmas, that is, situations where both courses of action might be morally permissible or impermissible, depending on an individual’s personal moral inclinations. These scenarios originated from philosophical thought experiments, namely, the Trolley and the Footbridge dilemmas (Foot, 1967;Thomson, 1985). In paradigms employing these moral dilemmas, participants have to choose, in accordance with their moral belief, whether to sacrifice a smaller number of people as collateral damage in order to save a larger number of people (by treating sacrificial harm as morally permissible) or to allow a larger number of people to die without inflicting harm to the bystanders (by upholding a moral norm of not inflicting harm actively). While multiple variations of moral dilemmas involving sacrificial harm have been developed and utilized in studying behavioural and neural correlates of moral decision-making, similar (less lethal) scenarios have been proposed and used as well, such as everyday moral dilemmas, depicting non-lethal bodily harm, stealing, deception, and promise breaking in situations where these actions might also be morally permissible (e.g., lying to save someone’s life) (Greene et al., 2001;May et al., 2022;Singer et al., 2019).

Similarly, moral decision-making in situations where personal (or even selfish) needs conflict with a prosocial course of action poses a similar conundrum—where both courses of action might be morally permissible, depending on individual moral views, for example, keeping the extra money that is left after one’s needs are fully met, as opposed to giving it away to charity. This is known as prosocial decision-making in the neuroimaging literature, and it reflects altruistic, helping behaviours in contrast to selfish ones. Prosocial and altruistic decision-making fall within the morality domain (Singer, 1965) and are measured by various altruistic and charitable decision-making tasks, as well as the Dictator game (Forsythe et al., 1994;Hoffman et al., 1996;Kahneman et al., 1986) or Trust game (Berg et al., 1995) when the resource-sharing actions of the proposer are investigated. In prosocial decision-making, the consequences are rewarding either to the decision-maker or to the recipient of the prosocial action, while being costly to the person who does not receive the reward. The reward–cost distribution in prosocial dilemmas is, however, different from sacrificial harm dilemmas, where sacrificial harm is morally permissible, yet results in dire consequences either way to the recipient of the decision as well as possible moral praise or blame (even self-attributed, seeSjåstad & Baumeister, 2019) and feelings of regret (decision costs) to the decision-maker (Pletti et al., 2016;Tasso et al., 2017). As a result, the two types of decision-making in the morality domain might be similar or differ by valence of outcome to the decision-maker and the target person of the decision—but do not differ by the dilemmatic nature wherein a decision must be made. Therefore, both traditional moral dilemmas that involve a choice of sacrificial harm or harm in general and prosocial decision-making tasks in which selfish and altruistic choices are at odds can be subsumed under the morality domain.

In non-social decision-making, as opposed to moral decision-making, choice outcomes affect solely the decision-maker (at least in experimental setups). Here, one must choose between choice options with possible rewards or losses associated with different levels of outcome probability. Outcome probability indicates how certainly or uncertainly the selected choice option will lead to the desired outcome (Platt & Huettel, 2008). A choice whether to buy a lottery ticket and potentially win a large sum of money as opposed to just saving the cost of the ticket is just one example of many similar choices between options with uncertainty as opposed to options with certainty. Two types of uncertainty can be distinguished: risk and ambiguity. In risky decision-making, one has to choose between two options with different ratios of reward values and outcome probabilities (Fukunaga et al., 2018). Usually, a risky choice has a high reward value but low reward probability, while a safe choice has a low reward value but high reward probability. Gambling tasks such as “Wheel of Fortune” (Ernst et al., 2004), the Cambridge Gambling Task (Rogers et al., 1999), Chicken Game (Osborne & Rubinstein, 1994), and other types of gambles are used to investigate risky decision-making. Importantly, in risky decision-making, outcome probabilities are presented (like in the “Wheel of Fortune,” Cambridge Gambling Task, Chicken Game) or can be easily inferred through visual cues or the frequency of reward reception (Schonberg et al., 2011), like in Iowa Gambling task (Bechara et al., 1994) and Balloon Analog Risk task (Lejuez et al., 2002). In contrast, in ambiguous decision-making, outcome probabilities for the choice options are either not provided or difficult to infer (Camerer & Weber, 1992). This makes ambiguous decision-making an act of choosing between an option whose probabilistic information is lacking while the reward reception is uncertain yet possibly large, as opposed to a safe option when reward probability is known but the reward is small. Alternatively, both of the choice options might have equal probability (like in a coin toss), making the choice ambiguous without any additional information helping to establish option preference, or when reward probability can only be partially inferred in time through exploration and learning (Krain et al., 2006;Taya, 2012).

In order to understand where in the brain the assumed shared cognitive functions also share neural correlates in the domain-general manner—and where they differ—one can perform a meta-analytical investigation of existing neuroimaging studies. Sorting these studies by their different condition comparison strategies would allow us to obtain domain-general and domain-specific neural correlates of decision-making. In neuroimaging experiments, selection of the experimental and control tasks affects the interpretation of results in meaningful ways (Newman et al., 2001). In the decision-making, the chosen condition comparison strategy allows the experimenter to investigate different components of decision-making and their corresponding neural processing. Two broad categories of these strategies can be separated, depending on whether the actual choice is considered or not. In the*task engagement*category, the condition comparison is performed between an experimental task and a control task. Furthermore, depending on the behavioural requirements in the control condition, researchers can investigate neural correlates of a broad set of decision-making processes when a*low-level control condition*is used or neural correlates of a very specific cognitive process when a*high-level control condition*is used. A different approach of analysis—*choice response*—is seen when neural correlates of actual choices are compared during condition comparison. The following sections will describe these analysis categories in more detail.

When investigating*task engagement*, one would traditionally compare the blood-oxygen-level-dependent (BOLD) signal during the experimental decision-making task versus a control task, disregarding what actual choices were made during the task by the participants. While this analysis approach is not exclusive to decision-making tasks, in some domains, such as moral decision-making, researchers typically utilize this approach. As already mentioned, two different analysis categories could be separated in this analysis approach, depending on what control signal is extracted from the signal obtained during the experimental task. In the first case, a low-level control condition is used, for example, a guided motorvisual performance or baseline measurement of resting-state with or without fixation. In the second case, a high-level control condition would require participants to perform a control decision-making task, which differs from the experimental task in some meaningful way, usually in a domain-specific manner.

One of the early fMRI studies in risky decision-making contrasting the “Wheel of Fortune” task with a motorvisual (low-level) control condition postulated that during selection of gamble options, this analysis approach would provide neural correlates of a broad set of cognitive functions, such as “assessing the cue, particularly evaluating the spatial representation of probabilities; making a decision between two competing options, which involves the weighting of possible outcomes; and executing the selected course of action by pressing a button” (Ernst et al., 2004). Similarly, in a recent meta-analysis, the comparison of an experimental task with a resting-state condition was considered as a useful tool to investigate general neural responses to demands of decision-making (Cutler & Campbell-Meiklejohn, 2019). Therefore, contrasting an experimental decision-making task with a low-level control task should track all domain-general cognitive decision-making components in the valuation mechanism, including the assessment and processing of domain-specific situational cues (e.g., information on moral permissibility, probabilistic nature of risk, and complete uncertainty in ambiguity). Depending on the length of BOLD time-series modelled for the analysis, the results might also represent neural correlates (anatomical location and functional activation intensity) for reward anticipation, outcome reception, and outcome evaluation, that is, reward prediction error, related emotional processing of reward reception, and reinforcement learning (Ernst et al., 2004;Labudda et al., 2008;Lawrence et al., 2009).

To investigate shared domain-general processes of decision-making, the comparison of studies using the low-level control condition in different domains allows researchers to investigate whether the neural activation patterns are similarly anatomically distributed across the brain (at least on the gross level, there could be different neuronal populations in the same brain regions specialized for specific domains yet indistinguishable due to the spatial resolution of fMRI, as opponents of a “common neural currency” hypothesis assume, seeRuff & Fehr (2014)for review). We propose that there are reasons to believe that this could be the case.

Evidence exists that neural correlates of cognitive functions such as objective value computation, conflict resolution, and reinforcement in the moral and non-social domains overlap anatomically when functional activation is measured (Crockett et al., 2017;Hutcherson et al., 2015;Lengersdorff et al., 2020). Regarding context and situational cue processing, probabilistic uncertainty about outcome likelihood, even though unintended, also emerges in moral decision-making tasks. It has been demonstrated that participants treat moral sacrificial harm dilemmas similarly to dilemmas containing risk or ambiguity: they implicitly attach probabilistic beliefs about outcome likelihood to choice options in moral dilemmas even when no probabilistic information is provided and the outcomes should be considered as certain (Shou & Song, 2017;Shou et al., 2020).Wiegmann and Waldmann (2014)propose that this is inherent to Trolley-type moral dilemmas where harm is caused from a distance. According to them, the scenarios leave space for imagining other possible events occurring after the decision-makers makes their choice.

Outcome uncertainty in the morality domain should not be confused with social uncertainty.FeldmanHall and Shenhav (2019)argue that in any social context, due to the nature of others’ involvement, social uncertainty arises as decision-makers need to predict beliefs, actions, and reactions of others to their decision-making. For example, when making moral decisions, the decision-makers might be faced with uncertainty on whether their preferred choice is morally permissible (Lockhart, 2000). Furthermore, anticipation of an affective reaction (shame, guilt, regret) to one’s decision indicates that even in hypothetical experiments, participants self-ascribe or imagine others ascribing moral praise or blame to them as moral agents (Pletti et al., 2016;Tasso et al., 2017).Lauharatanahirun et al. (2012)have shown that risk preferences in a social and non-social context, while behaviourally associated with each other, depend on opposite functional activation patterns in the left amygdala, possibly indicating additional decision costs in a social context. Therefore, cognitive functions such as perspective-taking, affect-sharing, or heuristic reference to moral norms might be involved in the morality domain but not in the non-social domains such as decision-making under risk or ambiguity (FeldmanHall & Nassar, 2021;FeldmanHall & Shenhav, 2019). Neural correlates of social uncertainty processing but not probabilistic uncertainty, therefore, would differentiate the morality domain from risk and ambiguity domains, as shown previously (Lauharatanahirun et al., 2012).

In the analysis of the decision-making tasks, if a high-level control task containing neutral decision performance is used in comparison with the experimental task, the BOLD signal representing most of the general cognitive aspects of decision-making behaviour would be removed, leaving only the BOLD signal representing domain-specific stimulus cue processing. For example,van Leijenhorst et al. (2006)visually manipulated reward probabilities in a Cake Guessing Task and contrasted the two conditions when the cakes represented a high risk for reward and a low risk for reward, without regarding whether the participants themselves chose a high-risk or a low—risk option. In the morality domain, researchers interested in the brain networks responsible for moral information processing directly compare moral decision-making or a moral judgement task with a control condition involving similarly structured decision-making or judgement performance in a social or semantic domain (Borg et al., 2006;Heekeren et al., 2001;Moll et al., 2002). This condition comparison strategy results in the remaining BOLD signal that correlates with processing of moral information,^*^including moral norms and social uncertainty. Therefore, the condition comparison strategy with the high-level control condition during decision-making aims to track what domain-specific cues would require attention from the decision-maker and where anatomically these cues induce functional activation in the brain. While the high-level control condition allows the experimenters to distil the stimulus cue processing, this cognitive process would most likely be the only cognitive process shared with the condition comparison when a low-level control condition is used.

In the*choice response*analysis approach, the BOLD signal during the selected choices of interest (e.g., choice to sacrifice someone in a moral dilemma, risky choice in a gamble, or ambiguous choice in a guessing task) would be compared with the BOLD signal of the opposite choice (e.g., choice to abstain from action in a moral dilemma, a safe certain choice option in a gamble or in a guessing task), based on participants’ actual behaviour during the experiment. Investigating BOLD signal changes for specific choices captures neural processing of multiple choice-related cognitive functions: conflict resolution, utility maximization, anticipation of reward or loss, approach activation, and effort (Bujold et al., 2022;Crockett et al., 2014;Glickman et al., 2019;Stillman et al., 2020). Importantly, after the subtraction of the BOLD signals representing opposite choices, differences in anticipation of outcome uncertainty resolution (additionally, anticipation of social uncertainty resolution in the morality domain (Kahane et al., 2012)), outcome anticipation, approach motivation, and subjective value signals associated with the specific choices should remain (Bossaerts, 2010;Fukui et al., 2005;Matthews et al., 2004).

Previous meta-analytical results suggest that different networks underlie the morality, risk, and ambiguity domains. In the morality domain, the (ventro)medial PFC, ACC, lateral OFC, aINS, the amygdala, the TPJ, and the precuneus, that is, brain areas associated with social cognition, emotion, and valuation processes, have been reported most frequently, when moral tasks were compared with control tasks without differentiating the level of control task (for meta-analyses, seeCutler & Campbell-Meiklejohn, 2019;Eres et al., 2018;Fede & Kiehl, 2020;Garrigan et al., 2016;Rhoads et al., 2021). With regard to risky and ambiguous decision-making, previous meta-analyses differed from our analysis approach as they analysed the spatial convergence for experiments that used the condition comparison between experimental and control conditions (*task engagement*), the comparison of opposite actual choices (*choice response*), and parametric modulation of functional activation together. Two recent meta-analyses byPoudel et al. (2020)andWu et al. (2021)demonstrated that aINS and PFC are involved in processing both risk and ambiguity, and that the bilateral caudate nuclei (CN) were more strongly associated with risky decision-making when risky choices in the tasks had higher rewarding outcome and higher outcome uncertainty, while frontal brain areas such as the inferior frontal gyrus, associated with working memory and attention, were more consistently found for ambiguity where tasks involved unknown outcome probability and known possible reward magnitude (Poudel et al., 2020). However, it is yet unclear which brain areas correspond to the condition comparison strategy in*task engagement*or in*choice response*and whether the previous results are not biased by one or another analysis strategy.

Individual studies employing the*choice response*analysis strategy in the morality domain have found that a neural circuit of brain areas associated with social and cognitive processing, such as the dlPFC, mPFC, precuneus, temporal lobe, and subcortical brain areas associated with reward and emotional processing, such as striatum and amygdala underlie choices with higher outcome magnitude, that is, a higher number of saved individuals (Greene et al., 2004;Han et al., 2014;Moll et al., 2006;Sommer et al., 2010;Telzer et al., 2011). In the risk domain, activation of the regions involved in cognitive reasoning, reward, and self-referential processing, such as dlPFC, temporal lobe, occipital lobe, precuneus, ventral striatum (VS), and aINS has been found for risky choices in*choice response*(Ernst et al., 2004;Symmonds et al., 2011;Wright et al., 2013). Concerning the ambiguity domain, activation of the dlPFC, precuneus, aINS, and VS—similar to risky decision-making—as well as parietal lobule, and ACC, both associated with attention and its control, has been found for ambiguous choices when*choice response*was investigated (Furl & Averbeck, 2011;Losecaat Vermeer et al., 2014).

Lesion studies have suggested that with regard to actual choices, damage to the vmPFC, one of the brain areas responsible for valuation processing, leads to higher acceptability of harmful sacrificial actions in moral decision-making (Koenigs et al., 2007;Thomas et al., 2011;Young et al., 2010), to disadvantageous gambling strategies in risky decision-making (Bechara et al., 1994,^1999^;Clark et al., 2008;Sutterer et al., 2015), and to altered aINS response to ambiguous cues in vmPFC lesion patients (Motzkin et al., 2014). Therefore, vmPFC damage seems to predispose lesion patients to prefer the choice options with higher possible outcome magnitude but also higher probabilistic or social uncertainty. It is, however, yet unclear which specific cognitive function in decision-making, for example, subjective value computation or cost–benefit comparison, is affected by these lesions. Taken together with the results from the individual studies discussed above, it seems that the mPFC as well as subcortical brain areas might be rather associated with neural activation of choice-specific preferences rather than the domain specifics. What domain-specific and domain-general neural activation is associated with the different value-based decision-making domains remains to be investigated, as there are no studies available that specifically compared moral decision-making with either risky or ambiguous decision-making.

We argue that when investigating consistent activation across experiments in decision-making, it is important to separate experiments into different categories not only by contextual domain but also by analysis approach. This can allow us to better specify functional parcellation of anatomical brain regions and relate this functional parcellation to various cognitive functions involved in either domain-general cognitive functions of value-based decision-making, or cognitive processing of domain-specific stimulus features, or choice-specific differences in the haemodynamic response corresponding to cognitive processing of choice-specific differences in their probabilistic nature (as in possible outcome variance or social uncertainty) and reward magnitude.

By using activation likelihood estimation (ALE) meta-analysis, we set out to investigate the similarities and differences between moral, risky, and ambiguous decision-making tasks in the brain regions activated consistently. In this meta-analysis, we aim to summarize the neural correlates involved in processing across moral, risky, and ambiguous decision-making and separately. As outlined above, we separated*task engagement*and*choice response*analysis approaches to investigate domain-general (*task engagement*category only including experiments with a low-level control condition), domain-specific (*task engagement*category only including experiments with a high-level control condition), and choice-specific (*choice response*category) neural underpinnings of behaviour in the three domains. To confirm these domain-general and domain-specific functional associations of our meta-analytical results, we have also implemented functional decoding analysis using BrainMap database (Laird et al., 2009,^2013^).

As no previous studies directly compared moral decision-making with either risky or ambiguous decision-making, we could specify hypotheses only for some of our research questions. Generally, we expected to find a broad network of spatial convergence nodes which correspond to the domain-general neural processing of cognitive functions involved in decision-making, when experiments from the three decision-making domains using low-level control conditions were analysed together in the meta-analysis. More specifically, we expected to find convergent activity for the three domains in brain areas that are responsible for value computation, for example, vmPFC, and for uncertainty computation, for example, aINS. Regarding domain-specific functional activation, we expected to find activation of brain areas responsible for social processing, for example, TPJ and precuneus, in the morality domain, but not in the risk or ambiguity domains. As the behavioural literature points to a possible implicit ascription of probabilistic uncertainty to outcome choices in moral dilemmas, the three domains could potentially share consistent activation in the*task engagement category*in brain regions, associated with uncertainty processing, for example, aINS. Based on the literature discussed above, for the*choice response*category, we expect to find activation foci in VS, mPFC, precuneus, and aINS for all three decision-making domains. These areas are similarly involved in processing of outcome magnitude differences and differences in uncertainty between the choices that might or might not bring about higher positive or negative outcome as compared with “safe” choices.

## 2 Methods

### 2.1 Literature search and selection

Preferred Reporting Items for Systematic Review and Meta-Analyses (PRISMA) guidelines and best-practice recommendations for conducting meta-analysis (Müller, Cieslik, et al., 2018) were followed to identify relevant neuroimaging studies and conduct the meta-analysis (seeFig. 1andSupplementary Methodsfor more details). In brief, we performed a literature search in the online databases “PubMed,” “Web of Science,” and “Science Direct” using the following keywords in appropriate combinations: [moral dilemma OR moral decision OR moral choice OR altruism OR altruistic decision OR altruistic choice OR ultimatum game OR donation task OR decision under risk OR risky choice OR risky decision OR ambiguity decision OR ambiguous decision OR decision under ambiguity OR ambiguous choice OR uncertainty decision OR decision under uncertainty OR uncertain decision OR uncertain choice] AND [fMRI OR positron]. Additionally, we traced the relevant references through previous meta-analyses, reviews, and via direct search in open-source database “NeuroSynth” and “Google Scholar.”

**Fig. 1.**
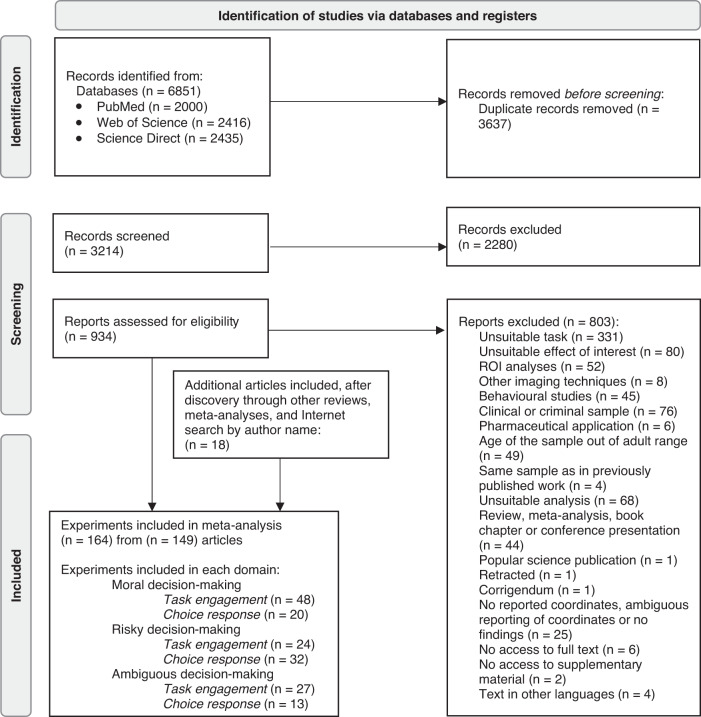
Illustration of the steps during the study eligibility identification process.

During the selection of the studies for the meta-analysis, authors of 40 studies were contacted with a request for additional data. In particular, the authors were contacted and asked for (1) main results from a healthy sample if the study reported contrasts between groups or results for mixed healthy and treatment/patient samples; (2) main results from adult participants only if the study reported contrasts between age groups or results for mixed underaged/senior and adult samples; (3) whole-brain results if the study reported region-of-interest analyses while indicating that whole-brain scans were acquired; (4) condition comparison between experimental task and baseline condition if the study reported that baseline was recorded but analysed differences between actual choices or applied parametric modulation analysis; (5) specification of contrast conditions if the report did not clearly specify them; (6) results in theSupplementary Materialif we did not have access to that. Of them, authors of nine studies provided unpublished results in form of tables of coordinates or activation maps which were in accordance with our inclusion and exclusion criteria. The rest of the studies, for which we did not get the required information, were excluded from the meta-analysis. In case activation maps were provided, coordinates were extracted with JuBrain Anatomy Toolbox Version 3.0 (Eickhoff et al., 2005,^2006^,^2007^). Information regarding which studies were included with unpublished data is disclosed inSupplementary Table 2, along with other information on all included studies.

An ethics statement is not applicable because this study is based on published literature. Unpublished data included in this meta-analysis are derived from studies, which have been approved by their local institutional boards, and related to already published reports.

A systematic approach in selecting articles for the meta-analysis was applied. Research articles written in English and published in peer-reviewed scientific journals were considered and the following a priori inclusion criteria were applied on all experiments: (1) results were obtained by whole-brain analysis using functional magnetic resonance imaging (fMRI) or positron emission tomography, thus results of region-of-interest analyses or other imaging techniques were excluded (Eickhoff et al., 2012), (2) results were obtained by a categorical condition comparison, thus studies using a parametric modulation approach or other analysis types (e.g., correlational, model-based, prediction) were excluded, (3) brain imaging results were reported in the standard system of coordinates, that is, coordinates reported in style of Montreal Neurological Institute (MNI) or Talairach (TAL) format, (4) results only from healthy adult samples (at least 18 years old, maximum mean age up to 55 years) were included, and studies investigating underaged, senior, or mixed samples as well as samples with clinical conditions or criminal samples exclusively, or samples undergoing psychopharmacological intervention were excluded (where reported, results from healthy adult control samples separately from the results from the experimental groups were included in the meta-analysis), (5) results from the same samples were included once, (6) only full and unambiguously reported results were included, any studies that lacked information on exact methodology, analysis steps, or condition comparison strategy, thus making assignment of the experiment into*task engagement*or*choice response*categories challenging, were excluded, (7) the experimental task actively and explicitly measured value-based decisional processes (in a first-person manner) and indicated participant’s choice or appropriateness judgement, and resulted in positively or negatively valenced outcomes (Morriss et al., 2019) for the participants or the protagonists in the experimental task in the three decision-making domains, (8) to avoid biasing meta-analytical results on brain areas processing morality, risk, and ambiguity as contextual cues only, we have excluded brain activation results derived from model-based analyses, parametric modulation, and direct categorical contrast between risky and ambiguous choices, (9) in the*task engagement*category, the contrasts representing comparison of decision-making in morality, risk, and ambiguity domains as compared with control task regardless of actual choices during the decision-making process were included; in the*choice response*category, actual choices, which contained more uncertainty and a possible larger reward/loss (numerically), compared with safe choices, which involved less uncertainty and smaller possible reward/loss, were included.

As experimental tasks in the risk and ambiguity domains represent decision outcomes in both positive (gain) and negative (loss) valence, we sought to reflect this framing effect in the morality domain as well. Thus, in the morality domain, studies employing experimental tasks of (1) dilemmas requiring harming someone or something to save others (from here on “sacrificial harm”), (2) morally relevant emotional or physical harm-related situations (from here on “harm”), (3) altruistic or prosocial choices (from here on “altruism”), and (4) deception were included. In the risk domain, studies investigating (1) gambling and risky investment (from here on “gambling under risk”), (2) naturalistic risk-taking, (3) medical decision-making under risk, and (4) driving or plane landing under risk (from here on “spatial navigation under risk”) were included. Finally, in the ambiguity domain, we included studies examining (1) gambling tasks with partial outcome probability information (from here on “gambling under ambiguity”), (2) binary guessing tasks providing no outcome probability information (from here on “guessing”), (3) decision-making tasks where probabilities can be inferred or learned during multiple runs of the task (from here on “probabilistic learning under ambiguity”), and (4) driving, plane landing, or maze navigation with limited probabilistic outcome information (from here on “spatial navigation under ambiguity”). For more information on domain classification, see theSupplementary Methods: Selection criteria for the tasks.

In addition to classification into morality, risk, or ambiguity domains, all experiments were also classified according to their condition comparison strategy. It is important to note that the content of the experimental task alone did not determine this classification. The combination of both contrast sides, that is, what conditions were compared during the analysis, indicated to which category the experiment was assigned to. The classification was performed as follows: (1)*task engagement (combined)*—studies investigating general aspects of decision-making as determined by the contrasts “task of interest>control task,” “task of interest>(implicit or explicit) baseline,” for example, specifically in the case of this meta-analysis contrasts like “moral decision-making task>non-moral decision-making control task,” “moral decision-making task>baseline,” “risky decision-making task>safe decision-making task,” “risky decision-making task>baseline,” “ambiguous decision-making task>certain decision-making task,” “ambiguous decision-making task>baseline”; (1a)*task engagement when only experiments using a low-level control condition were considered*—studies investigating experimental decision-making task as compared with an implicit or explicit baseline condition, for example, guided repetition of the main experiment, or unguided motorvisual task, or a resting-state scan as determined by contrasts “task of interest>(implicit or explicit) baseline,” for example, “moral decision-making task>baseline,” “risky decision-making task>baseline,” “ambiguous decision-making task>baseline”;(1b)*task engagement when only experiments using a high-level control condition were considered*—studies investigating domain-specific effects by comparing experimental decision-making task with a control decision-making task from a different or opposite domain as determined by contrasts “task of interest>control task,” for example, “moral decision-making task>non-moral decision-making control task,” “risky decision-making task>safe decision-making task,” “ambiguous decision-making task>certain decision-making task”; and (2)*choice response*—studies investigating subjective aspects of participants’ choice preference as determined by the contrasts “chosen option of interest>opposite chosen option,” “chosen option>(implicit or explicit) baseline,” for example, “utilitarian choice>deontological choice” or “prosocial choice>selfish choice” for the morality domain, “risky choice>safe choice” for the risk domain, and “ambiguous choice>certain choice” for the ambiguity domain.

To mirror the choice content from the risk and ambiguity domains, that is, choices of higher possible outcome magnitude and higher uncertainty compared with choices of lower possible outcome magnitude but higher or complete certainty, in the morality domain we selected contrasts comparing moral choices that involve larger benefit to others or oneself but also higher social uncertainty with moral choices that involve smaller benefit to others or oneself but with higher social certainty.

In studies of morality or prosociality, participants choose from two options with different outcomes according to what they consider morally permissible. One option usually involves a better outcome to a larger amount of people (be it sacrificing somebody to save a larger number of people, that is, an utilitarian choice, or foregoing selfish needs and donate to charity, while the other option involves better outcome to a smaller number of people (not using a bystander as collateral damage, thus allowing a larger number of people to be killed, i.e., a deontological choice or fulfilling one’s selfish needs,Hutcherson et al., 2015;Moll et al., 2006). When these two options are compared, the first option requires acting proactively (Gawronski et al., 2017), facing possible social uncertainty (Kahane et al., 2012), and achieving a more quantitatively rewarding outcome. The second option, on the other hand, could be regarded as a “safe” option, as it requires less or no active participation in the situation (Gawronski et al., 2017), and involves no or at least less moral uncertainty regarding the consequences for oneself (Kahane et al., 2012).

In prosocial or altruistic decision-making tasks, the “safe” option and the direction of the contrast included were considered individually depending on the task content. In tasks that simulated donations to charity, we considered that a more uncertain choice was the prosocial one, that is to donate money to charity, while a more certain “safe” choice was to abstain from donation or keep the benefit to oneself as previous research has shown a negative association between uncertainty avoidance and charitable giving (Stojcic et al., 2016). Contrasts comparing charitable giving with a decision not to give or keep the money for oneself were classified as “altruism” in task subcategorization. In other cases, an opposite contrast was more in line with our research question. That is, in some specific cases, for example, the Trust game or Dictator game, a choice to divide the money equally was considered a “safe” choice option because this type of choice does not elicit third-party (altruistic) punishment (Fehr & Fischbacher, 2004), while the selfish choice was considered an uncertain one. In these cases, contrasts comparing selfish decisions with prosocial decisions were classified as “harm” in the task subcategorization for the morality domain.

Differentiating options according to the magnitude of the possible benefit to others and possible moral uncertainty is akin to the differentiation of risky and ambiguous choice options as opposed to a safe option. In the risk domain, contrasts comparing risky choice as opposed to a safe choice or less risky choice were selected. Similarly, in the ambiguity domain, contrasts comparing ambiguous choice as opposed to safe or less ambiguous choice were included. Direct contrasts comparing risky and ambiguous choices were not included in this meta-analysis to avoid biasing results towards domain differences.

A licensed software EndNote 20 (Clarivate Analytics, USA) was used for storing manuscripts, removing duplicates, screening, and full-text review. Data were extracted by hand into a database, based on a Microsoft Excel file, and double-checked by an independent investigator. Extracted Talairach coordinates were transformed into the MNI coordinate space by using a linear transformation (Lancaster et al., 2007). When the coordinate system was not explicitly stated, coordinates from experiments which used SPM or FSL software were treated as MNI coordinates.

### 2.2 ALE meta-analysis

All meta-analyses were performed according to the standard analysis procedure used in previous studies (cf.Kogler et al., 2020;Müller, Höhner, et al., 2018). In particular, to identify consistent brain activation across the experiments, coordinate-based analyses were performed using the revised Activation Likelihood Estimation (ALE) algorithm (Eickhoff et al., 2012) implemented as an in-house Matlab code, using Matlab 2019b software (The Mathworks, Inc, USA). The ALE algorithm identifies clusters that show convergence of coordinates across experiments that is significantly higher than it would be expected under a random spatial distribution (Eickhoff et al., 2009,^2012^). Importantly, this algorithm treats all reported activation foci as centres of 3D Gaussian probability distributions and not as single points, therefore, modelling the spatial uncertainty of neuroimaging results. The width of the probability function is determined by the between-template and between-subjects variance, with the between-subjects variance being weighted by the number of participants for each experiment. That is, experiments with more participants should provide more precision of the true location and are, therefore, modelled by smaller Gaussian distributions.

The probabilities of all foci reported in each study were combined for each voxel, resulting in a modelled activation (MA) map for each experiment (Turkeltaub et al., 2012). To ensure that the meta-analytical results were not driven by studies reporting more than one contrast, different contrasts from the same study were coded as one experiment if included in the same meta-analysis. Unifying the MA maps of each study resulted in voxel-wise ALE scores indicating the convergence across results at a particular location of the brain. These scores were then compared with an analytically derived null distribution of random spatial associations between experiments (Eickhoff et al., 2012). As suggested for ALE, cluster-level family-wise error (FWE) corrected threshold of p < 0.05 and cluster-forming threshold at voxel-level p < 0.001 with 10,000 permutations were used to correct for multiple comparisons (Eickhoff et al., 2016;Frahm et al., 2022). The requirement of a minimum number of 17 experiments per analysis was set to have sufficient power to make sure that the results are not driven by a single experiment as well as to identify smaller effects (Müller, Cieslik, et al., 2018). Where necessary, separate experiments from the same sample were treated as one experiment, depending on combinations of domains and/or*task engagement*and*choice response*categories.

Contributions of each experiment and for labels (type of task, domain) were calculated as the ratio of the ALE value with and without the experiment (label) in question to rule out that convergence is driven by only few experiments (seeSupplemental Tables 7 and 8for detailed lists of experiment contributions andFigs. 2and4for aggregated contributions by task type).

**Fig. 2.**
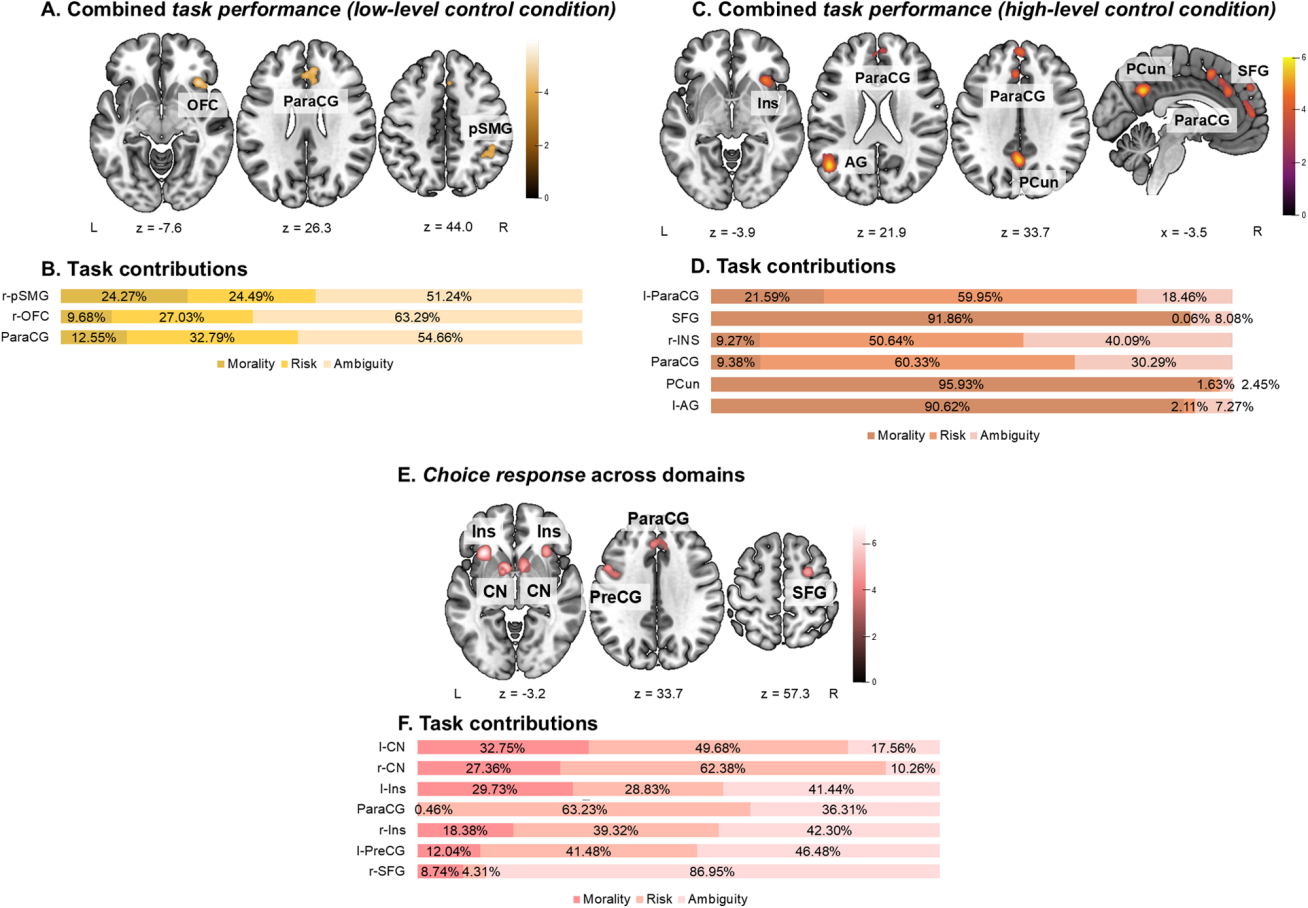
Results of ALE meta-analysis in task engagement category when only experiments with a low-level control condition were considered, task engagement category when only experiments with a high-level control condition were considered, and the choice response category. (A) Convergence of activity in task engagement when only experiments with low-level control condition were considered across all three decision-making domains. (B) Task contributions (experiments from the morality, risk, and ambiguity domains) by percent in task engagement (low-level control condition) category. (C) Conjunction and contrasts between meta-analytical results of*task engagement (low-level control condition)*and*task engagement (high-level control condition)*. (D) Task contributions (experiments from the morality, risk, and ambiguity domains) by percent in task engagement (high-level control condition) category. (E) Convergence of activity in*choice response*category across all three decision-making domains. (F) Task contributions (experiments from the morality, risk, and ambiguity domains) by percent in choice response category. Meaning of abbreviations: bil—bilateral, r—right, l—left, OFC—orbitofrontal cortex, ParaCG—paracingulate gyrus, pSMG—posterior division of supramarginal gyrus, Ins—insula, AG—angular gyrus, PCun—precuneus, SFG—superior frontal gyrus, CN—caudate nucleus, PreCG—precentral gyrus, lOCC—lateral occipital cortex, NAcc—nucleus accumbens.

Conjunction and contrast analyses were performed between the decision-making domains and*task engagement*and*choice response*categories. For conjunctions, a conservative minimum statistic was applied, so that only regions which reached statistical significance in the multiple-comparison corrected meta-analysis were considered (Nichols et al., 2005). Differences between the task and choice response as well as the three decision-making domains within each analysis angle were calculated by comparing the difference between two ALEs to a random distribution (based on 25,000 permutations) of differences (Eickhoff et al., 2012). Contrasts were masked by the respective main effects; an additional cluster threshold of 10 voxels was applied to contrast and conjunction analyses (Lieberman & Cunningham, 2009).

Anatomical labelling of meta-analytical results was performed with JuBrain Anatomy Toolbox Version 3.0 (Eickhoff et al., 2005,^2006^,^2007^) and MRIcron Version 1.0.20190902 (Rorden & Brett, 2000). Illustrations of the meta-analytical results were generated with MRIcroGL Version 20 (Rorden & Brett, 2000).

### 2.3 Functional decoding

Each statistically significant cluster of convergent activation resulting from meta-analyses in*task engagement (combined)*across the domains,*choice response*across the domains,*task engagement (combined)*in the morality domain,*task engagement (combined)*in the risk domain,*task engagement (combined)*in the ambiguity domain, as well as*choice response*in the morality domain, and*choice response*in the risk domain was functionally characterized by using the meta-data from the BrainMap database (Laird et al., 2009,^2013^). The analysis was performed with in-house scripts in the MatLab 2016 software (The Mathworks, Inc, USA) on 7937 published human neuroimaging studies (samples included healthy adults only) in the BrainMap database. Each cluster of convergent activation from the meta-analytical maps was separated into single region-of-interest map. This map was then used for the forward and reverse inference functional association analyses (Langner & Eickhoff, 2013; see alsoPoldrack, 2011). The BrainMap database contains coordinates of published neuroimaging studies which utilized statistical parametric images to identify regionally specific experimental task effects. The reported three-dimensional coordinates in a normalized reference space are assigned to the meta-data in the BrainMap database, which includes 5 main behavioural domains (cognition, action, perception, emotion, and interoception) and over 50 behavioural subdomains determined by the condition comparison in the included studies, as well as paradigm classes labelling the experimental task used in the experiment.

During the functional decoding analysis, forward and reverse inferences allow us to identify what experimental meta-data are associated with a particular region of interest, indicating what behavioural domains can be expected to evoke activation in that region of interest (forward inference,Henson, 2005,^2006^) or what specific mental state can be expected to be engaged by that cluster of activation (reverse inference,Poldrack, 2011). During the forward inference analysis, we tested whether the probability of activation in clusters of interest given a particular behavioural process [P(Activation|Task)] was higher than the baseline probability of activation in those clusters across the entire database [P(Activation)]. A binomial test estimated significance at p < 0.05, corrected for multiple comparisons using false discovery rate (FDR). In the reverse inference analysis, the likelihood P(Task|Activation) was derived from P(Activation|Task) as well as P(Task) and P(Activation) using Bayes’ rule, and the significance was set by chi-square tests at p < 0.05 and corrected for multiple comparisons using FDR.

## 3 Results

### 3.1 Literature search and data availability for analyses

In total, 149 studies met inclusion criteria for the current meta-analysis, providing 164 experiments from 152 independent samples (details on first author, publication year, number of participants, average age of participants, assigned decision-making domain, assigned task category, task description, originally reported contrast name and source table, number of foci, imaging method, and originally reported coordinate system can be found inSupplementary Table 2). The included experiments examined 3,625 healthy adult subjects overall. Eleven studies reported separate experiments, assessing task engagement and choice response (Abe et al., 2014;Ernst et al., 2004;Gloy et al., 2020;Han et al., 2014;Kireev et al., 2013;Lawrence et al., 2009;Sommer et al., 2010,^2014^;Vassena et al., 2014;Vorobyev et al., 2015;Wiehler et al., 2021), and one study used two separate experimental tasks with one being categorized as risk domain and the other as ambiguity domain (Blankenstein et al., 2017).

Ninety-five studies (99 experiments) were assigned to the *task engagement* category:

- in the morality domain, 46 studies (48 experiments: 41 experiments with high-level control condition involving decision-making, 4 experiments with low-level visuomotor control condition, 3 experiments with resting-state or fixation baseline as control condition; 471 foci in total) were included;
- in the risk domain, 23 studies (24 experiments: 9 experiments with high-level control condition involving decision-making, 11 experiments with low-level visuomotor control condition, and 4 experiments with resting-state or fixation baseline as control condition; in total 434 foci) were included;
- and in the ambiguity domain, 27 studies (27 experiments: 13 experiments with high-level control condition involving decision-making, 8 experiments with low-level visuomotor control condition, and 6 experiments with resting-state baseline as control condition; in total 454 foci) were included.

*The task engagement* category across the domains was further separated into:

- task engagement only including experiments which used a low-level control condition (35 experiments: 7 experiments from the morality domain, 14 experiments from the risk domain, and 13 experiments from the ambiguity domain, and 1 experiment where coordinates from risk and ambiguity domains had to be considered as a single experiment due to originating from the same sample, 596 foci in total);
- task engagement only including experiments which used a high-level control condition (63 experiments: 41 experiments from the morality domain, 9 experiments from the risk domain, 13 experiments from the ambiguity domain, 760 foci in total).

Differentiation between task engagement categories separately for low-level and high-level control conditions in each of the decision-making domains was not possible due to the low number of experiments using a low-level control condition in all three domains, and a low number of experiments using a high-level control condition in the risk and ambiguity domains (per best practice recommendations, minimum requirement for ALE meta-analysis is 17–20 experiments, seeMüller, Cieslik, et al., 2018).

Sixty-five studies (65 experiments) were assigned to *the choice response* category:

- in the morality domain, 20 studies (20 experiments: all 20 experiments comparing a choice of higher outcome magnitude and higher moral/probabilistic uncertainty with a choice of lower outcome magnitude and higher moral/probabilistic certainty, in total 192 foci)
- in the risk domain, 32 studies (32 experiments: 29 experiments comparing a choice of higher outcome magnitude and higher probabilistic uncertainty (risky choice) with a choice of lower outcome magnitude and higher/complete probabilistic certainty (safe choice), 2 experiments comparing a choice of higher outcome magnitude and higher probabilistic uncertainty (risky choice) with a guided motorvisual condition, and 1 experiment comparing a choice of higher outcome magnitude and higher probabilistic uncertainty (risky choice) with a resting-state baseline, in total 238 foci), and
- in the ambiguity domain, 13 studies (13 experiments: all 13 experiments comparing a choice of higher outcome magnitude and complete probabilistic uncertainty (ambiguous choice) with a choice of lower outcome magnitude and higher/complete probabilistic certainty (safe choice), in total 162 foci).

ALE meta-analyses were performed for:

1. Main*task engagement*effect across all domains (N = 98),
2. Main*task engagement*effect when only experiments with low-level control condition were considered across all domains (N = 35),
3. Main*task engagement*effect when only experiments with high-level control condition were considered across all domains (N = 63),
4. Main*choice response*effect across all domains (N = 65),
5. Morality domain and*task engagement (combined)*category (N = 48),
6. Risk domain and*task engagement (combined)*category (N = 24),
7. Ambiguity domain and*task engagement (combined)*category (N = 27),
8. Morality domain and*choice response*category (N = 20),
9. Risk domain and*choice response*category (N = 32).

Not enough experiments were detected for the ambiguity domain in the*choice response*category (N = 13) to perform a meta-analysis (per best practice recommendations, minimum requirement for ALE meta-analysis is 17–20 experiments, seeMüller, Cieslik, et al., 2018).

Additionally, further ALE meta-analyses were performed and reported in the supplement:

1. Uncertainty domain and*task engagement*: experiments from risk (24 experiments) and ambiguity (27 experiments) domains for the*task engagement*category were combined to investigate activation convergence in the uncertainty domain (N = 51 experiment) (seeSupplementary Table 3).
2. Uncertainty domain and*choice response*: experiments from risk (N = 32) and ambiguity (N = 13) were combined to investigate activation convergence in the uncertainty domain (N = 44 experiments) (seeSupplementary Table 3).
3. Experiments from morality, risk, and ambiguity domains and both*task engagement*and*choice response*categories (N = 153) were combined to assess activation convergence for all included experiments in the meta-analysis (seeSupplementary Table 4).
4. Experiments from*task engagement*and*choice response*categories in morality (N = 64), risk (N = 52), and ambiguity (N = 38) domains separately were combined to investigate general domain effects (seeSupplementary Table 5).

### 3.2 ALE meta-analysis results

#### 3.2.1 Task engagement *across domains*

To investigate consistent activation across experiments in*task engagement*across decision-making domains, we performed several ALE meta-analyses. In*task engagement,*we have additionally separated experiments into further categories depending on what control condition they used for condition comparison, that is, low-level control condition where the control task required no decision-making from the participants, for example, resting-state baseline or motorvisual guided repetition of the main experiment, or a high-level control condition where decision-making behaviour was required of participants during the control task. We also investigated consistent activation across the experiments when both low- and high-level control conditions were used.

First, we were interested in consistent activation across experiments across the morality, risk, and ambiguity domains, which utilized a low-level control condition in their condition comparison. Such condition comparison strategy allows researchers to investigate neural correlates of both domain-general and domain-specific decision-making processes. These analyses (35 experiments, 759 unique subjects) revealed that consistent activation is found in the paracingulate and cingulate gyri (located in dorsal anterior cingulate cortex (dACC),Heilbronner & Hayden, 2016), right orbitofrontal cortex (OFC), and right posterior supramarginal gyrus (pSMG, in the parietal cortex, (Wild et al., 2017), for the list of clusters and cluster maxima coordinates, seeTable 1AandFig. 2A, B). Task contributions (seeSupplementary Table 6) revealed that these results were primarily driven by the gambling tasks in the risk and ambiguity domains, as well as guessing tasks in the ambiguity domain. Two experiments from the morality domain (one using a deception task and one using moral dilemmas) also contributed to the pSMG cluster of convergent activation.

**Table 1.** Shows coordinates of peak convergence of all clusters identified in the ALE analysis of*task engagement (low-level control conditions), task engagement (high-level control conditions), and task engagement (combined)*, respectively, across all three domains.

Cluster #	Hemisphere	Region	BA	Z-value (uncorrected)	Size (Voxels)	x	y	z
***A. All studies in task engagement (low-level control conditions only)***								
1	R/L	Paracingulate gyrus, anterior division of cingulate gyrus	32	5.21	294	6	28	34
2	R	Orbitofrontal cortex	-	4.22	228	40	22	-2
3	R	Posterior division of supramarginal gyrus	40	3.79	121	46	-48	42
***B. All studies in task engagement (high-level control conditions only)***								
1	L	Angular gyrus, superior division of Lateral occipital cortex	39	6.07	291	-48	-64	24
2	R/L	Precuneus cortex, posterior division of cingulate cortex	-	6.04	280	-2	-56	32
3	R/L	Paracingulate gyrus, anterior division of cingulate gyrus	32	4.66	219	-6	30	30
4	R	Insular cortex	47	4.91	210	32	22	-6
5	R/L	Superior frontal gyrus, Paracingulate gyrus	9	4.53	206	0	52	34
6	L	Paracingulate gyrus	32	5.28	99	-6	14	50
***C. All studies in task engagement (combined)***								
1	R/L	Paracingulate gyrus	32	5.15	696	4	24	42
2	R	Orbitofrontal cortex	-	5.93	448	34	22	-8
3	L	Angular gyrus	39	5.76	304	-48	-62	24
4	L	Insular cortex	48	4.91	249	-34	18	4
5	R/L	Precuneus	-	6.02	249	-2	-54	32
6	R	Thalamus	-	5.92	210	10	-16	4
7	R	Frontal pole	45	5.43	188	42	36	22
8	L	Superior parietal lobule	7	5.75	187	-28	-58	48
9	R	Superior division of lateral occipital cortex	7	4.35	125	34	-58	50
***D. Conjunction task engagement (low-level control conditions only) ∩ task engagement (high-level control conditions only)***								
1	R	Orbitofrontal cortex	47	4.14	67	36	24	-6
2	R	Paracingulate gyrus	32	3.62	13	4	24	44
***E. Contrast task engagement (low-level control conditions only) > task engagement (high-level control conditions only)***								
1	R	Paracingulate gyrus	32	2.95	154	10	26	30
2	R	Orbitofrontal cortex	-	2.97	90	36	26	-14
3	R	Angular gyrus	40	3.18	87	48	-50	50
***F. Contrast task engagement (high-level control conditions only) > task engagement (low-level control conditions only)***								
1	L	Superior division of lateral occipital cortex	39	2.94	114	-52	-66	18
2	R/L	Precuneus	-	2.81	84	-4	-56	38
3	R/L	Frontal pole	10	2.32	28	-2	56	6

Visualization of the results is shown inFigures 2and3A. Experiment contribution details are given inSupplementary Table 6. Hemisphere, region, Broadman area (BA), Z-value, size in voxels, and MNI coordinates are listed.

*Note:*R, right; L, left; R/L, medial.

Then we assessed only those experiments across all decision-making domains, which utilized a high-level control condition in their condition comparison strategy (63 experiments, 1,353 unique subjects). This condition comparison strategy is used when only domain-specific neural activation is of interest, mostly representing processing of specific contextual cues for that domain. Here the control condition usually involves decision-making behaviour only in a “neutral” context. The meta-analysis identified significant activity convergence in six cortical clusters in total (seeTable 1B). The clusters in the left angular gyrus (AG), precuneus, and superior frontal gyrus (SFG) extending into the paracingulate gyrus (ParaCG) were primarily driven by the experiments in the morality domain (mostly moral dilemma tasks, seeFig. 2C–DandSupplementary Table 8). The two clusters in the ParaCG (anterior and dorsal ACC) and a cluster in the right anterior insula (aINS) were driven by experiments in the risk and ambiguity domains (seeFig. 2C–DandSupplementary Table 8).

The conjunction analysis indicated that the two analysis categories reported above share consistent activation in the right insula and in the right ParaCG (seeTable 1DandFig. 3A). Contrast analyses indicate that*task engagement*when only low-level control conditions are applied has higher convergence of activations in the right OFC and the right ParaCG clusters than*task engagement*when only high-level control conditions are used, closely neighbouring the clusters of shared spatial convergence of the two analysis categories. A cluster in the right AG (located in the parietal cortex,Wild et al., 2017) is related only to*task engagement*when only low-level control conditions are used (seeTable 1EandFig. 3A). The opposite contrast revealed that clusters of convergent activation in the left lateral occipital cortex (lOCC), precuneus, and frontal pole (FP), all mostly driven by experiments in the morality domain, are more consistently activated in*task engagement*when only experiments with only high-level control conditions are considered than in*task engagement*when only experiments with low-level control conditions are grouped (seeTable 1FandFig. 3A).

**Fig. 3.**
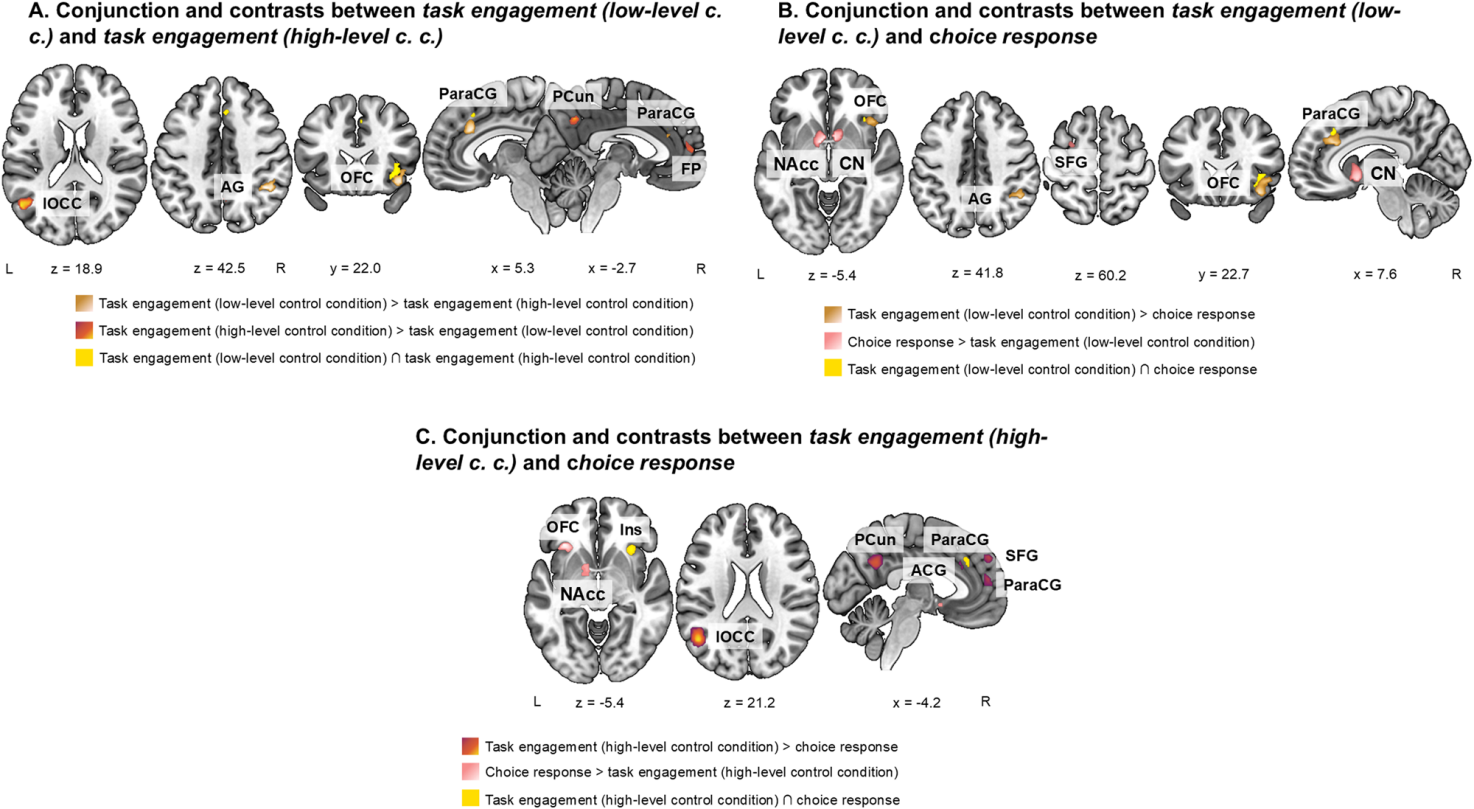
Results of conjunction and contrast analyses. (A) Conjunction and contrasts between meta-analytical results of*task engagement (low-level control condition)*and*task engagement (high-level control condition)*. (B) Conjunction and contrasts between meta-analytical results of*task engagement (low-level control condition)*and*choice response*. (C) Conjunction and contrasts between meta-analytical results of*task engagement (high-level control condition)*and*choice response*. Meaning of abbreviations: c. c.—control condition, R—right, L—left, OFC—orbitofrontal cortex, ParaCG—paracingulate gyrus, pSMG—posterior division of supramarginal gyrus, Ins—insula, AG—angular gyrus, PCun—precuneus, SFG—superior frontal gyrus, CN—caudate nucleus, lOCC—lateral occipital cortex, FP—frontal pole, NAcc—nucleus cccumbens, ACG—anterior cingulate gyrus.

Combining experiments using low-level and high-level control conditions across domains in the*task engagement*analysis category reveals similar patterns of activation as in the two analyses reported above (for complete list of clusters and cluster maxima coordinates, seeTable 1C; for task contributions seeSupplementary Table 6; for result visualization seeSupplementary Fig. 1). The combination of experiments that use low- and high-level control conditions allows us to capture domain-specific activity convergence more reliably as both of these analysis categories involve increased domain-specific activation as compared with the control condition. Besides the already identified clusters of convergent activation in the separate meta-analyses, additional clusters in this combined meta-analysis were revealed in the right FP, left aINS, left superior parietal lobule (SPL), and right superior part of lOCC, all primarily driven by experiments from the risk and ambiguity domains, and the right thalamus mostly driven by experiments from the ambiguity domain (for task contributions, seeSupplementary Table 6andSupplementary Fig. 1).

#### 3.2.2 Choice response *across domains*

To investigate choice-specific effects on neural activation, we combined all experiments (64 experiments, 1,703 unique subjects) in the*choice response*analysis category, in which experiments have compared chosen options with higher possible outcome magnitude but also higher uncertainties (probabilistic and/or social) to chosen options of lower possible outcome magnitude and higher or complete certainty across the morality, risk, and ambiguity domains. Our analysis revealed that a higher possible outcome magnitude and higher uncertainty of the selected choice option result in consistent involvement of clusters in the right and left caudate nuclei (CN), right and left aINS extending into OFC on the left, a medial cluster in SFG extending into ParaCG (located in dACC,Wild et al., 2017), a cluster in the left precentral gyrus (PreCG) extending into medial frontal gyrus (MFG), and a cluster in the right SFG extending into MFG (seeTable 2AandFig. 2E–F). Experiments contributing to these clusters primarily conducted risky or ambiguous decision-making tasks, however, interestingly experiments from the morality domain contributed almost a third to the bilateral CN and left aINS clusters (seeFig. 2FandSupplementary Table 7).

**Table 2.** Shows coordinates of peak convergence of all clusters identified in the ALE analysis of*choice response*across all three domains.

***A. All studies in choice response***								
1	L	Caudate	25	6.27	328	-12	8	-4
2	R	Caudate	25	6.00	260	10	10	0
3	L	Insular cortex	47	6.97	243	-32	24	-2
4	R/L	Paracingulate gyrus	32	4.83	198	8	34	38
5	R	Insular cortex	47	5.35	183	32	24	-2
6	L	Precentral gyrus	44	4.60	174	-50	10	34
7	R	Superior frontal gyrus	6	4.99	114	26	0	58
***B. Conjunction choice response ∩ task engagement (combined)***								
1	R	Insular cortex	47	5.05	157	34	24	0
2	L	Insular cortex	47	4.21	92	-30	20	0
3	R/L	Paracingulate gyrus	32	4.02	66	6	32	38
***C. Conjunction choice response ∩ task engagement (low-level control conditions only)***								
1	R	Orbitofrontal cortex	47	3.82	36	38	22	0
2	R	Paracingulate gyrus		3.88	23	8	30	36
***D. Conjunction choice response ∩ task engagement (high-level control conditions only)***								
1	R	Insular cortex	47	4.71	124	32	22	-4
2	L	Paracingulate gyrus	32	4.00	27	-4	32	34
***E. Contrast choice response > task engagement (combined)***								
1	L	Caudate	-	2.97	236	-10	-2	-6
2	R	Caudate	-	2.22	112	12	12	6
3	L	Insular cortex, orbitofrontal cortex	47	2.27	65	-36	24	-2
4	R	Superior frontal gyrus	6	2.67	46	24	-2	62
***F. Contrast task engagement (combined) > choice response***								
1	L	Angular gyrus	39	5.53	299	-48	-60	24
2	R/L	Precuneus	-	3.60	201	-4	-56	32
3	R/L	Anterior division of cingulate gyrus	24	3.60	161	2	28	22
4	R	Thalamus	-	2.61	128	10	-16	8
5	R	Orbitofrontal cortex	38	2.53	75	38	20	-16
6	L	Superior division of lateral occipital cortex	7	2.52	63	-34	-60	46
7	R	Superior division of lateral occipital cortex	7	1.91	10	34	-62	48
***G. Contrast choice response > task engagement (low-level control conditions only)***								
1	L	Nucleus accumbens	25	3.49	192	-6	4	-8
2	R	Nucleus accumbens, caudate	25	3.24	154	4	6	-10
3	R	Superior frontal gyrus	6	1.84	19	26	2	60
***H. Contrast task engagement (low-level control conditions only) > choice response***								
1	R	Anterior division of cingulate gyrus	24	3.53	195	4	28	28
2	R	Orbitofrontal cortex	38	3.20	160	36	24	-16
3	R	Angular gyrus	40	3.01	61	46	-50	44
***I. Contrast choice response > task engagement (high-level control conditions only)***								
1	L	Nucleus accumbens, pallidum	34	2.39	138	-16	0	-12
2	L	Orbitofrontal cortex	47	2.57	116	-32	26	-4
3	R	Superior frontal gyrus	6	2.18	23	24	-2	62
4	R	Superior frontal gyrus	32	1.92	10	10	36	40
***J. Contrast task engagement (high-level control conditions only) > choice response***								
1	L	Superior division of lateral occipital cortex, angular gyrus	39	6.04	284	-48	-62	24
2	R/L	Precuneus, posterior division of cingulate gyrus	-	3.78	253	-4	-54	32
3	R/L	Superior frontal gyrus	9	3.13	55	-4	54	38
4	L	Paracingulate gyrus	32	3.14	48	-2	52	16
5	L	Paracingulate gyrus	32	2.09	18	-8	12	44
6	L	Anterior division of cingulate gyrus	24	2.09	11	-4	28	26

Visualization of the results is shown inFigures 2Eand3B, C. Experiment contribution details are shown inFigure 2FandSupplementary Table 7. Hemisphere, region, Broadman area, Z-value, size in voxels, and MNI coordinates are listed.

*Note:*R, right; L, left; R/L, bilateral.

We have also investigated the similarities and differences of convergence of activations between the*task engagement*analysis category, during which the BOLD signal recorded during the task is unbroken and compared with the BOLD signal obtained during a control condition, and*choice response*analysis category, during which the BOLD signals are split apart and differentiated according to the actual choices of participants and later on are directly compared. In the conjunction analyses (seeTable 2BandSupplementary Fig. 2), we found that*task engagement*(experiments with high- and low-level control tasks combined) and*choice response*share spatial convergence of activations in clusters in the bilateral aINS extending to OFC on the left and in the medial ParaCG (located in dACC,Wild et al., 2017). Comparing*choice response*with*task engagement*divided according to a low- or high-level control task used in the condition comparison revealed a particular lateralization and cluster parcellation of the identified clusters.*Choice response*shares consistent involvement of clusters in the right OFC and the right ParaCG (located in dACC,Wild et al., 2017) with*task engagement*when only experiments with low-level control condition are considered (seeTable 2CandFig. 3B), while with*task engagement*when only experiments with a high-level control condition are considered, it shares convergence of activations in the right aINS and the cluster in the ParaCG (located in dACC,Wild et al., 2017) on the left hemisphere (seeTable 2DandFig. 3C). We have additionally performed a functional decoding analysis to confirm the functional associations of identified clusters in*task engagement (combined)*and*choice response categories*(seeSupplementary Fig. 3).

Investigating differences between the analysis categories showed that convergence of activations in the nucleus accumbens (NAcc) bilaterally (with an exception for comparison with*task engagement*when only experiments with low-level control tasks are considered), in the SFG, and a part of the cluster in the left aINS extending into OFC can be only captured by the*choice response*analysis approach (seeTable 2E, G, IandFig. 2E–F). Contrasts in the opposite direction indicated that in*task engagement (combined)*, convergent activation accumulates in clusters driven by domain-specific contributions from experiments that use a high-level control task: two clusters in the left AG and precuneus driven by experiments from the morality domain, three clusters in the anterior cingulate gyrus (ACG), right OFC, and the left superior part of lOCC driven by experiments from the risk and ambiguity domains, one cluster in the right superior part of lOCC driven by experiments from the risk, and one cluster in the right thalamus, primarily driven by experiments from the ambiguity domain (for complete list of clusters see,Table 2F). This was confirmed by comparison of*task engagement*when only experiments with high-level control tasks were considered with*choice response*as a similar set of clusters was revealed by a contrast analysis (for complete list of clusters, seeTable 2JandFig. 3C). Comparison of*task engagement*when experiments with low-level control tasks only were considered with*choice response*revealed specific parcellations of the right OFC and ParaCG clusters neighbouring the shared consistent activation clusters, and a cluster in the right AG (seeTable 2HandFig. 3B).

#### 3.2.3 *Moral, risky, and ambiguous decision-making in* task engagement

To further investigate how a specific domain (i.e., contextual cues) influences what brain networks are involved in decision-making processing and whether there are any similarities or differences in convergence of activations between the morality, risk, and ambiguity domains, we performed separate ALE meta-analyses in the*task engagement*category for each domain. For these analyses, we have combined experiments comparing moral, risky, or ambiguous decision-making tasks with both low- and high-level control conditions, that is, either requiring no decision-making or requiring decision-making in the control condition, to achieve more power in the statistical analyses. Both low- and high-level control conditions involve domain-specific processes, so in our results, we expected to see consistent involvement of brain areas engaged in domain-specific cue processing. To confirm that our results indeed correspond to domain-specific cue processing, we report experiment contributions (percentage of contribution from experiments with low- and high-level control condition, also seeSupplementary Table 8). We have additionally performed a functional decoding analysis to confirm the domain-general and domain-specific functional associations of identified clusters (seeSection 3.3.).

When moral decision-making was considered in the category of*task engagement*(48 experiments, 1,062 unique subjects), our analysis resulted in 4 clusters (seeTable 3AandFig. 4A) in the medial SFG extending into ParaCG (located in the dorsomedial prefrontal cortex (dmPFC),Eickhoff et al., 2014), left AG extending into superior division of the lOCC, medial precuneus extending into posterior cingulate gyrus (PCG), and the right temporal pole (TP). All clusters were mainly driven by experiments of moral dilemmas dealing with either morally relevant harm or sacrificial harm (seeFig. 4B) that were contrasted against a high-level (non-moral decision-making) control condition (seeSupplementary Table 8). When compared with the risk and ambiguity domains, both conjunction and contrast analyses revealed no significant overlaps between the domains (seeTable 3D, G), while domain differences closely mirrored the main results of the ALE meta-analyses in the specific domains (for a full list of clusters in contrast analyses, seeTable 3E, F, H, I).

**Fig. 4.**
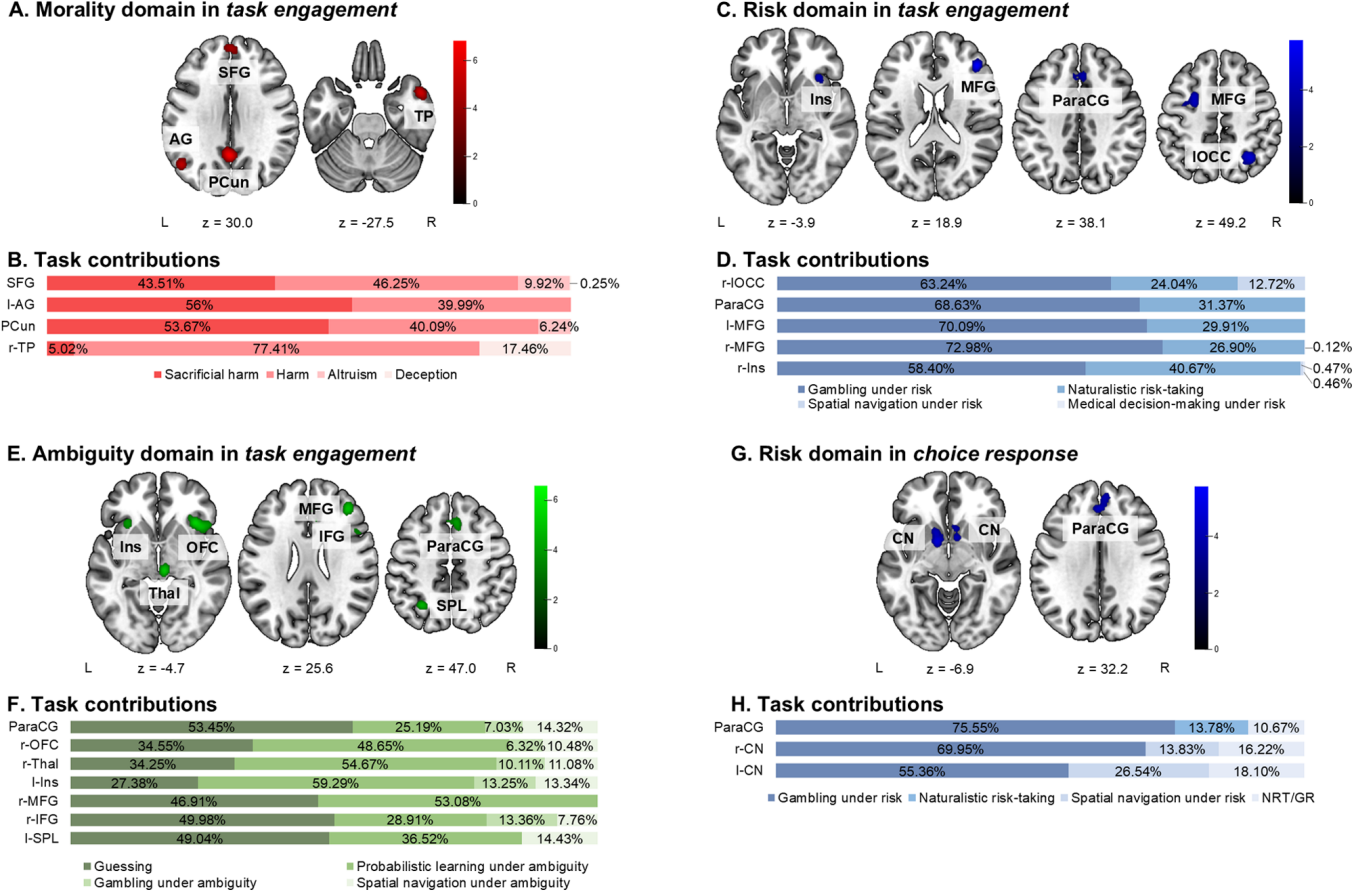
Results of ALE meta-analysis across experiments of the morality, risk, and ambiguity domains in task engagement and choice response categories. (A) Clusters of convergent activity in the morality domain in*task engagement*category. (B) Task contributions (sacrificial harm, altruism, and deception) by percent in the morality domain in task engagement category. (C) Clusters of convergent activity in the risk domain in*task engagement*category. (D) Task contributions (gambling under risk, naturalistic risk-taking, spatial navigation under risk, medical decision-making under risk) by percent in the risk domain in*task engagement*category. (E) Clusters of convergent activity in the ambiguity domain in*task engagement*category. (F) Task contributions (guessing, probabilistic learning under ambiguity, gambling under ambiguity, and spatial navigation under ambiguity) by percent in the ambiguity domain in*task engagement*category. (G) Clusters of convergent activity in the risk domain in*choice response*category. (H) Task contributions (gambling under risk, naturalistic risk-taking, spatial navigation under risk, NRT/GR—combined results from naturalistic risk-taking and gambling under risk task are given inPletzer & Ortner, 2016) by percent in ambiguity domain in*task engagement*category. Meaning of abbreviations: m—medial, r—right, l—left, SFG—superior frontal gyrus, AG—angular gyrus, PCun—precuneus, TP—temporal pole, Ins—insula, MFG—medial frontal gyrus, ParaCG—paracingulate gyrus, lOCC—lateral occipital cortex, OFC—orbitofrontal cortex, Thal—thalamus, IFG—inferior frontal gyrus, SPL—superior parietal lobule, CN—caudate nucleus, F-I—forward inference, R-I—reverse inference.

**Table 3.** Shows peak coordinates of the clusters of significant convergence resulting from the ALE analysis of*task engagement (combined)*for each domain separately.

Cluster #	Hemisphere	Region	BA	Z-value (uncorrected)	Size (Voxels)	x	y	z
***A. Morality in task engagement***								
1	R/L	Superior frontal gyrus	9	5.38	517	0	52	34
2	L	Angular gyrus	39	6.76	509	-48	-64	24
3	R/L	Precuneus	-	6.88	384	-2	-56	32
4	R	Temporal pole	21	5.50	157	52	8	-26
***B. Risk in task engagement***								
1	R	Superior division of lateral occipital cortex	7	4.78	196	32	-58	50
2	R/L	Paracingulate gyrus	32	4.19	169	4	20	44
3	L	Middle frontal gyrus	6	4.42	155	-26	4	50
4	R	Middle frontal gyrus	45	5.80	131	44	36	20
5	R	Insular cortex	47	4.31	126	32	22	-8
***C. Ambiguity in task engagement***								
1	R/L	Paracingulate gyrus	32	4.78	382	4	24	44
2	R	Orbitofrontal cortex	47	5.64	367	36	24	-6
3	R	Thalamus	-	6.61	315	10	-16	4
4	L	Insular cortex	47	4.74	147	-30	22	0
5	R	Middle frontal gyrus	46	5.05	108	40	38	26
6	R	Inferior frontal gyrus	48	4.34	104	46	18	30
7	L	Superior parietal lobule	7	4.78	104	-28	-58	46
***D. Conjunction morality ∩ risk in task engagement***								
		*No significant results*						
***E. Contrast morality > risk in task engagement***								
1	R/L	Superior frontal gyrus	9	4.25	372	-8	54	36
2	L	Angular gyrus	39	3.94	362	-48	-62	18
3	R/L	Precuneus	-	3.49	288	-4	-58	36
4	R	Temporal pole	21	2.18	119	48	8	-28
***F. Contrast risk > morality in task engagement***								
1	R	Superior division of lateral occipital cortex	7	4.64	189	32	-60	50
2	R/L	Anterior division of cingulate gyrus	32	4.13	164	4	20	42
3	L	Superior frontal gyrus	6	4.32	155	-24	-4	50
4	R	Middle frontal gyrus	45	5.80	131	44	36	20
5	R	Insular cortex	47	3.67	119	30	24	-4
***G. Conjunction morality ∩ ambiguity in task engagement***								
		*No significant results*						
***H. Contrast morality > ambiguity in task engagement***								
1	L	Angular gyrus	39	3.78	371	-50	-60	18
2	R/L	Precuneus	23	3.54	244	4	-60	30
3	R/L	Superior frontal gyrus	9	3.07	237	-10	50	34
4	R	Temporal pole	21	2.26	120	52	8	-26
***I. Contrast ambiguity > morality in task engagement***								
1	R/L	Anterior division of cingulate gyrus	32	4.78	376	4	24	44
2	R	Insular cortex	47	3.94	355	34	20	-4
3	R	Thalamus	-	5.88	315	10	-18	2
4	L	Insular cortex	47	3.46	135	-28	26	2
5	R	Middle frontal gyrus	46	4.97	108	40	38	24
6	R	Inferior frontal gyrus	48	4.34	104	46	18	30
7	L	Angular gyrus	7	2.59	89	-28	-60	42
***J. Conjunction ambiguity ∩ risk in task engagement***								
1	R	Insular cortex	-	4.11	83	32	24	-6
2	R/L	Paracingulate gyrus	24	4.00	82	4	28	36
3	R	Middle frontal gyrus	45	3.90	16	40	36	22
***K. Contrast risk > ambiguity in task engagement***								
1	R	Superior division of lateral occipital cortex	7	3.54	141	28	-60	46
2	L	Middle frontal gyrus	6	3.20	112	-28	0	54
3	R	Inferior frontal sulcus	45	2.62	71	46	34	14
4	L	Precentral gyrus	6	2.14	14	-34	-2	52
***L. Contrast ambiguity > risk in task engagement***								
1	R	Inferior frontal sulcus	44	2.47	81	52	18	30
2	R	Thalamus	-	2.09	71	14	-16	8
3	R/L	Midbrain	-	2.09	69	4	-22	-8
4	R	Insular cortex	47	2.37	58	46	20	-6

Visualization of the results is shown inFigure 4 A–F. Experiment contribution details are given inSupplementary Table 8. Hemisphere, region, Broadman area, Z-value, size in voxels, and MNI coordinates are listed.

*Note:*R, right; L, left; R/L, bilateral.

A meta-analysis on risky decision-making in the*task engagement*category (24 experiments, 576 unique subjects) revealed 5 clusters (seeTable 3BandFig. 4C) in the right superior division of lOCC extending into SPL, the ParaCG, the left MFG extending into the SFG and precentral gyrus, right MFG extending into the FP, and the right aINS extending into the OFC. Experiments contributing to these clusters primarily conducted a gambling or natural risk-taking task (seeFig. 4D) with a similar distribution of experiments that used a low- or a high-level control condition (seeSupplementary Table 8). Contrast analysis (seeTable 3K) revealed that convergence of activations in clusters in the right superior division of lOCC extending into SPL, left MFG extending into SFG, right IFG pars triangularis extending into FP, and left PreCG extending into MFG is more consistently activated in the risk domain as compared with the ambiguity domain.

For ambiguous decision-making in the*task engagement*category (27 experiments, 525 unique subjects), the ALE meta-analysis revealed 7 clusters (seeFig. 4E): medial ParaCG extending into the SFG, the right OFC, the right thalamus, the left aINS, right MFG extending into the FP, right inferior frontal gyrus (IFG) pars opercularis extending into the MFG and the PreCG, the left SPL extending into the superior division of the lOCC. The majority of experiments contributing to these clusters primarily conducted a guessing or probabilistic learning task (seeFig. 4F), with more experiments using a low- than high-level control condition (seeSupplementary Table 8). As compared with the risk domain, the ambiguity domain uniquely engages clusters in the right aINS extending into IFG pars opercularis and MFG, right thalamus, midbrain, and right interior frontal sulcus (IFS) (seeTable 3L).

Conjunction analysis indicated that the risk and ambiguity domains share convergence of activations in the right aINS extending into the OFC and frontal operculum, the medial ParaCG, and the right MFG (seeTable 3J).

#### 3.2.4 *Moral, risky, and ambiguous networks in* choice response

To investigate whether choosing a choice option with a possible higher outcome magnitude and higher uncertainty as compared with choosing an alternative with a lower possible outcome magnitude and higher certainty results in similar or different neural activation patterns in specific decision-making domains, we have also performed ALE meta-analyses in the*choice response*category for the morality and risk domains (we did not find enough experiments to perform this analysis in the ambiguity domain). Functional decoding was also performed for significant results in*choice response*analyses in specific domains (seeSection 3.3.).

In the morality domain (20 experiments; 458 unique subjects), no clusters above the significance threshold level were detected. In the risk domain (31 experiments, 811 unique subjects), we found that risky choices as compared with safe choices are associated with consistent activation in medial SFG extending into ParaCG, and bilateral CN on the right extending into NAcc (seeTable 4BandFig. 4G). Experiments contributing to these clusters primarily conducted a gambling under risk task (seeFig. 4HandSupplementary Table 9). When the risk domain was compared with the morality domain, only the medial cluster in the ParaCG but not the clusters in the bilateral CN was uniquely associated with risky choice (seeTable 4F).

**Table 4.** Shows peak coordinates of clusters of significant convergence revealed by the ALE analyses for each domain in the*choice response*category, as well as results of conjunction and contrast analyses.

Cluster #	Hemisphere	Region	BA	Z-value (uncorrected)	Size (Voxels)	x	y	z
***A. Morality in choice response***								
		*No significant convergence*						
***B. Risk in choice response***								
1	R/L	Paracingulate gyrus	32	4.50	296	-4	38	28
2	R	Caudate	25	5.53	191	10	10	2
3	L	Caudate	25	4.78	189	-10	6	-4
***C. Ambiguity in choice response***								
		*Not enough studies*						
***D. Conjunction morality ∩ risk in choice response***								
		*No significant results*						
***E. Contrast morality > risk in choice response***								
		*No significant results*						
***F. Contrast risk > morality in choice response***								
1	R/L	Paracingulate gyrus	32	3.17	193	-2	36	34

Visualization of the results is shown inFigure 4G–H. Experiment contribution details are given inSupplementary Table 9. Hemisphere, region, Broadman area, Z-value, size in voxels, and MNI coordinates are listed.

*Note:*R, right; L, left; R/L, medial.

### 3.3 Functional decoding

We performed separate meta-analyses in*task engagement (combined)*for the morality, risk, and ambiguity domains, as well as*choice response*for the morality and risk domains. Due to a limited number of experiments in some cases, we were not able to differentiate between experiments using a low-level or a high-level control condition, therefore, we ran meta-analyses on a combined set of experiments with both low-level and high-level control conditions. This analysis approach, on the one hand, ensured that domain-specific consistent functional involvement of particular brain areas was better captured in the meta-analysis but, on the other hand, introduced a possibility of identifying clusters that correspond to domain-general decision-making processing. Therefore, to better differentiate between possible domain-general and domain-specific functions of the meta-analytically determined clusters of convergent activation, we additionally performed a functional decoding analysis of our results in the above-mentioned analyses (functional decoding results for*task engagement (combined)*across domains and*choice response*across domains can be found inSupplementary Fig. 2).

#### 3.3.1 *Functional profiles of the morality, risk, and ambiguity networks in* task engagement

Functional decoding analysis was performed on all meta-analytically identified clusters for the three decision-making domains separately in the*task engagement*category. The results are depicted inFig. 5.

**Fig. 5.**
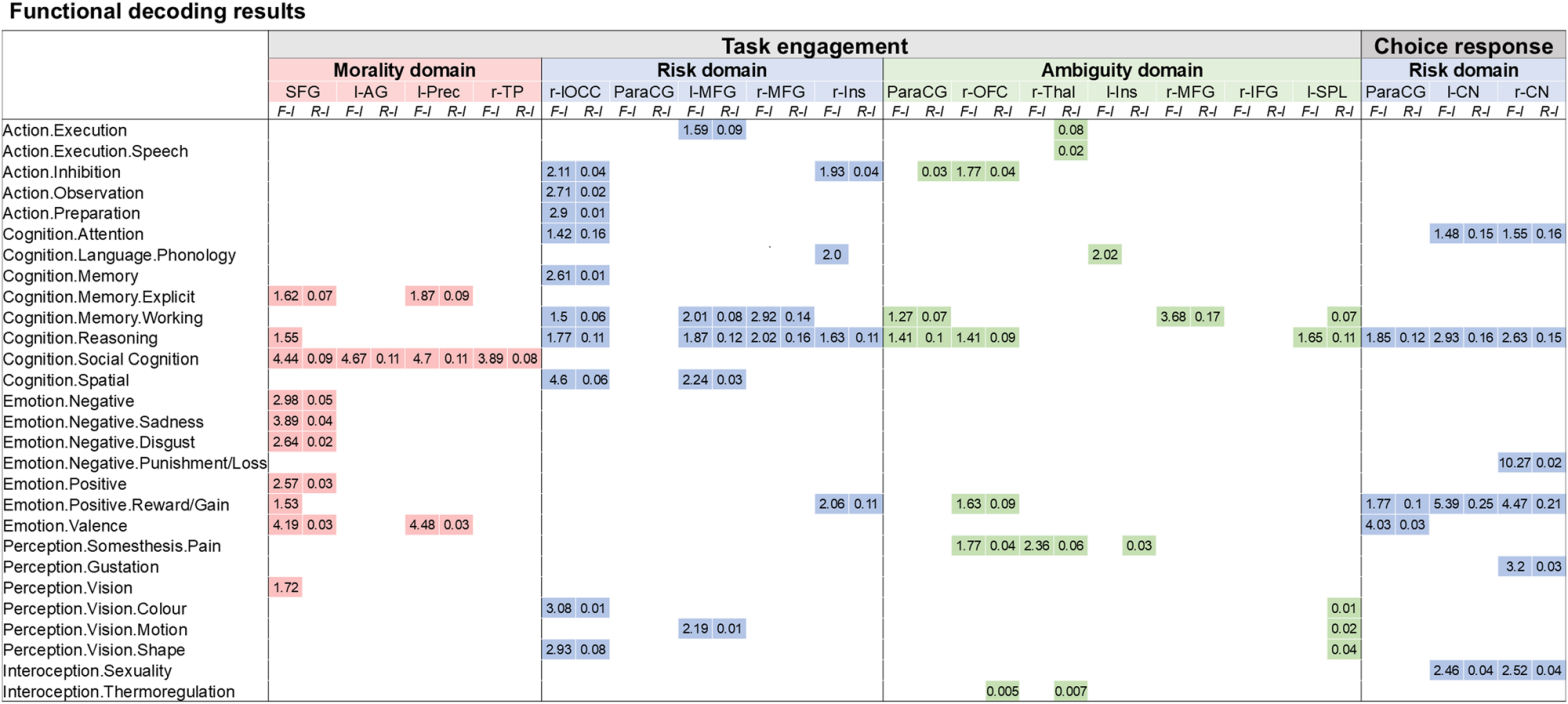
Functional decoding results in forward-inference and reverse-inference analyses, depicted as likelihood ratio and probability assessment, for all identified clusters of convergent activity in the morality, risk, and ambiguity domains in*task engagement*category, as well as the risk domain in*choice response*category. Meaning of abbreviations: m—medial, r—right, l—left, SFG—superior frontal gyrus, AG—angular gyrus, PCun—precuneus, TP—temporal pole, Ins—insula, MFG—medial frontal gyrus, ParaCG—paracingulate gyrus, lOCC—lateral occipital cortex, OFC—orbitofrontal cortex, Thal—thalamus, IFG—inferior frontal gyrus, SPL—superior parietal lobule, CN—caudate nucleus, F-I—forward inference, R-I—reverse inference.

##### 3.3.1.1 Morality domain

Both forward and reverse inference showed that all four clusters—medial SFG, left AG, left precuneus, and the right TP—were functionally associated with social cognition. The cluster in the precuneus and the cluster in the SFG were further associated with explicit memory and emotional processing with specific associations of the SFG with positive and negative emotions. Forward inference also indicated that SFG is associated with cognitive reasoning, processing reward as a positive emotion, and vision.

##### 3.3.1.2 Risk domain

Clusters in the right lOCC, the left and the right MFG, were functionally associated with cognitive reasoning and working memory as shown by both reverse and forward inferences. The right aINS was also associated with reasoning, and additionally with action inhibition. Furthermore, it was the only cluster associated with processing reward as a positive emotion and phonology in language cognition. The right lOCC was further associated with other cognitive functions, such as attention, memory in general, and spatial cognition, as well as perceptual functions such as colour and shape vision. The left MFG was also associated with action execution and visual motion perception. Finally, the cluster in the ParaCG had no functional associations in both forward and reverse inferences.

##### 3.3.1.3 Ambiguity domain

Clusters in the ParaCG, right OFC, and left SPL were associated with cognitive reasoning by both forward and reverse inference. The ParaCG, the right MFG, and the left SPL were linked to working memory (the latter by reverse inference only). The ParaCG and the right OFC were associated with action inhibition (the former by reverse inference only), while reverse inference showed association with action execution and execution of speech for the cluster in the right thalamus. The cluster in the left aINS was associated with phonology in language cognition by forward inference. The cluster in the right OFC (extending to aINS and overlapping with the cluster in the right aINS found in risk domain) was associated with processing reward as positive emotion. The clusters in the right OFC, the right thalamus, and the left aINS were functionally linked to pain perception (the latter by reverse inference only). The left SPL was also associated with vision functions, such as colour, motion, and shape perception. Finally, reverse inference indicated thermoregulation functions for clusters in the right OFC and the right thalamus.

#### 3.3.2 *Functional profile of regions of the risk network in* choice response

##### 3.3.2.1 Morality domain

No statistically significant clusters were identified in the meta-analysis, therefore, functional decoding was not performed.

##### 3.3.2.2 Risk domain

In the choice response analysis approach, all three meta-analytically identified clusters—ParaCG as well as bilateral CN—were functionally associated with reward processing and reasoning. The ParaCG was also associated with emotion processing. The two caudate nuclei were additionally associated with cognitive attention and sexuality interoception, while the right CN was associated with punishment processing and gustation (Fig. 5).

## 4 Discussion

In this meta-analysis, we sought to investigate the similarities and differences between neural correlates of moral, risky, and ambiguous decision-making domains in the categories of*task engagement*as well as*choice response*, that is, the two categories representing different condition comparison strategies in the field of decision-making. We found that both*task engagement*and*choice response*consistently engaged the salience network, although possibly in different functional roles, while only*choice response*captured consistent activation across experiments in the subcortical brain areas related to reward processing. When assessing morality, risk, and ambiguity domains separately in the*task engagement*category, we found that only the morality domain engaged the social cognition network. The risk and ambiguity domains shared overlapping convergence of activations in the salience network. The risk domain engaged the frontoparietal attention network to a greater extent than ambiguity, while the ambiguity domain additionally employed subcortical brain areas belonging to the salience network. Finally, we also observed that different neural mechanisms in the medial prefrontal cortex (mPFC) supported reward salience processing in the morality, risk, and ambiguity domains.

### 4.1 Domain-general neural circuit of decision-making

In this meta-analysis, we were interested if domain-general cognitive processes supporting decision-making would be based on anatomically shared neural circuits in moral, risky, and ambiguous decision-making. We have postulated that the condition comparison of experimental task with a low-level control condition (e.g., resting-state scan, guided motorvisual task) would capture consistent involvement of a large set of brain areas, involved in domain-general cognitive processes, such as objective value computation, cognitive control, attention allocation, and conflict resolution.

To investigate this, we have meta-analytically assessed spatial convergence of activations of all experiments that used a low-level control condition from the three domains—morality, risk, and ambiguity—together (analyses of these experiments for each domain separately were not possible due to insufficient number of experiments in each domain). Contrary to our general expectation that a broad neural circuit underlies domain-general processing of decision-making, the meta-analytical assessment resulted in only three clusters of consistent activation: a large medial cluster in the dorsal anterior cingulate cortex (dACC) and a cluster in the right supramarginal gyrus (SMG, located in the inferior parietal cortex,Wild et al., 2017), both associated with a wide range of domain-general cognitive functions, such as attention allocation, salience, and cognitive control (Niendam et al., 2012;Seeley, 2019), and a cluster in the right orbitofrontal cortex (OFC), commonly associated with conflict resolution and value signal integration (Padoa-Schioppa & Conen, 2017). All these clusters were primarily driven by experiments from the risk and ambiguity domains. Therefore, our analysis did not provide evidence that, first, the use of a low-level control condition in decision-making tasks consistently allows one to capture a domain-general decision-making circuit across various heterogeneous decision-making tasks. Second, our results also did not suggest that the morality domain similarly engages domain-general neural processing of objective value computation, cognitive control, attention allocation, and conflict resolution. However, we do not suggest that these results undermine the existence of a domain-general decision-making circuit and its involvement in various value-based decision-making domains.

One possible reason why we did not capture a large set of consistently activated clusters of activation throughout the experiments with a low-level control condition is the heterogeneity in methodological choices for contrast analysis. There is variability across the experiments included in our meta-analysis in how minimal the low-level control condition was (e.g., resting-state baseline allows to capture visual processing, while a guided motorvisual task would not), how conservative or liberal the correction for multiple comparisons was, and, of course, how the imaging results were modulated by specific task effects.

In our analysis, experiments with a low-level control condition from the morality domain contributed only a little to the identified clusters of convergent activation. One possible explanation is that during our literature search, we were only able to identify seven experiments using a low-level control condition from the morality domain that fit our inclusion and exclusion criteria, while in the risk and ambiguity domains, we obtained 14 and 13 experiments, respectively. Imbalance in the number of experiments in the analysis, in this case, could have led to a low contribution to meta-analytical results from the morality domain.

Several studies provide evidence that the salience network is interconnected with the social cognition network during moral decision-making (Chiong et al., 2013;Sevinc et al., 2017). Functionally this network might be responsible for early moral cue detection (Sevinc et al., 2017). However, the salience network might also play a larger role in moral decision-making: in a study investigating individuals suffering from behavioural variant frontotemporal dementia,Chiong et al. (2013)have demonstrated that the salience network (via the aINS) causally modulates the strength of functional coactivation of the social cognition network during moral decision-making. To conclude, more research is needed to investigate how the social cognition network, involved in the morality domain, interacts with the domain-general decision-making circuit during moral decision-making. Direct comparison with findings from other value-based decision-making domains could then provide clues about similarities and differences in how the neural processing in the domain-general decision-making circuit is involved in different domains.

### 4.2 Domain-specific neural circuits underlying morality, risk, and ambiguity

We were further interested if different value-based decision-making domains share any consistent activation associated with domain-specific processing. We have investigated this research question by using two different approaches. First, we have combined all experiments across the three domains—morality, risk, and ambiguity—in the*task engagement*category that compared an experimental decision-making task with a high-level control condition. Second, we divided all experiments in the*task engagement*category with both low-level and high-level control conditions according to the specific domain—morality, risk, or ambiguity—and performed separate meta-analyses on these three groups of experiments.

As hypothesized, both meta-analysis approaches revealed that only the morality domain engages the core nodes of the social cognition network: medially located clusters in the dmPFC and precuneus, left temporoparietal junction (TPJ), and the right temporal pole (TP). Our results (seeTable 3A) are largely comparable with previous meta-analyses on morality (seeSupplementary Fig. 8), however, some differences emerged due to several factors: (1) we have included a significant number of new studies published since the publication of the previous meta-analyses (n = 22 new studies in the morality domain compared with the most recent meta-analysis byEres et al., 2018) as well as some unpublished data, and (2) we did not differentiate between task instructions in moral dilemmas or restrict inclusion criteria to only one type of task instruction (as done byEres et al., 2018;Garrigan et al., 2016). Both previous meta-analyses investigated consistent activation across studies of moral cognition and demonstrated that vmPFC and right TPJ depended on specific task instructions and task content (e.g., proximity to the victim). As we did not separate studies by task instructions and included more studies, this may explain the lack of convergence of activity in comparison with previous meta-analyses (Eres et al., 2018;Garrigan et al., 2016).

For the risk and ambiguity domains, we have found that domain-specific cue processing in the*task engagement*category recruits brain regions of the salience network and parts of the frontoparietal attention network (seeTable 3B, C). Our conjunction analysis between the risk and ambiguity domains indicated that domain-specific cue processing in the two domains shares spatial overlap in the core nodes of the salience network, the right aINS and dACC, and in the right medial frontal gyrus (MFG), which belongs to the frontoparietal attention network. The conjunction results between the risk and ambiguity domains, indicating shared consistent activation in the core nodes of the salience network (seeTable 3J), reinforce the association of the salience network with uncertainty processing (Esber & Haselgrove, 2011).

Both risky and ambiguous decision-making tasks involve uncertainty, yet they do so to a different degree. This might be reflected in the extent to which the salience network is involved in processing uncertainty during these tasks. Our contrast analyses between the risk and ambiguity domains revealed that the thalamus and midbrain structures are consistently involved in ambiguous but not in risky decision-making. It has been shown that the thalamus and midbrain participate in salience processing (for a review, seeZhou et al., 2021) via the cortico-striato-thalamo-cortical loop of the salience network, which is responsible for cognitive control (Peters et al., 2016).

In our results, the risk domain was associated more with a part of the frontoparietal network (Uddin, 2015), that is, a cluster in the middle frontal gyrus (MFG) and a cluster in the lateral occipital cortex (lOCC) extending to the intraparietal sulcus (IPS), as compared with the ambiguity domain (seeTable 3K). As the frontoparietal network is associated with individual self-control (Baltruschat et al., 2020;Gentili et al., 2020;Lee & Telzer, 2016), and loss evaluation (Brevers et al., 2017;Dong et al., 2015;Losecaat Vermeer et al., 2014;Xue et al., 2011;Zeng et al., 2013) in the risk domain, its engagement in the risk but not ambiguity domain might represent different task effects for cue processing. Regarding risk as compared with ambiguity, risky decision-making tasks present reward probability to the participants, allowing them to engage in a more detailed yet more cognitively demanding cost–benefit analysis than ambiguity tasks, which provide no or partial information on the reward probabilities. Differential processing of risk and ambiguity was also found in inferior frontal sulcus (IFS), seeSupplementary Discussionfor more details.

Compared with other meta-analyses of consistent activation in the risk and ambiguity domains (e.g.,Poudel et al., 2020;Wu et al., 2021), our meta-analysis yielded different results in several respects (seeSupplementary Fig. 8): (1) By separating the analysis categories of task engagement and choice response, we were able to show that striatal activation is specific to the selection of risky or ambiguous choice options, but not to general decision-making in risk or ambiguity contexts (seeSection 4.3.). It also allowed us to identify smaller but important clusters of consistent activation, such as the IFS in the risk and ambiguity domains and the thalamus in the ambiguity domain, that were not captured when the convergence of activations was assessed without separating the different analysis categories. (2) Some differences between the results may be due to differences in the inclusion and exclusion criteria for study samples (our meta-analysis only included data from healthy adult samples, whereasPoudel et al. (2020)also included clinical samples) and the exclusion of imaging results obtained by parametrically modulating risk and ambiguity levels (e.g., both meta-analyses byPoudel et al., 2020;Wu et al., 2021).

We have argued that the morality domain might share consistent activation with the risk and ambiguity domains in the brain areas that process uncertainty, for example, aINS. It has been shown that in experimental moral dilemmas, participants intrinsically assign probabilistic uncertainty to the choice options (Shou & Song, 2017;Shou et al., 2020). However, we did not find any convergent activity in the aINS between the domains in*task engagement*category, and only low contribution of moral tasks to aINS in combined*task engagement*category across the domains. It might be the case that processing of probabilistic uncertainty in the morality domain is nevertheless significantly weaker than in the risk or ambiguity domains, and its neural correlates cannot be captured with traditional condition comparison methods. Future studies assessing neural underpinnings of uncertainty in the morality domain should directly manipulate levels of probabilistic uncertainty of the outcome choices in moral dilemmas and investigate whether this type of uncertainty is processed in the salience network.

Interestingly, our domain-specific meta-analyses for each domain in the*task engagement*category resulted in clusters of convergent activation in mPFC, indicating possible value and reward cue processing, just in different locations for each domain. In the morality domain, we observed consistent activation across experiments in a medial cluster in the dmPFC (seeFig. 6). This region has been associated with subjective value and reward processing, independently of the task domain and also independently of whether the decision affected oneself or the other (Piva et al., 2019). In line with this literature, our functional decoding analysis associated this cluster with social cognition, emotion recognition, reasoning, and reward processing. Adjacent to the dmPFC cluster in the morality domain, clusters in the dACC for risk and ambiguity were discovered in*task engagement*and a dACC cluster for risk in*choice response*(seeFig. 6). Our functional decoding results suggest that the dACC is associated with both salience and reward-related processing, possibly representing reward salience as well. This would fit with the previous findings, indicating that this area has temporal properties, allowing the subject to compare past and recent rewards and switch choice response if necessary (Wittmann et al., 2016), and that it is functionally connected to other reward-related brain areas, such as dorsal and ventral striata (for a review, seeHaber, 2016).

**Fig. 6.**
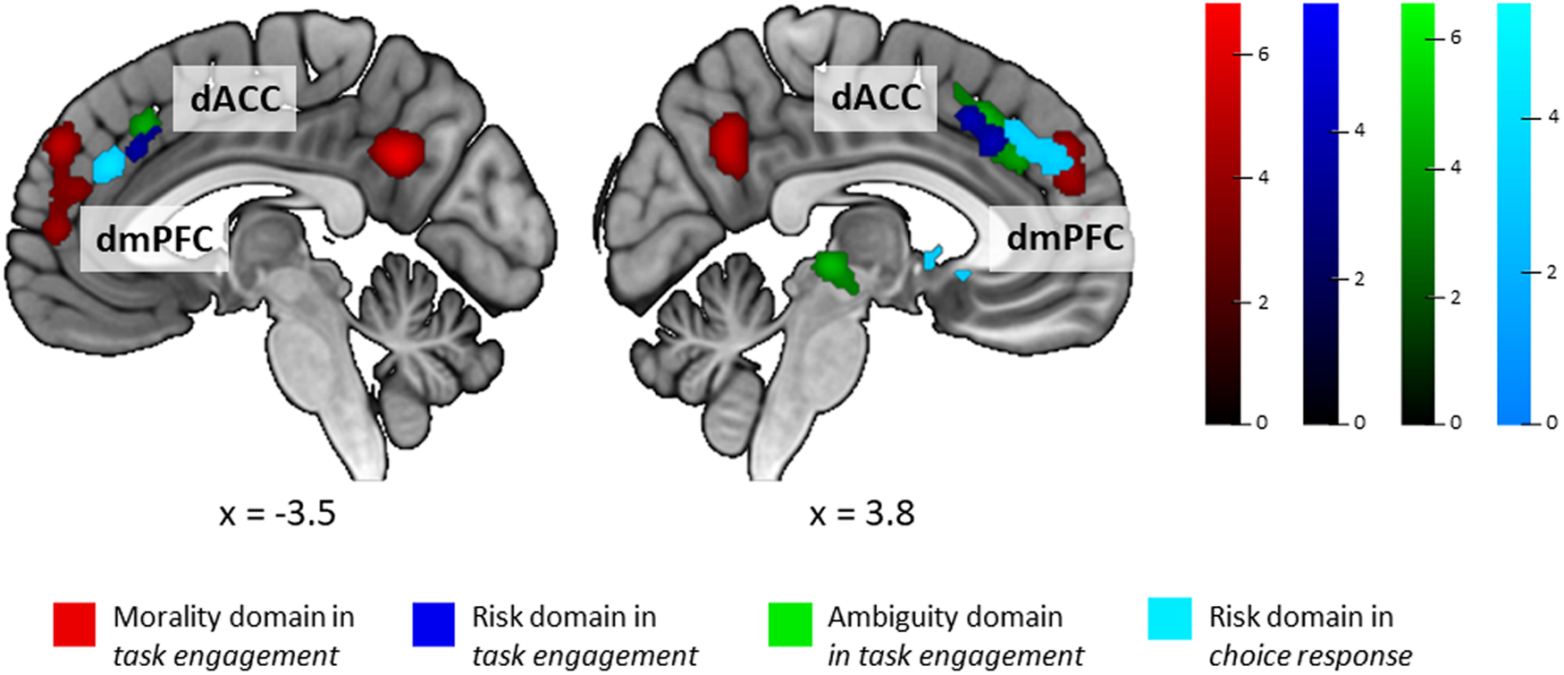
Differential involvement of mPFC in morality, risk, and ambiguity domains. Overlays depict possible functional parcellation of dmPFC and dACC regions in morality, risk, and ambiguity domains in task engagement and choice response categories.

Taken together, our findings could indicate that reward salience processing in higher cortical areas is functionally differentiated in the mPFC depending on the contextual domain, for example, morality, risk, or ambiguity, in which the reward signals are generated. We suggest that experiments using computational models where social and non-social rewards are manipulated focusing on these separate regions of interest could be useful for further investigations of the involvement of these brain areas in domain-specific reward processing.

### 4.3 Choice-specific neural underpinnings in morality, risk, and ambiguity

In this meta-analytical investigation, we separated domain-general, domain-specific, and choice-specific neural correlates by considering different condition comparison strategies applied in decision-making experiments. Regarding the choice-specific convergence of activations, that is, the*choice response*category, we were interested if choices with higher outcome magnitude and higher uncertainty (either probabilistic or social) as compared with “safe” choices (lower outcome magnitude but higher certainty) in the morality, risk, and ambiguity domains result in similar or different pattern of consistent activations. To answer this question, we performed several meta-analyses. First, we combined all experiments across the three domains using the*choice response*condition comparison strategy and estimated contributions of each domain to the meta-analytical results. Second, we ran separate meta-analyses on experiments in the*choice response*category from the morality and risk domains (not enough experiments were available for the ambiguity domain).

Several main findings could be concluded from these analyses and their comparison with the*task engagement*category. First, consistent involvement of reward-related subcortical brain areas (the bilateral caudate nuclei (CN) extending into nucleus accumbens (NAcc)) was captured only when choice-specific activation in*choice response*but not when consistent domain-general or domain-specific activation in the*task engagement*category was investigated. Second, we found some evidence that the higher outcome magnitude of the selected choice might be represented by higher consistent activation in the same brain areas in the morality and risk domains, as hypothesized. However, we did not find enough evidence in previous literature to hypothesize about differences in such choices in different domains. Unfortunately, we were also unable to provide this evidence, as our*choice response*analyses for specific domains only provided statistically significant findings in the risk domain. Finally, we found that the salience network was involved in both domain-specific and choice-specific analyses. We discuss these findings and their implications in more detail below.

When analysing the main effects of analysis approaches, we discovered that consistent activation across experiments in the bilateral CN/NAcc, a part of the left aINS extending into the OFC, and the right superior frontal gyrus (SFG) was not captured by*task engagement*but by*choice response*category only, as predicted by our hypothesis for the*choice response*category (seeTable 2). Similarly, convergence in the bilateral CN/NAcc was found in the risk domain for*choice response*but not for*task engagement*. These findings suggest that choices of higher possible outcome magnitude and higher outcome uncertainty consistently are associated with neural processing in subcortical reward-related brain areas, most likely representing the difference in anticipation of outcome magnitude (Zhang et al., 2017). Our findings are in line with previous findings which show that striatum encodes both anticipated reward magnitude and reward probability and increases its activation when reward magnitude or uncertainty increases (Preuschoff et al., 2006;Yacubian et al., 2007). Further, the spatial locations of our identified cortical brain areas, including the aINS, closely resemble the findings of a previous searchlight study, which found that neural activation change patterns in these brain areas can predict risky choices but not safe choices (Helfinstein et al., 2014), possibly indicating a specific neural circuit responding to higher uncertainty with higher functional activation. Furthermore, resting-state activity in these brain areas (NAcc, OFC, lateral PFC, and dACC) positively correlated with expected benefit from a risky choice (Cox et al., 2010). This confirms that for one to choose an option with higher possible outcome magnitude but also higher uncertainty, a widely distributed network of brain areas with diverse functional associations, such as reward, salience detection, attention, working memory, and motor action preparation, is required.

We also found that choices of higher possible outcome magnitude and higher social uncertainty in the morality domain contributed to the bilateral CN/NAcc clusters to a meaningful extent as compared with the risk domain (seeFig. 2FandSupplementary Table 7). The contributing experiments from the morality domain were investigating altruistic and prosocial behaviour and in equal proportion the specific choices by the participants to donate more as compared with the opposite choice or keep more to themselves as compared with the opposite choice. Since the choices affected different receivers but still had the higher possible outcome magnitude in common, we cautiously suggest that consistent involvement of the bilateral CN/NAcc clusters in these choices corresponds to the higher outcome magnitude similarly as in the risk domain. We also found that experiments from the morality domain meaningfully contributed to the left aINS cluster. In this case, most contributing experiments contrasted more socially uncertain choices with socially preferrable choices, possibly indicating that the left aINS could be a target region of interest to further investigate neural correlates of social uncertainty processing—similarly as uncertainty processing in the risk domain. However, as our analysis of the*choice response*category in the morality domain separately did not yield significant results and we were unable to run conjunction and contrast analyses between the domains, interpretation of these results should be further tested in future studies, for example, by modulating possible outcome magnitude and level of social uncertainty in a parametric manner.

We have further perfomed a conjunction analysis between the*choice response*and*task engagement*categories across all three decision-making domains to investigate whether domain-specific and choice-specific invovement of uncertainty processing are represented in the same or distinct brain areas. This analysis demonstrated consistent involvement of the bilateral aINS and ParaCG cluster corresponding to dACC in both*task engagement*and*choice response*categories (seeTable 2CandFig. 7). As mentioned before, these brain areas constitute the core of the salience network (Seeley, 2019). Convergence of activity in these brain areas was to a large extent resulting from contributions of risk and ambiguity experiments in both*choice response*and*task engagement*categories, indicating that this network is consistently involved in uncertainty processing in the non-social domains, expressed as both variance in outcome probability and missing information on outcome probability.

**Fig. 7.**
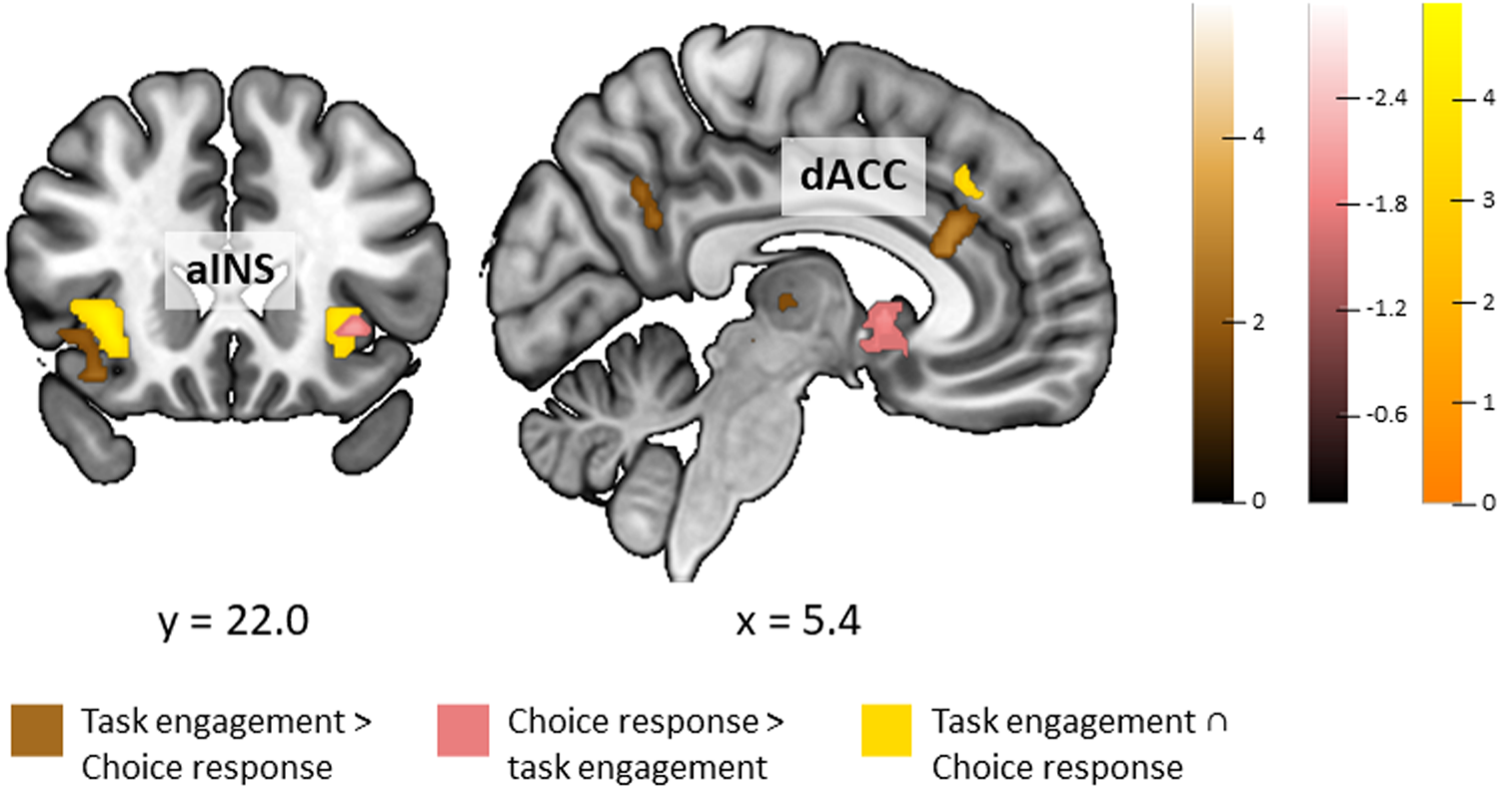
Involvement of the salience network in*task engagement (combined)*and*choice response.*Overlays depict differential involvement of core brain areas of the salience network, that is, aINS and dACC, in*task engagement*and*choice response*categories across all decision-making domains. Gold clusters represent task engagement > choice response contrast; pink clusters represent choice response > task engagement contrast; yellow clusters represent conjunction between task engagement and choice response.

Importantly, our results endorse the dual-process model of salience (Esber & Haselgrove, 2011;Pearce & Mackintosh, 2010). This model postulates that two separate salience components are computed in the brain: one for stimulus selection where predictive cues produce a stronger saliency response than irrelevant cues, and another one for learning where a stronger salience signal is computed for more uncertain cues. In*task engagement*, it is likely that the consistent activation of the salience network as compared with a control task is involved in predictive cue computation, when participants engage with the task and detect specific stimulus cues (George et al., 2010). On the other hand, our results in*choice response*provide evidence that uncertain cues in the environment are more salient than the certain ones (Esber & Haselgrove, 2011), as we were looking for convergence of activations associated with an uncertain choice (with known partial probabilities for outcome in risk and unknown probabilities for outcome in ambiguity) as compared with a safe choice (known outcome).

As our conjunction and contrast analyses between*task engagement (combined), task engagement (when only experiments with low-level control condition are considered), task engagement (when only experiments with high-level control condition are considered)*, and*choice*response across domains indicate, a particular parcellation of the aINS could represent differences in how salience processing is involved in domain-specific and choice-specific manner. This might have clinical relevance as it has been previously demonstrated that the left aINS of sensation-seeking individuals showed increased activation when making risk appraisal as compared with that of individuals who score low on sensation seeking (Kruschwitz et al., 2012). Moreover, a recent study investigating twins discovered genetic influence on risk-taking behaviour and corresponding activation in the left aINS and the right striatum (Rao et al., 2018). This indicates that there might be a specific part of the left aINS, which is involved when a more uncertain but possibly more rewarding option is chosen, and its activity can be further modulated by individual differences.

A potential functional lateralization effect has also emerged from our results: the right aINS/OFC clusters in*task engagement*in the risk and ambiguity domains (seeFig. 3C) as well as*task engagement (combined)*and*choice response*across domains (seeSupplementary Fig. 3) were functionally associated with positive emotional processing of reward gain, supporting previous literature revealing the role of the aINS in regulating and integrating reward-relevant information (reward salience) by receiving valence and preference inputs from the amygdala and PFC (Kim et al., 2011;Naqvi & Bechara, 2009) and sending outputs to the striatum and other brain regions that are involved in reward-related computations (Haber, 2016).

Taken together, our results indicated that in the*task engagement*category when the experimental condition entails all types of choices made by participants and is then compared with a control condition as compared with*choice response*category, the subtle differences in neural correlates of anticipated outcome magnitude, attached specifically to one type of choice, are not consistently captured because they are annulled by inclusion of the opposite choices (high vs. low outcome magnitude) in the same analysis. Similarly, if the different condition comparison approaches are not assessed separately, the dual involvement of the salience network in processing uncertainty might be overlooked. This is especially important to keep in mind when meta-analytical assessments are performed: decision-making experiments using contrasts from*task engagement*and*choice response*categories should not be combined into one analysis, as they obscure functional interpretation of the identified anatomical convergence of activations.

## 5 Limitations And Future Directions

In this meta-analysis, the included neuroimaging experiments in the domains of morality, risk, and ambiguity for*task engagement*differ by how often a high- or low-level control condition was used. In the morality domain, the most common analytical approach is to use a high-level control condition, which involves non-moral decision-making processes; while in risk and ambiguity, both high- and low-level control conditions are implemented more equally, although also to a lesser extent than direct comparisons of conditions capturing neural correlates of the actual choices. Unfortunately, at the moment, there are an insufficient number of experiments that would allow for meta-analytical comparisons according to the high- or low-level of control conditions in the different domains in*task engagement*. With enough experiments, this would allow us to distinguish the particular task effects on the domain-general decision-making network, domain-specific decision-making network, as well as the network specifically processing the situational cues of interest. Differentiating the extent of the control condition could also benefit research into mental disorders affecting decision-making. It would further provide fundamental knowledge on decision-making mechanisms and aid in the advancement of personalized healthcare.

It is also important to note that our results in the morality domain are primarily driven by tasks which implemented sacrificial harm dilemmas as stimuli. In our meta-analysis, we aimed to balance the negatively and positively valenced outcomes of moral decision-making and combined neuroimaging results into one category, expecting that the results from positively valenced outcomes would contribute to a better understanding on shared processing of moral cues, as based on previous literature (Hare et al., 2010;Hu et al., 2021;Van Overwalle, 2009;Wu & Hong, 2022). However, in the*task engagement*category, we were able to include only two studies investigating prosocial behaviour that used a*task engagement*type of condition comparison strategy. While these studies have contributed to our meta-analytical results, a more balanced meta-analytical investigation is required. Comparably, altruistic and prosocial decision-making as well as risky decision-making are investigated in terms of comparing neural correlates of actual choices rather than experimental task versus control task. Future studies could consider applying this condition comparison strategy to their studies in addition to the comparison of actual choices. Moreover, individual investigations into differences between the neural correlates of negatively and positively valenced moral decision-making could provide evidence for common neural mechanisms underlying moral behaviour despite its outcome valence.

Furthermore, no significant results were found in the morality domain for*choice response*. In this category, we were able to include experiments from sacrificial harm dilemmas and prosocial decision-making to a similar extent, and some deception experiments in addition. It seems that the heterogeneity of the experiments underlies the lack of convergence of activations in this analysis. When enough studies investigating actual choices in sacrificial harm dilemmas and prosocial decision-making are available, we suggest analysing these experiments based on the respective tasks separately. Unfortunately, due to a low number of experiments, this was not implementable at the moment.

We aimed to compare the three decision-making domains in the*choice response*category, but due to a small number of studies we were not able to run the analysis for the ambiguity domain. As compared with risky choices, ambiguous choice options are more ecologically valid and more representative of a wider variety of choices ordinary people encounter in their daily life. Presence of ambiguity in choice options might differentially affect individuals with mental disorders that alter decision-making, based on disturbed neural processing in either domain-general decision-making network or processing of ambiguity cues. Differences between neural correlates of ambiguous and risky choices could further inform the researchers about possible neural targets for treatment interventions.

Finally, our results in the morality, risk, and ambiguity domains somewhat diverge from the previous meta-analyses, which also considered morality, risk, or ambiguity (Bzdok et al., 2012;Eres et al., 2018;Garrigan et al., 2016;Poudel et al., 2020;Wu et al., 2021). While most of the clusters of convergent activation in our meta-analysis replicate previous findings, some differences between our and other meta-analyses arise because of different experiment inclusion strategies. For example, unlike other meta-analyses, we have included only explicit tasks in the morality domain. Here we also included sacrificial harm dilemma tasks with a few variations in exact instruction and did not differentiate between them. In risk and ambiguity domains, we have excluded experiments reporting results of parametric modulation of risk and ambiguity as well as contrasts comparing risk and ambiguity conditions directly to each other. Finally, across domains, we separated experiments according to their condition comparison strategy into*task engagement*and*choice response*categories. These methodological as well as theoretical differences in our meta-analysis resulted in a somewhat different set of clusters of convergence and allowed us to pick up a more nuanced neural representation of processes involved in decision-making, without contradicting the findings of other meta-analyses.

## 6 Conclusion

Our coordinate-based ALE meta-analysis compared the neural correlates of the morality, risk, and ambiguity domains and separated*task engagement*and*choice response*analysis strategies. We found that moral cues are processed in a multi-modal social cognition network, while the risk and ambiguity domains require engagement of the salience and the frontoparietal attention networks. Besides replicating and extending previous meta-analytical results, our meta-analysis demonstrated that in brain areas, such as aINS and mPFC, functional parcellation of these regions and lateralization effects exist depending on the decision-making domain. Importantly, we also demonstrated that it is necessary to separate*task engagement*and*choice response*categories when analysed and compared meta-analytically—as they capture different neural mechanisms underlying general decision-making as compared with specific choices. Separation of these condition comparison strategies resulted in support for the dual-process theory of salience. Our findings, therefore, highlight the importance of considering the specific analysis approaches in the field of decision-making, not only for meta-analyses but in individual studies as well. This approach of differentiating*task engagement*from*choice response*might allow the researchers investigating brain functions related to outcome magnitude and social or non-social uncertainty processing to better define how the neural correlates of these functions are modulated. This, in turn, might have clinical relevance to individualized medicine as individual behavioural differences might emerge due to functional deficits in either domain-general, domain-specific, or choice-specific neural correlates. Applying different condition comparison strategies and comparing clinical populations to each other or to healthy cohorts might allow researchers to pinpoint target brain areas for the use of neurostimulation or, in the future, targeted medication.

## Supplementary Material

Supplementary Material

## Data Availability

The data that support the findings of this study are available athttps://osf.io/j7df6/.

## Footnotes

* However, in many instances, the terminology used in this domain has been misleading: the terms “moral decision-making” or “moral judgment” have been extensively used even in cases, where a high-level control task was contrasted to the experimental task, implying that the remaining BOLD signal may represent all general cognitive functions involved in decision-making and not the very specific ones related to the domain-specific cue.
