# Supplementary material for: Distinct neural networks of task engagement and choice response in moral, risky, and ambiguous decision-making: An ALE meta-analysis
Source: Imaging Neurosci (Camb). 2024 Aug 30;2:imag-2-00277. doi: 10.1162/imag_a_00277 (PMC12290865; doi:10.1162/imag_a_00277)
Supplement: Supplementary Material [file imag_a_00277-supp.pdf]

Supplementary material for "Distinct neural networks of task engagement and response choice in moral, risky and ambiguous decision-making: an ALE meta-analysis"

Aiste Ambrase, Veronika I. Müller, Julia A. Camilleri, Hong Yu Wong, Birgit Derntl

Content:

- Supplementary Methods: Selection criteria for the tasks
- Supplementary Discussion: Functional parcellation of Inferior frontal sulcus in risk and ambiguity domains
- Supplementary Figure 1. IFS in risk and ambiguity domains.
- Supplementary Table 1. Checklist for neuroimaging meta-analyses (Müller et al., 2018)
- Supplementary Table 2. Included studies
- Supplementary Table 3. Combined risk and ambiguity domains – Uncertainty domain
- Supplementary Table 4. Activation clusters across all experiments
- Supplementary Table 5. Activation clusters across all experiments for each domain
- Supplementary Table 6. Task contributions in *task engagement* category across domains
- Supplementary Table 7. Task contributions in *choice response* category across domains
- Supplementary Table 8. Task contributions in *task engagement* category for morality, risk, and ambiguity domains
- Supplementary Table 9. Task contributions in *choice response* category for risk domain
- Supplementary Figure 2. Task engagement category across domains (combined experiments with low- and high-level control conditions) and domain contributions
- Supplementary Figure 3. Functional decoding results for combined task engagement and choice response categories across the domains
- Supplementary Methods and Results: Resting-state functional connectivity analysis between meta-analytically identified clusters of consistent activation in morality, risk, and ambiguity domains
- Supplementary Table 10. Resting state functional connectivity, conjunction of moral, risky, and ambiguous domains in *task engagement* category
- Supplementary Figure 4. Resting-state functional connectivity analysis between meta-analytically identified clusters of consistent activation in morality, risk, and ambiguity domains
- Supplementary Figure 5. Resting-state functional connectivity results for combined nodes in morality domain for *task engagement*
- Supplementary Figure 6. Resting-state functional connectivity results for combined nodes in risk domain for *task engagement*
- Supplementary Figure 7. Resting-state functional connectivity results for combined nodes in ambiguity domain for *task engagement*
- Supplementary Figure 8. Comparison of our results with other recent meta-analyses
- References

Supplementary Methods: Selection criteria for the tasks

Morality domain. In the domain of moral decision-making, experimental tasks using harm- or help-based moral dilemmas (including tasks either posing a question "What would you do?" or "How appropriate is to…?") representing negatively and positively valanced decisions, deception opportunities, altruistic or decision-making where the participants' decision would affect a third party were included. This is based on Kohlberg's theory which postulates that moral reasoning predicts moral behaviour (Fang et al., 2017). It has been demonstrated that behaviourally experiment participants respond to moral dilemmas in a similar manner when proposed action in the dilemma is formulated as a binary choice ("Yes/No") or as acceptability rating on a scale (McNair et al., 2019).

In the experimental tasks, the participants were required to act as a decision-maker and not a decision-receiver. For example, in altruistic tasks such as the Ultimatum game or one-shot investment games as well as strategic decision-making tasks such as the Dictator game or Trustee game, participants should have been in the position of the proposer, investor, dictator or trustee; studies in which experimental tasks put the participants in the role of the offer receiver were excluded. Punishment tasks where participants acted as a judge of a violation and ascribed punishment were also excluded. Although these tasks also involve moral deliberation, we were interested in the first-person perspective when deciding or judging the moral permissibility of proposed actions.

Risk domain. In the domain of risky decision-making, gambling tasks such as Risky Gains Task, Wheel of Fortune, Game of Dice Task, Cups Task, Cambridge Gambling Task, various investment, and similar tasks as well as Iowa Gambling Task, Chicken Game, and Balloon Analog Risk Task were included. The latter three tasks constitute what is called "naturalistic risk-taking" and substantially differ from the neuroeconomic tasks by the fact that the reward probabilities in the naturalistic risk-taking tasks are generally unknown (Congdon et al., 2013). Risk-taking behaviour is captured well by naturalistic risk-taking tasks in healthy and clinical populations and correlate with other self-report risk-taking measures (for a review, see Schonberg et al. (2011)). In these tasks, even though the probabilities of the outcome are generally unknown, similarly as in probabilistic learning paradigms, individuals learn the abstract probabilities (as in the Iowa Gambling Task) or are instructed the probability of a loss increases with each of their choice to gamble further in a continuous gamble (as in the Balloon Analog Risk Task). In one study using the BART, explosion probabilities were provided but the task engagement still correlated with self-reported risk-taking (Pleskac, 2008), indicating that limited knowledge of probabilities does not affect risk-taking behaviour. Therefore, as outcome probabilities in these tasks can be implicitly deducted, studies using these paradigms were included in the risk domain as opposed to the ambiguity domain, even though a certain degree of ambiguity is conceptually involved in these paradigms (Schonberg et al, 2011).

Ambiguity domain. In domain of ambiguous decision-making, tasks exploring guessing, probabilistic learning, exploration/exploitation (Two-, Four- or Six-armed Bandit Task), gambling with limited or omitted information on outcome probabilities, as well as complete ignorance about outcome probabilities, and 50/50 chance of the desired outcome were included. Tasks using relying on perceptual (visual) ambiguity were excluded. While most of the studies in the domain used gambling or similar set-ups, several studies employed driving, aviation, or weather prediction stimuli for a more naturalistic depiction of decision ambiguity.

Studies which used "50/50 chance" paradigms have been included in the ambiguous decision-making even if the study authors termed the task as risk-taking. Structurally "50/50 chance" tasks are similar to guessing tasks with two options to choose from. The participant in these tasks is at an indifference point regarding the two options as they are equal in both

objective and subjective values. Furthermore, Huettel (2006) in his influential study on different types of uncertainty separated two terms: reward risk and behavioural risk, with the latter meaning uncertainty about the optimal action in the decision situation. Behavioural uncertainty might stem from the situation where equal probabilities of the choice options provide no further information on the optimal action (Lauriola et al., 2007) or from the situation where information about the probabilities of reward is not fully disclosed (Bach et al, 2009) or fully missing (e.g. decision under ignorance (Pushkarskaya et al., 2010)). In the later literature, the term “behavioural risk” was called “ambiguity” and the meta-analyses by Poudel et al. (2020) and Iyer et al. (2021) also categorized the study by Huettel (2006) as ambiguous. An early meta-analysis by Krain et al (2006) categorized a 50% chance condition in the study by Paulus et al (2003) as ambiguity.

Supplementary Discussion: Functional parcellation of Inferior frontal sulcus in risk and ambiguity domains

Our meta- as well as contrast analyses between the risk and ambiguity domains revealed different parts of the inferior frontal sulcus (IFS) for the two domains respectively. The cluster of convergent activity in the risk domain is located in the anterior part of the sulcus, while the identified cluster for the ambiguity domain is found close to the inferior frontal junction (IFJ) and extends into the inferior frontal gyrus (IFG) pars opercularis (see Figure 6). A recent cyto- and receptor-architectonic study reported that the IFS is divided into functionally and structurally different subregions (Ruland et al., 2022), in line with our finding of functional parcellation of the IFS between risk and ambiguity domains.

Previous literature suggests that the anteriorly located IFG/IFS is functionally associated with cognitive reasoning (Hartwigsen et al., 2018) and working memory (Ruland et al., 2022), while the more posterior IFS/IFJ cluster is linked to task switching activation (Derrfuss et al., 2005; Derrfuss et al., 2009), working memory (Bedini & Baldauf, 2021), and inhibitory control (Sebastian et al., 2013). It is likely that depending on how much information on reward probabilities is available, these processes are recruited differently during decision-making. Our results, therefore, indicate that different cognitive control and working memory demands might be reflected in the functional segregation of the IFS, as different parts of the region are involved in risk and ambiguity domains.

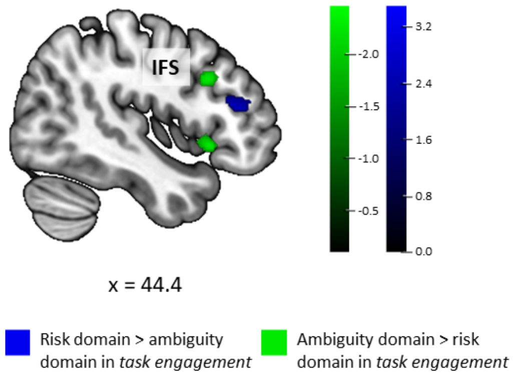

Supplementary Figure 1. IFS in risk and ambiguity domains. Overlays depict comparison of risk and ambiguity domains in task engagement. The blue cluster represents contrast risk > ambiguity contrast, while the green clusters represent contrast ambiguity > risk contrast.

Supplementary Table 1. Checklist for neuroimaging meta-analyses (Müller et al., 2018)

The research question is specifically defined	YES, and it includes the following contrasts:<br>1) *task engagement* – “task of interest>control task”, “task of interest>(implicit or explicit) baseline” in morality, risk, and ambiguity domains<br>2) *choice response* – “chosen option of interest>chosen option of no interest”, “chosen option>(implicit or explicit) baseline” in morality, risk, and ambiguity domains
The literature search was systematic	YES, it included the following keywords in the following databases:<br>[moral dilemma OR moral decision OR moral choice OR altruism OR altruistic decision OR altruistic choice OR ultimatum game OR donation task OR decision under risk OR risky choice OR risky decision OR ambiguity decision OR ambiguous decision OR decision under ambiguity OR ambiguous choice OR uncertainty decision OR decision under uncertainty OR uncertain decision OR uncertain choice] AND [fMRI OR positron] in “PubMed”, “Web of Science”, and “Science Direct”.
Detailed inclusion and exclusion criteria are included	YES
Sample overlap was taken into account	Yes, using the following method:<br>1) exclusion of publications, using the same data from the same sample as in the original publication;<br>2) were appropriate, separate experiments from the same sample were considered as one experiment.
All experiments use the same search coverage (state how brain coverage is assessed and how small volume corrections and conjunctions are taken into account)	Yes, the search coverage is the following:<br>Whole-brain analyses only.
Studies are converted to a common reference space	Yes, using the following conversion(s):<br>Linear transformation to MNI reference space.
Data extraction have been conducted by two investigators (ideal case) or double checked by the same investigator (state how double-checking was performed)	Yes, the following authors:<br>AA checked inclusion criteria<br>AA extracted the coordinates<br>AA extracted other info: sample size, stimuli description, imaging method, coordinate system.<br>AA and BD double-checked the following data: quality and inclusion criteria of questionable articles.<br><br>Student assistant HF doubled-checked the following data by comparing data file with

	original publications: extracted coordinates, sample size, mean age of participants by comparing original articles with data files. HF extracted other info: number of females in the study.
The study includes a table with at least the references, basic study description (e.g. for fMRI tasks: stimuli), contrasts and basic sample descriptions (e.g. size, mean age and gender distribution, specific characteristics) of the included studies, source of information (e.g. contact with authors), reference space	YES, and also the following data: number of foci included, original table number from which the data was extracted.
The study protocol was previously registered, and all analyses planned beforehand, including methods and parameters used for inference, correction for multiple testing, etc.	NO
The meta-analysis includes diagnostics	Yes, the following: Contributions

Supplementary Table 2. Included studies

Paper	Number of subjects	Mean age or age range	Task category	Task description	Contrast	Number of foci	Imaging method	Coordinate system
Ambiguous decision-making: Task engagement (N = 27 papers, 27 experiments, N = 454 foci)								
Behrens et al, 2007	18 (F: 9)	19-32	Probabilistic learning under ambiguity	Choosing between two rectangles and learning to identify a correct one.	Main effect of Decide (Suppl. Tab. 2)	48	fMRI	MNI
Bhanji et al, 2010	15 (F: 7)	21.9 (3.09)	Gambling under ambiguity	Guessing whether a randomly generated number would be lower or higher than the number in the cue.	Low choice certainty > high choice certainty (Tab. 2)	1	fMRI	MNI
Blackwood et al, 2004	8 (F: 0)	38	Guessing	Predicting from which jar a ball will be drawn and from which survey a word will be taken.	Uncertain > certain (Tab. 1)	6	fMRI	TAL
Blankenstein et al, 2017	50 (F: 25)	23.71 (2.56)	Gambling under ambiguity	Choosing between a gamble with ambiguous reward probability and a safe option.	Gamble ambiguity > fixation with ambiguity attitude as a positive Regressor (Tab. 4)	4	fMRI	MNI
Callan et al, 2009	14 (F: 7)	27.7	Spatial navigation under ambiguity	Turning a car at an intersection with obstacles blocking the view (DO) or the view enhanced by additional camera (DOC).	DO > DOC (Tab. 1)	18	fMRI	TAL
Causse et al, 2013	15 (N/A)	25.4 (2.45)	Spatial navigation under ambiguity	Choosing to land a plane or defer landing by judging the information provided in the instrument landing system.	High uncertainty > implicit baseline (Tab. 1)<br>High uncertainty > low uncertainty (Tab. 2)	9<br>24	fMRI	TAL
D'Acremont et al, 2013	23 (F: 10)	22	Probabilistic learning under ambiguity	Learning probabilities of cues and deciding whether to buy a gamble or not for a certain price.	Task > rest (Tab. S18)<br>Active > control decision (Tab. S18)	11<br>6	fMRI	MNI
D'Cruz et al, 2011	15 (F: 9)	25.4	Probabilistic learning under ambiguity	2-choice reversal learning task: guessing and learning the correct location of two identical stimuli presented.<br>4-choice reversal learning task: guessing and learning the correct location of four identical stimuli presented.	Unexpected non-reinforcement>expected positive reinforcement (2-choice Task) (Tab. 1)<br>Unexpected non-reinforcement>expected positive reinforcement (4-choice Task) (Tab. 1)	25<br>25	fMRI	MNI
Elliott et al, 1997	6 (F: 0)	27-50	Guessing	Guessing which 3 out of 6 buttons were assigned to be correct.	Guessing > planning (Tab. 1: rCBF decreases)	5	PET	TAL

Elliott et al, 1999	5 (F: 2)	29-41	Guessing	Guessing either the colour or the suit of the next card.	Guessing > reporting (Tab. 1)	12	fMRI	TAL
Farrar et al, 2018	19 (F: 12)	18-37	Guessing	Guessing to match card shape or colour when rules are unknown or known.	Uncertain > certain (unpublished coordinates received from the author corresponding to Fig. 4)	21	fMRI	MNI
Feinstein et al, 2006	16 (F: 8)	35.4	Guessing	Guessing whether the next card will be higher or lower.	Uncertain trials > certain trials (Tab. 1)	2	fMRI	TAL
Gloy et al, 2020	25 (F: 25)	21.04 (2.8)	Guessing	Deciding whether to take an umbrella by judging ambiguous weather information.	UNC > FIX (Tab. 1)	45	fMRI	MNI
Jung et al, 2014	24 (F: 8)	47.0 (11.6)	Guessing	Guessing whether the number of balls in odd or even when the balls are overlapped and not overlapped	Uncertain > certain (NO-PASS run 2) (Tab.1)<br>Uncertain > certain (PASS run 3) (Tab. 1)	11<br>7	fMRI	MNI
Krug et al, 2014	64 (F: 17)	34.7 (9.8)	Guessing	Learning probabilities of a jar and guessing from which jar the balls are being drawn.	Block information gathering > count (Tab. 2)<br>Event decision > count (Tab. 2)	21<br>13	fMRI	MNI
Mestres-Misse et al, 2017	22 (F: 11)	25.5 (3.1)	Probabilistic learning under ambiguity	Calculating the value of a sequence with ambiguous, unambiguous and incongruent instructions.	Ambiguous > unambiguous (Tab. S2)	8	fMRI	MNI
Miller et al, 2005	18 (F: 12)	18-44	Guessing	Predicting upcoming stimulus location.	Predict > detect 1-back (Tab. 2)	9	fMRI	TAL
Ohira, et al, 2010	16 (F: 0)	21.69 (2.25)	Gambling under ambiguity	Stochastic decision-making task: choosing from two symbols with ambiguous reward probabilities.	Random reward > control (Tab. 3b)	6	PET	MNI
Paulus et al, 2001	12 (F: 2)	40.0 (1.95)	Guessing	Predicting the side of a house where a car will appear.	Uncertain > control (Tab.1)	8	fMRI	TAL
Paulus et al, 2002	16 (F: 4)	38.9 (1.8)	Guessing	Predicting the side of a house where a car will appear.	Two-choice prediction > two-choice response (50% error) (Tab. 1)	6	fMRI	TAL
Paulus et al, 2004	19 (F: 5)	36.3 (6.2)	Probabilistic learning under ambiguity	Rock-Paper-Scissors Task: learning changing reward probabilities of the preferred option.	Early > late trials (Tab. 1)	3	fMRI	TAL
Shao et al, 2016	26 (F: 26)	19-28	Guessing	Guessing the position of the selected card after shuffling.	Self > PC (Tab. S2: Card selection 2/Control)	3	fMRI	MNI
Volz et al, 2003	16 (F: 5)	24.9	Guessing	Predicting which of the two figures is winning an imaginary battle.	Uncertain > certain (Tab. 1)	9	fMRI	TAL
Volz et al, 2004	12 (F: 7)	25.1	Probabilistic learning under ambiguity	Learning the probabilities and predicting which of the two figures is winning an imaginary battle.	Uncertain > certain (Tab. 3)	14	fMRI	TAL

Volz et al, 2005	15 (F: 10)	25.9	Probabilistic learning under ambiguity	Learning the probabilities and predicting which of the two figures is winning an imaginary battle.	Rule learning > control (Tab. 3)<br>Putative learning > control (Tab. 3)	10<br>12	fMRI	TAL
Wiehler et al, 2021	23 (F: 0)	25.91 (6.47)	Probabilistic learning under ambiguity	Four-armed Bandit task: learning reward probabilities while exploring four gamble options.	Task > implicit baseline (unpublished coordinates for control group only received from the author)	44	fMRI	MNI
Yoshida & Ishii, 2006	13 (F: 2)	23-28	Spatial navigation under ambiguity	Searching for the goal during maze exploration.	Goal-search task > visual search (Suppl. Tab. 1)	8	fMRI	TAL
Ambiguous decision-making: Choice response (13 papers, 13 experiments, N = 162 foci)								
Aberg et al, 2021	28 (F: 20)	27.57 (0.73)	Probabilistic learning under ambiguity	Three-armed Bandit task: learning reward probabilities while exploring four gamble options.	Explore > expoit (Suppl. Tab. 12)	18	fMRI	MNI
Addicott et al, 2014	24 (F: N/A)	36 (11)	Probabilistic learning under ambiguity	Six-armed Bandit task: learning reward probabilities while exploring four gamble options.	Explore > exploit (Tab. 2: Selection phase)	12	fMRI	MNI
Daw et al, 2006	14 (F: N/A)	N/A	Probabilistic learning under ambiguity	Four-armed Bandit task: learning reward probabilities while exploring four gamble options.	Explore > exploit (Fig. 3 and Suppl. Tab. 5)	6	fMRI	MNI
Furl et al, 2011	17 (F: N/A)	N/A	Guessing	Learning probabilities of a jar and guessing from which jar the balls are being drawn.	Draw choices > urn choices (Tab. 2)	5	fMRI	MNI
Gloy et al, 2020	25 (F: 25)	21.04 (2.8)	Guessing	Deciding whether to take an umbrella by judging ambiguous weather information.	Uncertain > certain (Tab. 1)	9	fMRI	MNI
Laureiro-Martinez et al, 2014	50 (F: 11)	33.182 (6.290)	Probabilistic learning under ambiguity	Four-armed Bandit task: learning reward probabilities while exploring four gamble options.	Explore > expoit (Tab. 3)	20	fMRI	MNI
Laureiro-Martinez et al, 2015	63 (F: 11)	33.333 (6.020)	Probabilistic learning under ambiguity	Four-armed Bandit task: learning reward probabilities while exploring four gamble options.	Exploration > exploitation (Tab. 3)	27	fMRI	TAL
Li et al, 2021	111 (F: 74)	21.8 (1.8)	Probabilistic learning under ambiguity	Four-armed Bandit task: learning reward probabilities while exploring four gamble options.	Exploration > exploitation (Tab. 1)	15	fMRI	MNI
Losecaat Vermeer et al, 2014	26 (F: 12)	22	Gambling under ambiguity	Mixed gamble task: gambling with 50/50 chance of reward after a gain/loss outcome for another task.	Play > pass (Tab. 3)	10	fMRI	MNI
Matthews et al, 2004	12 (F: 5)	34	Gambling under ambiguity	Lane risk-taking task: choosing between a gamble with 50/50 reward probability and a safe option.	risky choice > safe choice (Tab. 2)	4	fMRI	TAL
Purcell et al, 2021	18 (F: 10)	23.44 (2.91)	Gambling under ambiguity	Mixed gamble task: choosing between a gamble of 50/50 chance of reward and a sure reward	GambleReward_ChooseGamble > ChooseSureThing (Tab. S3)	3	fMRI	MNI

Rigoli et al, 2019	23 (F: 13)	37 (5)	Gambling under ambiguity	Mixed gamble task: choosing between a gamble of 50/50 chance of reward and a sure reward	Gambling > safe choice (Suppl. Tab. S3)	7	fMRI	MNI
Wiehler et al, 2021	23 (F: 0)	25.91 (6.47)	Probabilistic learning under ambiguity	Four-armed Bandit task: learning reward probabilities while exploring four gamble options.	Exploration > exploitation (unpublished coordinates for control group received from the author)	26	fMRI	MNI
Moral decision-making: Task engagement (46 papers, 48 experiments, N = 471 foci)								
Abe et al, 2014	25 (F: 14)	22	Deception	Deciding whether to lie or not in life-like harmful or helpful dilemma situation	Decision > control (Tab. 2)	10	fMRI	MNI
Bacha-Trams et al, 2017	30 (F: 30)	26	Sacrificial harm	Trolley dilemma task: choosing between rescuing different individuals (close kin, friend or unknown).	Decision > baseline including presentation of information (Tab. 2)	12	fMRI	MNI
Bahnemann et al, 2010	25 (F: 3)	26 (4.43)	Harm	Judging whether an action violated a moral norm.	MJ > PNJ (Tab. 2)	11	fMRI	MNI
Borg et al, 2006	24 (F: 11)	22.9	Sacrificial harm	Trolley dilemma task: choosing whether to sacrifice a group of people or not.	Moral > nonmoral (Tab. 4)	7	fMRI	MNI
Chakroff et al, 2016	20 (F: 7)	27	Harm	Judging wrongness/rightness of harmful and impure actions.	Harmful > impure (Suppl. Tab. 1.1.)	12	fMRI	MNI
Cui et al, 2021	33 (F: 15)	22.24 (3.73)	Harm	Investing money with different moral conflict levels for the investment targets.	HMC > LMC (Tab. 1)	27	fMRI	MNI
de Achaval et al, 2013	13 (F: 5)	28.5 (8.6)	Sacrificial harm	Trolley dilemma task: judging the appropriateness of an action to sacrifice a smaller number of humans in order to save a larger number.	Moral > nonmoral (Tab. 2: Controls)	6	fMRI	TAL
Duc et al, 2013	27 (F: 13)	23.2	Harm	Choosing between two moral transgressions.	Taboo & tragic > routine (Tab. S5)	6	fMRI	TAL
FeldmanHall et al, 2012	14 (F: 8)	25.9 (4.6)	Harm	Deciding whether to take a larger monetary reward and inflict more painful electric shocks to a receiver or a lower reward with a less painful electric shock.	Real PvG Decide > Non-Moral PvG Decide (Tab. 1)	4	fMRI	MNI
FeldmanHall et al, 2014	38 (F: 22)	24.6 (3.8)	Sacrificial harm	Trolley dilemma task: choosing whether to sacrifice something significant to save someone.	Difficult moral > difficult nonmoral (Tab. 4)	6	fMRI	MNI
Goucher-Lambert et al, 2017	11 (F: 4)	24 (3.4)	Harm	Choosing between two bottles with different environmental impact.	Environmental > control (Tab. 2)[a] [a] Coordinates in this table were reported in TAL RAI coordinate space and converted to TAL by us before transformation to MNI coordinate space for analysis purposes.	4	fMRI	TAL

Han et al, 2014	USA: 8 (F: 4) KR: 8 (F: 4)	28.59 (5.18)	Sacrificial harm	Trolley dilemma task: judging the appropriateness of an action to sacrifice a smaller number of humans in order to save a larger number.	Moral-personal > neutral (American sample) (Tab. S2)<br>Moral-impersonal > neutral (American sample) (Tab. S2)<br>Moral-personal > neutral (Korean sample) (Tab. S1)<br>Moral-impersonal > neutral (Korean sample) (Tab. S1)	11<br>16<br>18<br>10	fMRI	MNI
Harenski et al, 2010	14 (F: 14)	24.5 (3.94)	Sacrificial harm	Judging the severity of a sacrificial harm depicted in a picture.	Moral > nonmoral (Tab.1)	2	fMRI	MNI
Harenski et al, 2012	36 (F: 0)	27.3 (8.37)	Sacrificial harm	Judging the severity of a sacrificial harm depicted in a picture.	Moral > nonmoral (Tab.2)	11	fMRI	MNI
Harrison et al, 2012	73 (F: 31)	33.1 (8.3)	Sacrificial harm	Trolley dilemma task: choosing whether to sacrifice a smaller number of humans in order to save a larger number.	Combined moral > nonmoral (Tab. 2: Controls)	11	fMRI	MNI
Heekeren et al, 2003	8 (F: 1)	27.8 (4.5)	Harm	Judging the appropriateness of an action which either includes bodily harm or not.	Moral judgment > semantic judgment (Tab.2)	9	fMRI	TAL
Heekeren et al, 2005	12 (F: 2)	25.75 (1.54)	Harm	Judging the appropriateness of an action which either includes bodily harm or not.	Moral decision > semantic decision (Tab.2)	8	fMRI	MNI
Hu et al, 2021	39 (F: 24)	19.9 (1.9)	Deception	Deciding whether to take a bribe which hurts a third innocent party.	Bribe > control (Suppl. Tab. 1h)	3	fMRI	MNI
Kireev et al, 2013	12 (F: 0)	21-30	Deception	Making a computer to accept a deceptive claim or challenge a deceptive one.	Deception > truthful control (Tab. 1)	6	PET	MNI
Lelieveld et al, 2016	44 (F: 25)	21.0 (1.87)	Deception	Judging deceitfulness in reports of a dice roll game.	Unjustifiable lies > honest reports (Tab. 1)	6	fMRI	MNI
Li et al, 2011	27 (F: 20)	22.41 (2.5)	Harm	Deciding whether to lose a either a significant or a trivial item of value.	Vital > trivial (Tab. 1)	11	fMRI	MNI
Li et al, 2015	24 (F: 12)	20.42 (1.06)	Sacrificial harm	Judging moral blameworthiness of sacrificial harms.	II > NN (Tab. 2)	1	fMRI	MNI
Lim et al, 2017	19 (F: 10)	22.63 (1.30)	Sacrificial harm	Trolley dilemma task: judging the appropriateness of an action to sacrifice a smaller number of humans in order to save a larger number. Disgust primer applied.	Moral > nonmoral (Tab.1)	22	fMRI	MNI
Luo et al, 2006	20 (F: 11)	20-36	Sacrificial harm	Judging legality of sacrificial harm depicted in a picture.	Illegal > legal (Tab. 2)	9	fMRI	TAL
Moll et al, 2001	10 (F: 4)	24-43	Harm	Judging the wrongness and rightness of an action.	Moral > factual judgment (Tab. 1)	10	fMRI	TAL

Moll et al, 2002	7 (F: 4)	30.3 (4.7)	Harm	Judging the wrongness and rightness of an action.	Moral > neutral (Tab. 2)	3	fMRI	TAL
Ngo et al, 2019	16 (F: 0)	27.4 (6.3)	Harm	Deciding between a personal desire and a moral standard.	Moral > nonmoral (unpublished coordinates received from the author)	6	fMRI	MNI
Niemi et al, 2018	16 (F: 8)	22.44 (2.66)	Altruism	Judging moral praiseworthiness of an action.	Charity > Unspecified (Suppl. Tab. S1)	4	fMRI	MNI
Parkinson et al, 2011	38 (F: N/A)	N/A	Harm	Judging wrongness and rightness of harmful and dishonest actions.	Harmful transgressions > neutral scenarios (Tab. 1)<br>Dishonest transgressions > neutral scenarios (Tab. 1)	8<br>6	fMRI	TAL
Prehn et al, 2008	23 (F: 23)	25.17 (6.56)	Harm	Judging the wrongness and rightness of an action.	Socio-normative judgments > grammatical judgments (Tab. 3)	6	fMRI	MNI
Pujol et al, 2012	22 (F: 0)	40.6 (9.5)	Sacrificial harm	Trolley dilemma task: choosing whether to sacrifice a smaller number of humans in order to save a larger number.	Moral dilemma > nondilemma (controls) (Suppl. Tab. S2)	14	fMRI	MNI
Pulcu et al, 2014	15 (F: 9)	38.9 (5.9)	Altruism	Charitable donation task: deciding whether to donate to a charity with either a possibility for a personal loss, personal gain or no loss or gain.	Donation > fix (control) (Tab. 4)	3	fMRI	MNI
Reniers et al, 2012	24 (F: 0)	18-30	Harm	Judging the appropriateness of an action by agreeing to it or not.	Moral > nonmoral (Tab. 2)	6	fMRI	TAL
Sawe et al, 2015	20 (F: 12)	26 (6)	Altruism	Deciding whether to donate to an environmental charity in order to save valuable land.	Destructive > non-destructive use (Suppl. Tab. A4)	1	fMRI	TAL
Schleim et al, 2011	40 (F: 18)	31.05 (4.02)	Sacrificial harm	Trolley dilemma task: judging the appropriateness of an action to sacrifice a smaller number of humans in order to save a larger number.	Moral > neutral (Tab. 2)	6	fMRI	TAL
Schneider et al, 2013	28 (F: 13)	31.29 (9.03)	Harm	Deciding whether to agree with proposed action in a social-ethical dilemma.	Moral > BL (Tab. 3: healthy controls)	4	fMRI	MNI
Simmank et al, 2020	16 (F: 0)	28.3 (2.5)	Harm	Judging the wrongness and rightness of an action.	Moral > knowledge (Tab. 3)	2	fMRI	MNI
Smith et al, 2015	30 (F: 25)	26	Altruism	Judging the fairness of a spit of money between two social groups.	Experimental > control trials (Tab. 1)	7	fMRI	TALI
Sommer et al, 2010	12 (F: 7)	24.5 (2.1)	Harm	Deciding between a personal desire and a moral standard.	Moral conflicts > neutral conflict (Tab. 2)	6	fMRI	MNI
Sommer et al, 2014	16 (F: 9)	15.0 (0.82)	Harm	Deciding between a personal desire and a moral standard.	Moral conflict > social conflict (Tab. 2: Adults)	6	fMRI	MNI

Verdejo-Garcia et al, 2014	14 (F: 1)	30.1 (8.8)	Sacrificial harm	Trolley dilemma task: choosing whether to sacrifice a smaller number of humans in order to save a larger number.	Moral dilemma > control (Tab. 1: Control subjects)	8	fMRI	MNI
Wang et al, 2015	28 (F: 14)	22	Altruism	Judging the moral beauty of an action.	Scene moral aesthetic judgment > scene gender judgment (Tab. 1)	10	fMRI	MNI
White et al, 2017	23 (F: 17)<br>10 (F: 8)	25.08 (2.94)<br>24.31 (1.56)	Harm	Judging the wrongness and rightness of an action.	Harm > baseline<br>Harm/welfare > neutral (unpublished coordinates received from author)<br>*Coded as two separate experiments*	35<br>36	fMRI	MNI
Wu et al, 2011	18 (F: 13)	21.4	Deception	Judging the wrongness and rightness of a deceptive action.	BL (Bad act lie) > BT (Bad act truth) (Tab. 1)	8	fMRI	MNI
Young et al, 2007	10 (F: 5)	18-22	Harm	Judging permissibility of an action when protagonist's intentions/action outcomes are negative or neutral.	Negative belief > neutral belief (Suppl. Tab. 3)	2	fMRI	MNI
Zijlmans et al, 2018	22 (F: 0)	23.19	Sacrificial harm	Judging the severity of a sacrificial harm depicted in a picture.	Immoral > nonmoral (unpublished coordinates for the control group received from the author)	15	fMRI	MNI
Moral decision-making: Choice response (20 papers, 20 experiments, N = 192 foci)								
Abe et al, 2014	25 (F: 14)	22	Deception	Deciding whether to lie or not in life-like harmful or helpful dilemma situation	Dishonest/harmful > honest/harmful (Tab. 4)	3	fMRI	MNI
Chang et al, 2011	15 (F: 9)	18.5	Harm	One-shot trust game: acting as a trustee and deciding how much money to return to the investor.	Less > equal (Suppl. Tab. S2)	11	fMRI	MNI
Genevsky et al, 2013	22 (F: 6)	22	Altruism	Donation task: deciding whether to donate to an orphanage when either the name of a child, the photo or both are displayed.	Giving > not giving (Tab. 2)	21	fMRI	TAL
Greene et al, 2004	41 (F: 17)	N/A	Sacrificial harm	Trolley dilemma task: judging the appropriateness of an action to sacrifice a smaller number of humans in order to save a larger number.	Utilitarian > nonutilitarian (Tab. 4)	6	fMRI	TAL
Greening et al, 2014	18 (F: 9)	N/A	Altruism	Donation task: Deciding to assign a gain or a loss to either yourself or a charity.	Charity > self (Tab. 3: Main effect of choice)	2	fMRI	MNI
Güroğlu et al, 2014	18 (F: 7)	N/A	Harm	Dictator game: acting as an allocator and splitting the sum of money between themselves and an anonymous player.	Inequity > equity (Suppl. Tab. S1)	14	fMRI	MNI

Han et al, 2014	16 (F: 8)	28.59 (3.18)	Sacrificial harm	Trolley dilemma task: judging the appropriateness of an action to sacrifice a smaller number of humans in order to save a larger number.	Appropriate (Utilitarian) > Inappropriate (Suppl. Tab. S7: Moral-personal)<br>Appropriate (utilitarian) > inappropriate (Suppl. Tab. S7: Moral-impersonal)	6<br>7	fMRI	MNI
Kahane et al, 2012	16 (F: 9)	29.25	Sacrificial harm	Trolley dilemma task: deciding whether to sacrifice a smaller number of humans in order to save a larger number in intuitive and unintuitive scenarios.	Utilitarian > deontological (Suppl. Tab. S5: DI dilemmas)	8	fMRI	MNI
Kireev et al, 2013	24 (F: 14)	29.3 (6.5)	Deception	Making a computer to accept a deceptive claim or challenge a deceptive one.	DecClm > HonClm (Tab. 2)	21	fMRI	MNI
Park et al, 2017	36 (F: 16)	18-30	Altruism	Dictator game: acting as an allocator and splitting the sum of money between themselves and an anonymous player. Information about he partner's race and emotion was manipulated.	Giving > not giving (Tab. 1: Face period)	16	fMRI	TAL
Sommer et al, 2010	7 (F: N/A)	N/A	Harm	Deciding between a personal desire and a moral standard.	Immoral decision > moral decision (Fig. 3)	1	fMRI	MNI
Sommer et al, 2014	12 (F: N/A)	N/A	Altruism	Deciding between a personal desire and a moral standard.	Morally guided > personal desire-oriented (Tab. 4: Adults)	1	fMRI	MNI
Spence et al, 2008	17 (F: 9)	29.9 (7.1)	Deception	Choosing to answer questions deceitfully or truthfully.	Lie > truth (Tab. 1)	7	fMRI	MNI
Strombach et al, 2015	27 (F: 13)	25.03	Altruism	Donation task: Deciding to assign a gain to either yourself or yourself and another player.	Generous > selfish decision (Suppl. Tab. S2: Positive)	23	fMRI	MNI
Sun et al, 2016	25 (F: 25)	20-25	Sacrificial harm	One-shot trustee game: acting as a trustee and deciding how much money to return to the investor (either human or computer).	Dishonest > honest choice (Tab. 2: Main effect of choice)	6	fMRI	MNI
Sun et al, 2017	17 (F: 8)	24.05	Deception	Deciding whether to lie or not in reporting token number for the financial gain.	Lying > honest (Tab. 3)	4	fMRI	MNI
Tusche et al, 2016	33 (F: 15)	26	Altruism	Donation task: deciding whether to donate to a charity while foregoing a personal gain.	High donations > low donations (unpublished coordinated received from the author)	29	fMRI	MNI
Ty et al, 2017	18 (F: 9)	20.3 (1.52)	Sacrificial harm	Donation task: deciding whether to donate to a charity or not. Guilt inducing pictures included in the stimuli.	Choice harm 1 > help 1 (Tab. 2)	3	fMRI	MNI

Volz et al, 2015	34 (F: 17)	24.3 (2.6)	Deception	Lying or telling truth about the colour of a ball associated with a reward to another participant.	Simple deception > truth (Tab. 4)	8	fMRI	TAL
Yin et al, 2017	37 (F: 26)	19-33	Deception	Choosing to lie in order to benefit oneself or a charity or tell the truth to benefit oneself or a charity.	Charity benefiting lie > Charity benefiting truth (Tab. S2)	1	fMRI	MNI
Risky decision-making: Task engagement (23 papers, 24 experiments, N = 434 foci)								
Abidi et al, 2018	14 (F: 7)	23.50 (3.75)	Medical decision-making under risk	Choosing from 3 medications with different severity of side effects or benefits.	Severe > mild side effects (Tab. 1)	5	fMRI	MNI
Bjork et al, 2007	20 (F: 10)	28.5 (3.2)	Naturalistic risk-taking	Chicken game: pressing a button to collect rewards for as long as one dares in order not to be penalized.	Low penalty trials > no penalty trials (Tab. 2)<br>High-penalty trials > no penalty trials (Tab. 3)	22<br>21	fMRI	TAL
Bjork et al, 2008	17 (F: 7)	33.5	Naturalistic risk-taking	Chicken game: pressing a button to collect rewards for as long as one dares in order not to be penalized.	Low penalty trials > no penalty trials (Tab. 2: Controls)<br>High penalty trials > no penalty trials (Tab. 3: Controls)	22<br>23	fMRI	TAL
Blankenstein et al, 2017	50 (F: 25)	23.71 (2.56)	Gambling under risk	Choosing between a gamble with a probable reward and a safe option.	Gamble Risk > Fixation with Risk Attitude as a Positive Regressor (Tab. 3)	9	fMRI	MNI
Brevers et al, 2016	15 (F: 9)	22.07 (1.67)	Naturalistic risk-taking	Iowa Gambling Task: selecting from 4 card decks with different probabilities of reward.	Deck selection > implicit baseline (control group) (Tab. 2)	5	fMRI	MNI
Ernst et al, 2002	20 (F: 8)	30.4 (6.3)	Naturalistic risk-taking	Iowa Gambling Task: selecting from 4 card decks with different probabilities of reward.	Active task > control task (Tab. 1)	22	PET	MNI
Ernst et al, 2004	17 (F: 7)	28.9 (4.9)	Gambling under risk	Wheel of fortune task: choosing between low and high reward with different reward probabilities attached.	Monetary > control (Tab. 1: Selection)	19	fMRI	MNI
Ersche et al, 2005	15 (F: 6)	35.8 (9.0)	Gambling under risk	Cambridge risk task: choosing between two options associated with different probabilities of reward and punishment.	Risky decision-making > control (Tab. 4: Controls)	10	PET	TAL
Gathmann et al, 2014	19 (F: 9)	24.06 (5.07)	Gambling under risk	Game of Dice task: betting which number will be thrown by one to four dices. Combined with simultaneous 2n-back task for cognitive load.	GDT plus 2-back > 2-back (Tab. 3)	5	fMRI	MNI
Gentili et al, 2020	35 (F: 25)	23 (2)	Naturalistic risk-taking	Balloon Analog Risk Task: imploding a balloon and choosing either to cash out or pump further.	BART > resting state (Suppl. Tab. S1)	22	fMRI	TAL

Gowin et al, 2014	40 (F: 14)	35.6 (11.5)	Naturalistic risk-taking	Risky Gains Task: choosing between a safe option with a low reward or two following risky options where the proposed amount could be won or lost.	Risky gains task > baseline (Suppl. Tab. S3)	38	fMRI	TAL
Huettel, 2006	23 (F: 15)	18-29	Gambling under risk	Responding to five different shapes and choosing either to gamble for reward or skip.	Certain + reward risk > baseline (Tab. 1)	20	fMRI	MNI
Kano et al, 2011	13 (F: 0)	N/A	Naturalistic risk-taking	Iowa Gambling Task: selecting from 4 card decks with different probabilities of reward.	IGT 1-4 > control (Tab. 2: nonalexithymic group)	11	PET	TAL
Lawrence et al, 2009	15 (F: 0)	32.7 (10.1)	Naturalistic risk-taking	Iowa Gambling Task: selecting from 4 card decks with different probabilities of reward.	Active decision-making > control task (Tab. 1)	5	fMRI	MNI
Lee et al, 2021	TW: 51 (F: 26) USA: 27 (F: 15)	TW: 23.3 (2.32) USA: 21.4 (3.51)	Gambling under risk	Lottery choice task: accepting or rejecting lottery offers with varying probability and reward magnitude	Low probabilities > high probabilities (Taiwanese sample) Low probabilities > high probabilities (US sample) (unpublished coordinates received from the author)	4<br>3	fMRI	MNI
Rogers et al, 1999	8 (F: 0)	31.9 (2.0)	Gambling under risk	Rogers Decision Making Task: gambling a certain number of points based on a known likelihood for reward.	(4:2+5:1) > control (Tab. 2)	9	PET	TAL
Roy et al, 2011	23 (F: 16)	27.6 (7.)	Gambling under risk	Choosing between a reward and a loss with varying probabilities attached.	Prob40±60 > Prob100 (Tab. 1)	51	fMRI	MNI
Studer et al, 2012	39 (F: 0)	24.4 (4)	Gambling under risk	Roulette Betting Task: choosing the amount to bet on a roulette.	Active choice > no-choice (Tab. A1)	13	fMRI	MNI
van Leijenhorst et al, 2006	14 (F: 9)	21.5 (2.2)	Gambling under risk	Cake guessing task: guessing which cake flavour will be chosen by the computer with probabilities known.	HR_pos > LR_pos (Tab. 1: Adults)	16	fMRI	TAL
Vassena et al, 2014	20 (F: 12)	21.9 (20-26)	Gambling under risk	Choosing between a gamble with a probable reward and a safe option.	Choice > no choice (Tab. 1: Decision phase)	8	fMRI	MNI
Von Siebenthal et al, 2020	31 (F: 16)	27.7 (6.6)	Gambling under risk	The roulettes task: choosing between a smaller safer reward and larger riskier reward.	Selection phase > baseline (Tab. 2)	37	fMRI	MNI
Vorobyev et al, 2015	27 (F: 0)	18-19	Spatial navigation under risk	Computerized driving task: driving to reach the end goal as quickly as possible.	Go > baseline & Stop > baseline (Suppl. Tab. A)	18	fMRI	MNI
Weber and Huettel, 2008	23 (F: 11)	23	Gambling under risk	Choosing between two options with different probabilities, magnitudes of reward, and temporal delay conditions.	Risk > control & Risk > delay (Tab. 2)	16	fMRI	MNI

Risky decision-making: Choice response (32 papers, 32 experiments, N = 238 foci)

Bednarski et al, 2012	21 (F: 16)	25.0 (3.8)	Naturalistic risk-taking	Stop signal task (modified): choosing between speeding up and risking to make a mistake or slowing down.	Speed up > slow down (Tab. 3: Light and moderate drinkers)	18	fMRI	MNI
Burnette et al, 2021	16 (F: 5)	31.0 (9.05)	Naturalistic risk-taking	Balloon Analog Risk Task: imploding a balloon and choosing either to cash out or pump further.	Pumps (non-parametric) > control pumps (unpublished coordinates received from the author)	5	fMRI	MNI
Campbell-Meiklejohn et al, 2008	23 (F: 10)	25.68 (1.05)	Gambling under risk	Choose between gambling to recover a loss or quitting.	Chase losses > quit (Tab. 1)	4	fMRI	MNI
Canessa et al, 2011	24 (F: 12)	21.61 (2.75)	Gambling under risk	Wheel of fortune task: choosing between low and high reward with different reward probabilities attached.	Risky > non-risky (Suppl. Tab. 1)	6	fMRI	TAL
Christakou et al, 2009	19 (F: 0)	24.2 (3.93)	Naturalistic risk-taking	Iowa Gambling Task: selecting from 4 card decks with different probabilities of reward.	Disadvantageous > advantageous (Suppl. Tab. 1A)	6	fMRI	TAL
Cohen and Ranganath, 2005	17 (F: 9)	22-27	Gambling under risk	Choosing between a lower reward with higher probability and higher reward with lower probability of winning.	High > low-risk decision (Tab. 2)	4	fMRI	MNI
Cohen et al, 2005	16 (F: 7)	20-27	Gambling under risk	Choosing between a lower reward with higher probability and higher reward with lower probability of winning.	High > low risk (Tab. 1)	11	fMRI	MNI
Congdon et al, 2013	23 (F: 13)	25.65 (4.43)	Naturalistic risk-taking	Angling Risk Task: sequentially choosing between fishing with known probabilities or cashing-out.	ART risky choice > baseline (Tab. 3)	1	fMRI	MNI
Ekins et al, 2013	30 (F: 18)	18-40	Gambling under risk	Choosing between two lotteries with different reward probabilities attached.	Risk dom. trials > payoff dom. trials (Tab. 4: LxF)	1	fMRI	MNI
Ernst et al, 2004	17 (F: 7)	28.9 (4.9)	Gambling under risk	Wheel of fortune task: choosing between low and high reward with different reward probabilities attached.	Selection of high-reward/risk options > selection of low reward/risk options (Tab. 3)	20	fMRI	MNI
Fukui et al, 2005	14 (F: 1)	24.4 (1.45)	Naturalistic risk-taking	Iowa Gambling Task: selecting from 4 card decks with different probabilities of reward.	Risky decisions > safe decisions (Tab. 2)	1	fMRI	TAL
Fukunaga et al, 2018	25 (F: 14)	24.24 (3.00)	Gambling under risk	Choosing between a gamble with a probable reward and a safe option.	ChooseGambe > ChooseSureThing (Suppl. Tab. S8)	3	fMRI	MNI
Häusler et al, 2018	165 (F: 0)	39.0 (6.7)	Gambling under risk	Splitting money between a gamble (stock) with partial reward probability and a safe option (bond).	Stock > bond choice (Suppl. Tab. S3: gain domain)	2	fMRI	MNI
Jollant et al, 2010	12 (F: 0)	30	Naturalistic risk-taking	Iowa Gambling Task: selecting from 4 card decks with different probabilities of reward.	Risky > safe (Coordinates reported in section Whole-brain analysis: affective controls)	1	fMRI	TAL

Kolling et al, 2014	18 (F: 9)	22-36	Gambling under risk	Choosing between a lower reward with higher probability and higher reward with lower probability of winning.	Choice (riskier) > Choice (safer) (Suppl. Tab. S1)	4	fMRI	MNI
Lawrence et al, 2009	15 (F: 0)	32.7 (10.01)	Naturalistic risk-taking	Iowa Gambling Task: selecting from 4 card decks with different probabilities of reward.	Choices from risky > safe decks (Tab. 2)	10	fMRI	MNI
Lee et al, 2008	12 (F: 0)	29.9 (6.2)	Naturalistic risk-taking	Risky Gains Task: choosing between a safe option with a low reward or two sequential risky options where the proposed amount could be won or lost.	Risky > safe (Tab. 2: younger men)	4	fMRI	MNI
Liu et al, 2007	15 (F: 9)	26 (8)	Gambling under risk	Choosing to bet or to bank after seeing the total number of chips at bank.	Bet > bank (Tab. 1)	5	fMRI	MNI
Liu et al, 2017	27 (F: 0)	22.74 (2.35)	Gambling under risk	The cups task: Choosing between a gamble with partial reward probability and a safe option.	Risky choices > safe choices (Suppl. Tab. S2: HC group)	2	fMRI	MNI
Morawetz et al, 2019	29 (F: 13)	24.52 (4.25)	Gambling under risk	Risk Perception in Investment Decisions task: choosing between a safe investment and a risky investment after observing investment history. Emotion framing applied.	Risky choices > safe choices (Tab. 2)	3	fMRI	MNI
Paulus et al, 2003	17 (F: 6)	38.3 (1.4)	Naturalistic risk-taking	Risky Gains Task: choosing between a safe option with a low reward or two sequential risky options where the proposed amount could be won or lost.	Risky > safe responses (Tab. 1)	5	fMRI	TAL
Pletzer et al, 2016	59 (F: 41)	22.39 (5.14)	Naturalistic risk-taking<br>Gambling under risk	Balloon Analog Risk Task: imploding a balloon and choosing either to cash out or pump further.<br>Game of Dice task: betting which number will be thrown by one to four dices.	Risky > safe decision (coordinates reported in section 3.2.1.)<br>Risky > safe decision (coordinates reported in section 3.2.1.)	4<br>5	fMRI	MNI
Schonberg et al, 2012	16 (F: 10)	23.6 (2.9)	Naturalistic risk-taking	Balloon Analog Risk Task: imploding a balloon and choosing either to cash out or pump further.	Pumps (average) > control pumps (average) (Tab. 2)	2	fMRI	MNI
Symmonds et al, 2011	23 (F: 11)	24	Gambling under risk	Choosing between a gamble with a probable reward and a safe option.	Gamble > sure (Suppl. Tab. 5)	12	fMRI	MNI
van Leijenhorst et al, 2010	15 (F: 7)	21.60 (2.08)	Gambling under risk	Cake guessing task: guessing which cake flavour will be chosen by the computer with probabilities known.	HR > LR (Suppl. Tab. 1: adults)	1	fMRI	MNI
Vassena et al, 2014	20 (F: 12)	21.9 (20-26)	Gambling under risk	Choosing between a gamble with a probable reward and a safe option.	Risky gambles > safe gambles (Tab. 1)	5	fMRI	TAL

Vorobyev et al, 2015	34 (F: 0)	18-19	Spatial navigation under risk	Computerized driving task: driving to reach the end goal as quickly as possible.	Go > stop (Tab. 2)	24	fMRI	MNI
Wright et al, 2012	22 (F: 16)	22 (18-32)	Gambling under risk	Choosing between a gamble with a probable reward and a safe option.	Accept > reject (Tab. 1)	9	fMRI	MNI
Wright et al, 2013a	25 (F: 10)	24 (19-36)	Gambling under risk	Choosing between two lotteries with different reward magnitudes and probabilities attached.	Riskier > surer (Tab. 2a)	18	fMRI	MNI
Wright et al, 2013b	26 (F: 13)	24 (18-33)	Gambling under risk	Choosing between a gamble with a probable reward and a safe option.	Riskier > surer (Tab. 1)	23	fMRI	MNI
Xue et al, 2009	13 (F: 5)	23.6 (6)	Gambling under risk	The cups task: Choosing between a gamble with a probable reward and a safe option.	Risky > safe choices (Suppl. Tab. S3)	9	fMRI	MNI
Zhang et al, 2019	25 (F: 14)	20.64 (2.06)	Gambling under risk	The cups task (modified): Choosing between a gamble with a probable reward and a safe option for either self or other.	Risky > sure (Tab. 1)	10	fMRI	MNI

Supplementary Table 3. Combined risk and ambiguity domains – Uncertainty domain

Cluster #	Hemis	Region	BA	Z-value	Size (Voxels)	x	y	z
All studies in Uncertainty Domain in Task engagement, 51 experiment: risk (N = 24), ambiguity (N = 27)								
1	R/L	Paracingulate Gyrus; Superior Frontal Gyrus; Cingulate Gyrus, anterior division	32	5.78	691	4	24	42
2	R	Orbitofrontal Cortex; Insula	48	6.32	525	34	24	-6
3	R	Thalamus	-	6.58	313	10	-16	4
4	R	Middle Frontal Gyrus; Frontal Pole	45	6.07	301	42	36	22
5	L	Insula; Orbitofrontal Cortex	48	5.23	258	-34	18	4
6	L	Superior Parietal Lobule; Lateral Occipital Cortex, superior division	7	5.51	207	-28	-58	48
7	R	Lateral Occipital Cortex, superior division; Superior Parietal Lobule	7	4.63	202	34	-58	50
8	L	Precentral Gyrus; Middle Frontal Gyrus	6	4.29	137	-50	2	34
All studies in Uncertainty Domain in Response Choice								
1	R/L	Paracingulate Gyrus; Superior Frontal Gyrus; Cingulate Gyrus, anterior division	32	5.27	489	8	34	38
2	L	Caudate	25	5.02	222	-12	2	-8
3	L	Precentral Gyrus; Middle Frontal Gyrus	6	4.18	217	-42	2	34
4	L	Insula	47	6.30	178	-32	22	-2
5	R	Insula	47	4.94	167	32	24	-2
6	R	Caudate	25	5.85	152	10	10	0
7	R	Superior Frontal Gyrus; Middle Frontal Gyrus	6	5.25	120	26	0	56

Supplementary Table 4. Activation clusters across all experiments

Cluster #	Hemis	Region	BA	Z-value	Size (Voxels)	x	y	z
All studies								
1	R/L	Cingulate Gyrus, anterior division; Paracingulate Gyrus; Superior Frontal Gyrus	32	6.11	1472	4	22	44
2	L	Left Putamen; Insula; Left Caudate	47	7.36	780	-32	22	-2
3	R	Insula; Orbitofrontal Cortex	47	7.20	592	34	24	-2
4	R	Lateral Occipital Cortex, superior division; Supramarginal Gyrus, posterior division; Superior Parietal Lobule	40	5.63	510	44	-38	44
5	R/L	Thalamus	-	5.40	362	10	-16	4
6	L	Precentral Gyrus; Inferior Frontal Gyrus, pars opercularis	44	5.34	302	-48	8	30
7	L	Lateral Occipital Cortex, superior division; Superior Parietal Lobule	7	6.12	290	-28	-56	48
8	R	Frontal Pole	45	6.24	271	44	36	22
9	R	Caudate	25	5.94	266	10	10	0
10	R/L	Precuneous	-	5.44	197	0	-56	32
11	L	Lateral Occipital Cortex, superior division; Angular Gyrus	39	5.12	188	-48	-64	24
12	R	Middle Frontal Gyrus; Superior Frontal Gyrus	6	4.78	170	28	0	56

Supplementary Table 5. Activation clusters across all experiments for each domain

Cluster #	Hemis	Region	BA	Z-value	Size (Voxels)	x	y	z
All studies in morality domain								
1	R/L	Paracingulate Gyrus; Superior Frontal Gyrus	32	5.34	512	6	52	24
2	L	Supramarginal Gyrus, posterior division; Lateral Occipital Cortex, superior division; Angular Gyrus	39	6.27	388	-48	-64	24
3	R/L	Precuneous	-	6.43	335	0	-56	32
4	R	Temporal Pole	21	5.04	115	52	8	-26
All studies in risk domain								
1	R/L	Paracingulate Gyrus; Superior Frontal Gyrus	32	4.66	560	6	30	38
2	R	Caudate	25	6.25	306	10	10	0
3	R	Orbitofrontal Cortex; Insula; Inferior Frontal Gyrus, pars triangularis; Inferior Frontal Gyrus, pars opercularis; Frontal Operculum Cortex	47	5.66	265	30	22	-8
4	R	Lateral Occipital Cortex, superior division; Superior Parietal Lobule	7	5.00	256	28	-58	52
5	L	Precentral Gyrus; Inferior Frontal Gyrus, pars opercularis	44	4.60	204	-44	4	28
6	L	Accumbens	-	4.78	197	-10	6	-4
7	R	Frontal Pole; Middle Frontal Gyrus	45	5.80	152	44	36	20
8	L	Lateral Occipital Cortex, superior division; Superior Parietal Lobule	7	3.78	110	-16	-68	44
All studies in ambiguity domain								

1	R/L	Paracingulate Gyrus; Superior Frontal Gyrus	32	5.80	773	8	18	50
2	R	Orbitofrontal Cortex; Insula	47	6.27	440	34	24	-4
3	R/L	Thalamus	-	6.18	303	10	-16	4
4	L	Insula	47	7.00	288	-32	22	-2
5	R	Supramarginal Gyrus, posterior division; Superior Parietal Lobule	40	5.33	222	42	-38	46
6	R	Frontal Pole	46	5.82	206	40	38	24
7	L	Superior Parietal Lobule	7	5.09	205	-28	-56	46
8	L	Frontal Pole; Middle Frontal Gyrus	46	4.46	172	-36	42	22
9	L	Superior Parietal Lobule; Postcentral Gyrus	40	4.79	142	-40	-40	44
10	L	Thalamus; Brain Stem	-	4.65	123	-12	-16	6
11	R	Superior Frontal Gyrus; Middle Frontal Gyrus	6	5.18	116	26	0	56
12	R	Middle Frontal Gyrus; Inferior Frontal Gyrus, pars opercularis	44	4.65	104	50	16	32
13	L	Precentral Gyrus; Middle Frontal Gyrus	6	3.95	104	-46	4	34
14	L	Brain Stem	-	5.07	97	-4	-32	-14
Conjunction Morality Domain ∩ Risk Domain								
1	R	Superior Frontal Gyrus; Paracingulate Gyrus	32	3.50	16	6	48	28
Contrast Morality Domain > Risk Domain								
1	R/L	Paracingulate Gyrus; Frontal Pole; Superior Frontal Gyrus	9	3.29	315	-8	54	40
2	L	Angular Gyrus; Lateral Occipital Cortex, superior division	39	5.83	307	-46	-62	24
3	R/L	Precuneous; Cingulate Gyrus, posterior division	9	3.22	241	-8	-54	32
4	R	Temporal Pole	21	2.76	98	50	6	-30

Contrast Morality Domain < Risk Domain								
1	R/L	Paracingulate Gyrus	32	4.66	392	6	30	38
2	R	Caudate	-	3.13	228	6	10	6
3	R	Lateral Occipital Cortex, superior division; Superior Parietal Lobule	7	3.38	201	32	-64	46
4	L	Precentral Gyrus	6	3.67	156	-50	0	34
5	R	Insula; Orbitofrontal Cortex; Frontal Operculum Cortex	47	3.17	149	26	22	-6
6	R	Frontal Pole; Middle Frontal Gyrus	45	3.45	135	50	34	20
7	L	Putamen; Caudate	11	2.55	82	-18	14	-8
8	L	Lateral Occipital Cortex, superior division	19	2.74	74	-16	-72	42
9	R	Inferior Frontal Gyrus, pars triangularis	45	2.22	18	50	22	2
10	L	Lenticular Fasciculus	25	2.13	17	-8	2	-6
Conjunction Morality Domain ∩ Ambiguity Domain								
		no results						
Contrast Morality Domain > Ambiguity Domain								
1	R/L	Paracingulate Gyrus; Frontal Pole; Superior Frontal Gyrus	32	3.14	311	6	50	28
2	L	Supramarginal Gyrus, posterior division; Angular Gyrus	37	3.54	288	-48	-58	16
3	R/L	Precuneous	-	3.67	238	6	-60	32
4	R	Temporal Pole	21	2.34	99	52	8	-26
Contrast Morality Domain < Ambiguity Domain								
1	R/L	Paracingulate Gyrus; Superior Frontal Gyrus	32	5.80	729	8	18	50
2	R	Insula; Orbitofrontal Cortex	47	6.20	410	36	24	-6
3	R/L	Thalamus	0	5.64	303	10	-18	2
4	L	Insula	47	3.49	251	-34	20	2
5	R	Frontal Pole	46	5.64	205	40	40	26

6	L	Lateral Occipital Cortex, superior division; Superior Parietal Lobule	7	3.01	202	-28	-60	54
7	R	Superior Parietal Lobule; Supramarginal Gyrus, posterior division	2	2.85	171	40	-38	50
8	L	Frontal Pole; Middle Frontal Gyrus	46	3.35	159	-40	38	22
9	L	Superior Parietal Lobule; Supramarginal Gyrus, posterior division	40	3.94	133	-42	-42	44
10	R	Superior Frontal Gyrus	8	3.78	114	24	2	54
11	R	Middle Frontal Gyrus	44	4.25	104	50	14	34
12	L	Thalamus	-	3.26	102	-14	-18	4
13	L	Brain Stem	-	3.16	94	-6	-28	-18
14	L	Precentral Gyrus; Middle Frontal Gyrus	6	3.20	82	-46	2	36
Conjunction Risk Domain ∩ Ambiguity Domain								
1	R/L	Paracingulate Gyrus	32	4.66	277	6	30	38
2	R	Insula; Orbitofrontal Cortex; Frontal Operculum Cortex	47	5.26	187	32	22	-6
3	L	Precentral Gyrus; Middle Frontal Gyrus	44	3.73	57	-48	4	34
4	R	Frontal Pole	45	4.59	43	42	36	22
5	L	Superior Parietal Lobule	7	3.48	29	-24	-58	50
Contrast Risk Domain > Ambiguity Domain								
1	R/L	Caudate; Accumbens; Subcallosal Cortex	25	2.32	195	10	18	6
2	R	Lateral Occipital Cortex, superior division; Superior Parietal Lobule	7	2.51	71	28	-60	46
3	R	Paracingulate Gyrus; Superior Frontal Gyrus	32	2.07	37	4	46	28
4	R	Frontal Pole	45	2.15	24	48	36	14
5	L	Lateral Occipital Cortex, superior division	-	2.15	16	-16	-74	44
Contrast Risk Domain < Ambiguity Domain								
1	R/L	Thalamus	-	2.99	263	4	-22	-8

2	R/L	Paracingulate Gyrus; Superior Frontal Gyrus	-	3.38	222	2	16	52
3	L	Insula	47	3.41	151	-36	22	0
4	L	Frontal Pole; Middle Frontal Gyrus	46	3.26	138	-34	52	18
5	L	Superior Parietal Lobule; Postcentral Gyrus; Supramarginal Gyrus, anterior division; Supramarginal Gyrus, posterior division	40	3.54	137	-40	-40	40
6	R	Orbitofrontal Cortex	-	3.05	135	46	20	-8
7	R	Inferior Frontal Gyrus, pars opercularis; Middle Frontal Gyrus	44	3.38	101	52	18	30
8	R/L	Brain Stem	30	2.90	92	-8	-28	-20
9	R	Frontal Pole	46	2.90	77	34	40	24
10	R	Supramarginal Gyrus, posterior division	2	2.19	65	44	-36	50
11	L	Thalamus	-	2.84	61	-16	-18	6
12	R	Superior Frontal Gyrus	6	2.15	44	26	4	60
13	L	Lateral Occipital Cortex, superior division	7	1.97	36	-30	-60	44
14	R	Superior Parietal Lobule	40	2.03	16	34	-48	50
15	L	Superior Parietal Lobule	-	1.97	15	-32	-56	54

Supplementary Table 6. Task contributions in *task engagement* category across domains

Cluster name	Study	Task type	Control task type	Average contribution (%)
**Task engagement across domains**				
**Paracingulate Gyrus (R/L)**	**38 out of 98 experiments contributed:**			
	Behrens et al, 2007	Probabilistic learning under ambiguity	Low-level control condition (baseline)	0.85
	Bjork et al, 2007	Naturalistic risk-taking	High-level control condition	2.99
	Bjork et al, 2008	Naturalistic risk-taking	High-level control condition	2.55
	Brevers et al, 2016	Naturalistic risk-taking	Low-level control condition (baseline)	3.41
	Callan et al, 2009	Spatial navigation under ambiguity	High-level control condition	0.18
	Causse et al, 2013	Spatial navigation under ambiguity	Low-level control condition (baseline)	5.53
	Cui et al, 2021	Harm	High-level control condition	2.47
	d'Acremont et al, 2013	Probabilistic learning under ambiguity	Low-level control condition (baseline)	1.48
	D'Cruz et al, 2011	Probabilistic learning under ambiguity	High-level control condition	0.67
	de Achaval et al, 2013	Sacrificial harm	High-level control condition	1.75
	Elliott et al, 1999	Guessing	Low-level control condition	0.39
	Ernst et al, 2002	Naturalistic risk-taking	Low-level control condition	3.24
	Ernst et al, 2004	Gambling under risk	Low-level control condition	6.72
	Farrar et al, 2018	Guessing	High-level control condition	1.29
	FeldmanHall et al, 2012	Harm	High-level control condition	0.76
	Gentili et al, 2020	Naturalistic risk-taking	High-level control condition	0.36
	Gloy et al, 2020	Guessing	Low-level control condition (baseline)	8.33
	Gowin et al, 2014	Naturalistic risk-taking	Low-level control condition (baseline)	0.15
	Han et al, 2014 (American sample)	Sacrificial harm	High-level control condition	2.39
	Hu et al, 2021	Deception	High-level control condition	2.34
	Jung et al, 2014	Guessing	High-level control condition	6.39

	Kano et al, 2011	Naturalistic risk-taking	Low-level control condition	1.4
	Kireev et al, 2013	Deception	Low-level control condition	4.17
	Krug et al, 2014	Guessing	Low-level control condition	4.69
	Miller et al, 2005	Guessing	Low-level control condition	0.72
	Ohira, et al, 2010	Gambling under ambiguity	Low-level control condition	3.16
	Roy et al, 2011	Gambling under risk	High-level control condition	9.52
	Shao et al, 2016	Guessing	Low-level control condition	1.86
	Studer et al, 2012	Gambling under risk	Low-level control condition	1.56
	van Leijenhorst et al, 2006	Gambling under risk	High-level control condition	0.25
	Vassena et al, 2014	Gambling under risk	Low-level control condition	2.31
	Verdejo-Garcia et al, 2014	Sacrificial harm	Low-level control condition	2.37
	Volz et al, 2003	Guessing	High-level control condition	1.26
	Volz et al, 2004	Probabilistic learning under ambiguity	High-level control condition	1.19
	Volz et al, 2005	Probabilistic learning under ambiguity	High-level control condition	5.26
	Weber and Huettel, 2008	Gambling under risk	High-level control condition	2.8
	Yoshida & Ishii, 2006	Spatial navigation under ambiguity	Low-level control condition	2.63
	Young et al, 2007	Harm	High-level control condition	0.25
**Orbitofrontal Cortex (R)**	**32 out of 98 experiments contributed:**			
	Abidi et al, 2018	Medical decision-making under risk	High-level control condition	0.12
	Bacha-Trams et al, 2017	Sacrificial harm	Low-level control condition (baseline)	4.79
	Behrens et al, 2007	Probabilistic learning under ambiguity	Low-level control condition (baseline)	6.59
	Bhanji et al, 2010	Gambling under ambiguity	High-level control condition	3.05
	Bjork et al, 2007	Naturalistic risk-taking	High-level control condition	4.41
	Callan et al, 2009	Spatial navigation under ambiguity	High-level control condition	0.66
	Causse et al, 2013	Spatial navigation under ambiguity	Low-level control condition (baseline)	5.3
	d'Acremont et al, 2013	Probabilistic learning under ambiguity	Low-level control condition (baseline)	5.22
	D'Cruz et al, 2011	Probabilistic learning under ambiguity	High-level control condition	6.64
	de Achaval et al, 2013	Sacrificial harm	High-level control condition	0.22
	Duc et al, 2013	Harm	High-level control condition	1.06
	Elliott et al, 1999	Guessing	Low-level control condition	0.47
	Ernst et al, 2002	Naturalistic risk-taking	Low-level control condition	2.27
	Gentili et al, 2020	Naturalistic risk-taking	High-level control condition	4.39

	Gloy et al, 2020	Guessing	Low-level control condition (baseline)	6.6
	Harrison et al, 2012	Sacrificial harm	High-level control condition	0.09
	Jung et al, 2014	Guessing	High-level control condition	0.8
	Kano et al, 2011	Naturalistic risk-taking	Low-level control condition	2.24
	Miller et al, 2005	Guessing	Low-level control condition	1.19
	Ngo et al, 2019	Harm	High-level control condition	0.12
	Paulus et al, 2001	Guessing	Low-level control condition	4
	Rogers et al, 1999	Gambling under risk	Low-level control condition	3.52
	Roy et al, 2011	Gambling under risk	High-level control condition	8.84
	Sommer et al, 2014	Harm	High-level control condition	3.53
	van Leijenhorst et al, 2006	Gambling under risk	High-level control condition	1.2
	Vassena et al, 2014	Gambling under risk	Low-level control condition	2.24
	Volz et al, 2003	Guessing	High-level control condition	4.29
	Volz et al, 2004	Probabilistic learning under ambiguity	High-level control condition	2.61
	Volz et al, 2005	Probabilistic learning under ambiguity	High-level control condition	6.34
	Vorobyev et al, 2015	Spatial navigation under risk	Low-level control condition	2.74
	Wang et al, 2015	Altruism	High-level control condition	0.9
	Weber and Huettel, 2008	Gambling under risk	High-level control condition	3.39
**Lateral Occipital Cortex (L)**	**28 out of 98 experiments contributed:**			
	Bacha-Trams et al, 2017	Sacrificial harm	Low-level control condition (baseline)	5.12
	Bahnemann et al, 2010	Harm	High-level control condition	3.57
	Borg et al, 2006	Sacrificial harm	High-level control condition	0.69
	Chakroff et al, 2016	Harm	High-level control condition	0.69
	FeldmanHall et al, 2014	Sacrificial harm	High-level control condition	0.75
	Gowin et al, 2014	Naturalistic risk-taking	Low-level control condition (baseline)	2.36
	Han et al, 2014 (Korean sample)	Sacrificial harm	High-level control condition	0.58
	Harenski et al, 2012	Sacrificial harm	High-level control condition	5.49
	Harrison et al, 2012	Sacrificial harm	High-level control condition	6.35
	Heekeren et al, 2003	Harm	High-level control condition	5.44
	Heekeren et al, 2005	Harm	High-level control condition	1.46
	Lee et al, 2021(Taiwanese sample)	Gambling under risk	High-level control condition	2.34

	Lim et al, 2017	Sacrificial harm	High-level control condition	1.41
	Mestres-Misse et al, 2017	Probabilistic learning under ambiguity	High-level control condition	5.76
	Moll et al, 2001	Harm	High-level control condition	5.24
	Ngo et al, 2019	Harm	High-level control condition	2.64
	Parkinson et al, 2011	Harm	High-level control condition	4.7
	Paulus et al, 2002	Guessing	Low-level control condition	2.99
	Prehn et al, 2008	Harm	High-level control condition	1.22
	Pujol et al, 2012	Sacrificial harm	High-level control condition	3.83
	Schleim et al, 2011	Sacrificial harm	High-level control condition	7.02
	Schneider et al, 2013	Harm	High-level control condition	5.9
	Simmank et al, 2020	Sacrificial harm	Low-level control condition	5.12
	Sommer et al, 2010	Harm	High-level control condition	2.07
	Sommer et al, 2014	Harm	High-level control condition	4.63
	Verdejo-Garcia et al, 2014	Sacrificial harm	Low-level control condition	4.52
	Von Siebenthal et al, 2020	Gambling under risk	Low-level control condition (baseline)	5.06
	Zijlmans et al, 2018	Sacrificial harm	High-level control condition	2.8
**Insular Cortex (L)**	**23 out of 98 experiments contributed:**			
	Abidi et al, 2018	Medical decision-making under risk	High-level control condition	0.15
	Behrens et al, 2007	Probabilistic learning under ambiguity	Low-level control condition (baseline)	8.66
	Bjork et al, 2007	Naturalistic risk-taking	High-level control condition	6.2
	Bjork et al, 2008	Naturalistic risk-taking	High-level control condition	0.15
	Causse et al, 2013	Spatial navigation under ambiguity	Low-level control condition (baseline)	6.18
	D'Cruz et al, 2011	Probabilistic learning under ambiguity	High-level control condition	10.34
	de Achaval et al, 2013	Sacrificial harm	High-level control condition	4.79
	Ernst et al, 2002	Naturalistic risk-taking	Low-level control condition	2.12
	Gentili et al, 2020	Naturalistic risk-taking	High-level control condition	4.09
	Gloy et al, 2020	Guessing	Low-level control condition (baseline)	3.25
	Han et al, 2014 (American sample)	Sacrificial harm	High-level control condition	2.99
	Han et al, 2014 (Korean sample)	Sacrificial harm	High-level control condition	5.51
	Huettel, 2006	Gambling under risk	Low-level control condition (baseline)	0.93
	Jung et al, 2014	Guessing	High-level control condition	5.64

	Kano et al, 2011	Naturalistic risk-taking	Low-level control condition	2.41
	Krug et al, 2014	Guessing	Low-level control condition	7.89
	Sommer et al, 2014	Harm	High-level control condition	4.65
	Studer et al, 2012	Gambling under risk	Low-level control condition	0.71
	Vassena et al, 2014	Gambling under risk	Low-level control condition	1.07
	Volz et al, 2004	Probabilistic learning under ambiguity	High-level control condition	1.63
	Volz et al, 2005	Probabilistic learning under ambiguity	High-level control condition	6.64
	Von Siebenthal et al, 2020	Gambling under risk	Low-level control condition (baseline)	9.05
	Vorobyev et al, 2015	Spatial navigation under risk	Low-level control condition	4.81
**Precuneus (R/L)**	**20 out of 98 experiments contributed:**			
	Bahnemann et al, 2010	Harm	High-level control condition	7.31
	Gentili et al, 2020	Naturalistic risk-taking	High-level control condition	0.98
	Han et al, 2014 (American sample)	Sacrificial harm	High-level control condition	1.62
	Han et al, 2014 (Korean sample)	Sacrificial harm	High-level control condition	1.19
	Harenski et al, 2012	Sacrificial harm	High-level control condition	7.08
	Harrison et al, 2012	Sacrificial harm	High-level control condition	7.79
	Heekeren et al, 2005	Harm	High-level control condition	2.13
	Krug et al, 2014	Guessing	Low-level control condition	7.45
	Li et al, 2011	Sacrificial harm	High-level control condition	5.59
	Lim et al, 2017	Sacrificial harm	High-level control condition	0.72
	Mestres-Misse et al, 2017	Probabilistic learning under ambiguity	High-level control condition	1.04
	Moll et al, 2001	Harm	High-level control condition	1.09
	Ngo et al, 2019	Harm	High-level control condition	3.52
	Niemi et al, 2018	Altruism	High-level control condition	6.72
	Parkinson et al, 2011	Harm	High-level control condition	4.27
	Paulus et al, 2001	Guessing	Low-level control condition	0.15
	Pujol et al, 2012	Sacrificial harm	High-level control condition	4.83
	Schleim et al, 2011	Sacrificial harm	High-level control condition	6.87
	Schneider et al, 2013	Harm	High-level control condition	6.97
	Sommer et al, 2010	Harm	High-level control condition	3.12
	Sommer et al, 2014	Harm	High-level control condition	2.98
	Verdejo-Garcia et al, 2014	Sacrificial harm	Low-level control condition	5.6
	Von Siebenthal et al, 2020	Gambling under risk	Low-level control condition (baseline)	6.7

	Zijlmans et al, 2018	Sacrificial harm	High-level control condition	4.07
**Thalamus (R)**	**19 out of 98 experiments contributed:**			
	Abidi et al, 2018	Medical decision-making under risk	High-level control condition	5.46
	Behrens et al, 2007	Probabilistic learning under ambiguity	Low-level control condition (baseline)	9.36
	Bjork et al, 2007	Naturalistic risk-taking	High-level control condition	0.95
	Bjork et al, 2008	Naturalistic risk-taking	High-level control condition	9.49
	Causse et al, 2013	Spatial navigation under ambiguity	Low-level control condition (baseline)	9.58
	D'Cruz et al, 2011	Probabilistic learning under ambiguity	High-level control condition	9.4
	Elliott et al, 1999	Guessing	Low-level control condition	5.09
	Ernst et al, 2002	Naturalistic risk-taking	Low-level control condition	0.15
	Farrar et al, 2018	Guessing	High-level control condition	10.15
	Gentili et al, 2020	Naturalistic risk-taking	High-level control condition	1.91
	Gowin et al, 2014	Naturalistic risk-taking	Low-level control condition (baseline)	2.17
	Han et al, 2014 (American sample)	Sacrificial harm	High-level control condition	0.4
	Mestres-Misse et al, 2017	Probabilistic learning under ambiguity	High-level control condition	3.1
	Paulus et al, 2001	Guessing	Low-level control condition	5.6
	Roy et al, 2011	Gambling under risk	High-level control condition	0.39
	Sommer et al, 2010	Harm	High-level control condition	1.89
	Volz et al, 2003	Guessing	High-level control condition	8.56
	Volz et al, 2004	Probabilistic learning under ambiguity	High-level control condition	3.93
	Wiehler et al, 2021	Probabilistic learning under ambiguity	Low-level control condition (baseline)	12.14
	Yoshida & Ishii, 2006	Spatial navigation under ambiguity	Low-level control condition	0.11
**Frontal Pole (R)**	**23 out of 98 experiments contributed:**			
	Behrens et al, 2007	Probabilistic learning under ambiguity	Low-level control condition (baseline)	1.55
	Bjork et al, 2007	Naturalistic risk-taking	High-level control condition	2.81
	Bjork et al, 2008	Naturalistic risk-taking	High-level control condition	7.23
	Brevers et al, 2016	Naturalistic risk-taking	Low-level control condition (baseline)	0.67
	d'Acremont et al, 2013	Probabilistic learning under ambiguity	Low-level control condition (baseline)	8.26
	D'Cruz et al, 2011	Probabilistic learning under ambiguity	High-level control condition	8.33

	Elliott et al, 1999	Guessing	Low-level control condition	0.29
	Ernst et al, 2002	Naturalistic risk-taking	Low-level control condition	0.3
	Feinstein et al, 2006	Guessing	High-level control condition	6.07
	Gentili et al, 2020	Naturalistic risk-taking	High-level control condition	2.9
	Gloy et al, 2020	Guessing	Low-level control condition (baseline)	9.01
	Gowin et al, 2014	Naturalistic risk-taking	Low-level control condition (baseline)	0.13
	Han et al, 2014 (American sample)	Sacrificial harm	High-level control condition	0.3
	Kano et al, 2011	Naturalistic risk-taking	Low-level control condition	3
	Krug et al, 2014	Guessing	Low-level control condition	5.28
	Parkinson et al, 2011	Harm	High-level control condition	0.19
	Roy et al, 2011	Gambling under risk	High-level control condition	11.28
	van Leijenhorst et al, 2006	Gambling under risk	High-level control condition	1.45
	Vassena et al, 2014	Gambling under risk	Low-level control condition	8.37
	Volz et al, 2004	Probabilistic learning under ambiguity	High-level control condition	8.17
	Von Siebenthal et al, 2020	Gambling under risk	Low-level control condition (baseline)	5.5
	Wang et al, 2015	Altruism	High-level control condition	1.59
	Weber and Huettel, 2008	Gambling under risk	High-level control condition	7.12
**Superior Parietal Lobule (L)**	**22 out of 98 experiments contributed:**			
	Bacha-Trams et al, 2017	Sacrificial harm	Low-level control condition (baseline)	9.6
	Behrens et al, 2007	Probabilistic learning under ambiguity	Low-level control condition (baseline)	6.88
	Bjork et al, 2007	Naturalistic risk-taking	High-level control condition	8.41
	Blankenstein et al, 2017	Gambling under ambiguity	Low-level control condition (baseline)	0.19
	Causse et al, 2013	Spatial navigation under ambiguity	Low-level control condition (baseline)	8.48
	Chakroff et al, 2016	Harm	High-level control condition	1.07
	Ernst et al, 2004	Gambling under risk	Low-level control condition	4.92
	Farrar et al, 2018	Guessing	High-level control condition	6.45
	Gathmann et al, 2014	Gambling under risk	Low-level control condition	0.65
	Gloy et al, 2020	Guessing	Low-level control condition (baseline)	6.14

	Huettel, 2006	Gambling under risk	Low-level control condition (baseline)	2.56
	Jung et al, 2014	Guessing	High-level control condition	8.57
	Kireev et al, 2013	Deception	Low-level control condition	0.57
	Lelieveld et al, 2016	Deception	High-level control condition	0.98
	Rogers et al, 1999	Gambling under risk	Low-level control condition	0.18
	Studer et al, 2012	Gambling under risk	Low-level control condition	3.31
	van Leijenhorst et al, 2006	Gambling under risk	High-level control condition	2.73
	Vassena et al, 2014	Gambling under risk	Low-level control condition	2.85
	Volz et al, 2004	Probabilistic learning under ambiguity	High-level control condition	2.64
	Volz et al, 2005	Probabilistic learning under ambiguity	High-level control condition	9.64
	Weber and Huettel, 2008	Gambling under risk	High-level control condition	9.4
	Wu et al, 2011	Deception	High-level control condition	3.76
**Lateral Occipital Cortex (R)**	**22 out of 98 experiments contributed:**			
	Bacha-Trams et al, 2017	Sacrificial harm	Low-level control condition (baseline)	5.38
	Bjork et al, 2007	Naturalistic risk-taking	High-level control condition	0.88
	Bjork et al, 2008	Naturalistic risk-taking	High-level control condition	3.03
	Causse et al, 2013	Spatial navigation under ambiguity	Low-level control condition (baseline)	1.94
	Ernst et al, 2004	Gambling under risk	Low-level control condition	1.41
	Farrar et al, 2018	Guessing	High-level control condition	0.32
	Gathmann et al, 2014	Gambling under risk	Low-level control condition	8.28
	Gentili et al, 2020	Naturalistic risk-taking	High-level control condition	13.6
	Krug et al, 2014	Guessing	Low-level control condition	2.31
	Miller et al, 2005	Guessing	Low-level control condition	1.82
	Paulus et al, 2001	Guessing	Low-level control condition	0.15
	Rogers et al, 1999	Gambling under risk	Low-level control condition	3.67
	Roy et al, 2011	Gambling under risk	High-level control condition	5.37
	Studer et al, 2012	Gambling under risk	Low-level control condition	0.96
	van Leijenhorst et al, 2006	Gambling under risk	High-level control condition	6.63
	Vassena et al, 2014	Gambling under risk	Low-level control condition	0.23
	Volz et al, 2004	Probabilistic learning under ambiguity	High-level control condition	5.04
	Vorobyev et al, 2015	Spatial navigation under risk	Low-level control condition	12.54
	Weber and Huettel, 2008	Gambling under risk	High-level control condition	8.78

	White et al, 2017	Harm	High-level control condition	0.11
	Wu et al, 2011	Deception	High-level control condition	8.68
	Yoshida & Ishii, 2006	Spatial navigation under ambiguity	Low-level control condition	8.62
**Task engagement (Low-level control condition only)**				
**Paracingulate Gyrus, anterior division of Cingulate gyrus (R/L)**	**17 out of 35 experiments contributed:**			
	Behrens et al, 2007	Probabilistic learning under ambiguity	Low-level control condition (baseline)	1.48
	Brevers et al, 2016	Naturalistic risk-taking	Low-level control condition (baseline)	8.4
	Causse et al, 2013	Spatial navigation under ambiguity	Low-level control condition (baseline)	8.82
	Elliott et al, 1999	Guessing	Low-level control condition	0.88
	Ernst et al, 2002	Naturalistic risk-taking	Low-level control condition	8.9
	Gloy et al, 2020	Guessing	Low-level control condition (baseline)	17.99
	Gowin et al, 2014	Naturalistic risk-taking	Low-level control condition (baseline)	0.15
	Kano et al, 2011	Naturalistic risk-taking	Low-level control condition	4.21
	Kireev et al, 2013	Deception	Low-level control condition	8.62
	Krug et al, 2014	Guessing	Low-level control condition	7.59
	Miller et al, 2005	Guessing	Low-level control condition	2.23
	Ohira, et al, 2010	Gambling under ambiguity	Low-level control condition	9.05
	Shao et al, 2016	Guessing	Low-level control condition	0.23
	Studer et al, 2012	Gambling under risk	Low-level control condition	3.13
	Vassena et al, 2014	Gambling under risk	Low-level control condition	7.97
	Verdejo-Garcia et al, 2014	Sacrificial harm	Low-level control condition	3.93
	Yoshida & Ishii, 2006	Spatial navigation under ambiguity	Low-level control condition	6.36
**Orbitofrontal Cortex (R)**	**13 out of 35 experiments contributed:**			
	Bacha-Trams et al, 2017	Sacrificial harm	Low-level control condition (baseline)	9.68
	Behrens et al, 2007	Probabilistic learning under ambiguity	Low-level control condition (baseline)	16.83
	Causse et al, 2013	Spatial navigation under ambiguity	Low-level control condition (baseline)	10.51

	d'Acremont et al, 2013	Probabilistic learning under ambiguity	Low-level control condition (baseline)	12.58
	Elliott et al, 1999	Guessing	Low-level control condition	1.81
	Ernst et al, 2002	Naturalistic risk-taking	Low-level control condition	7.38
	Gloy et al, 2020	Guessing	Low-level control condition (baseline)	14.49
	Kano et al, 2011	Naturalistic risk-taking	Low-level control condition	3.43
	Miller et al, 2005	Guessing	Low-level control condition	2.52
	Paulus et al, 2001	Guessing	Low-level control condition	4.56
	Rogers et al, 1999	Gambling under risk	Low-level control condition	9.79
	Vassena et al, 2014	Gambling under risk	Low-level control condition	0.44
	Vorobyev et al, 2015	Spatial navigation under risk	Low-level control condition	5.99
**Posterior division of Supramarginal gyrus (R)**	**13 out of 35 experiments contributed:**			
	Causse et al, 2013	Spatial navigation under ambiguity	Low-level control condition (baseline)	0.18
	Ersche et al, 2005	Gambling under risk	Low-level control condition	0.22
	Gloy et al, 2020	Guessing	Low-level control condition (baseline)	11.7
	Kireev et al, 2013	Deception	Low-level control condition	9.39
	Krug et al, 2014	Guessing	Low-level control condition	29.75
	Miller et al, 2005	Guessing	Low-level control condition	1.41
	Paulus et al, 2001	Guessing	Low-level control condition	7.15
	Rogers et al, 1999	Gambling under risk	Low-level control condition	0.25
	Studer et al, 2012	Gambling under risk	Low-level control condition	2.85
	Von Siebenthal et al, 2020	Gambling under risk	Low-level control condition (baseline)	0.89
	Vorobyev et al, 2015	Spatial navigation under risk	Low-level control condition	20.2
	White et al, 2017 (partial)	Harm	Low-level control condition (baseline)	14.87
	Wiehler et al, 2021	Probabilistic learning under ambiguity	Low-level control condition (baseline)	0.98
**Task engagement (High-level control condition only) 63 experiments**				
**Angular gyrus, superior division of Lateral Occipital Cortex (L)**	**21 out of 63 experiments contributed:**			
	Bahnemann et al, 2010	Harm	High-level control condition	5.27

	FeldmanHall et al, 2014	Sacrificial harm	High-level control condition	3.09
	Han et al, 2014 (American sample)	Sacrificial harm	High-level control condition	0.22
	Han et al, 2014 (Korean sample)	Sacrificial harm	High-level control condition	2.23
	Harenski et al, 2012	Sacrificial harm	High-level control condition	7.89
	Harrison et al, 2012	Sacrificial harm	High-level control condition	8.86
	Heekeren et al, 2003	Harm	High-level control condition	5.7
	Heekeren et al, 2005	Harm	High-level control condition	2.57
	Lee et al, 2021 (Taiwanese sample)	Gambling under risk	High-level control condition	2.07
	Lim et al, 2017	Sacrificial harm	High-level control condition	2.84
	Mestres-Misse et al, 2017	Probabilistic learning under ambiguity	High-level control condition	7.27
	Moll et al, 2001	Harm	High-level control condition	7.66
	Ngo et al, 2019	Harm	High-level control condition	0.91
	Parkinson et al, 2011	Harm	High-level control condition	7.6
	Prehn et al, 2008	Harm	High-level control condition	2.05
	Pujol et al, 2012	Sacrificial harm	High-level control condition	5.61
	Schleim et al, 2011	Sacrificial harm	High-level control condition	7.59
	Schneider et al, 2013	Harm	High-level control condition	8.79
	Sommer et al, 2010	Harm	High-level control condition	4.06
	Sommer et al, 2014	Harm	High-level control condition	2.67
	Zijlmans et al, 2018	Sacrificial harm	High-level control condition	4.92
**Precuneus cortex, posterior division of Cingulate Cortex (R/L)**	**21 out of 63 experiments contributed:**			
	Bahnemann et al, 2010	Harm	High-level control condition	7.53
	Gentili et al, 2020	Naturalistic risk-taking	High-level control condition	1.61
	Han et al, 2014 (American sample)	Sacrificial harm	High-level control condition	2.35
	Han et al, 2014 (Korean sample)	Sacrificial harm	High-level control condition	1.73
	Harenski et al, 2012	Sacrificial harm	High-level control condition	8.43
	Harrison et al, 2012	Sacrificial harm	High-level control condition	9.21
	Heekeren et al, 2005	Harm	High-level control condition	2.33
	Li et al, 2011	Sacrificial harm	High-level control condition	6.72
	Lim et al, 2017	Sacrificial harm	High-level control condition	0.91
	Mestres-Misse et al, 2017	Probabilistic learning under ambiguity	High-level control condition	2.22
	Moll et al, 2001	Harm	High-level control condition	2.14
	Ngo et al, 2019	Harm	High-level control condition	4.96

	Niemi et al, 2018	Altruism	High-level control condition	6.9
	Parkinson et al, 2011	Harm	High-level control condition	4.99
	Pujol et al, 2012	Sacrificial harm	High-level control condition	6.62
	Schleim et al, 2011	Sacrificial harm	High-level control condition	8.04
	Schneider et al, 2013	Harm	High-level control condition	8.76
	Sommer et al, 2010	Harm	High-level control condition	4.24
	Sommer et al, 2014	Harm	High-level control condition	4.48
	Volz et al, 2005	Probabilistic learning under ambiguity	High-level control condition	0.13
	Zijlmans et al, 2018	Sacrificial harm	High-level control condition	5.53
**Paracingulate Gyrus, anterior division of Cingulate gyrus (R/L)**	**17 out of 63 experiments contributed:**			
	Bjork et al, 2007	Naturalistic risk-taking	High-level control condition	6.09
	Bjork et al, 2008	Naturalistic risk-taking	High-level control condition	7.69
	Callan et al, 2009	Spatial navigation under ambiguity	High-level control condition	0.62
	Cui et al, 2021	Harm	High-level control condition	7.3
	D'Cruz et al, 2011	Probabilistic learning under ambiguity	High-level control condition	2.91
	Ernst et al, 2004	Gambling under risk	High-level control condition	20.05
	Farrar et al, 2018	Guessing	High-level control condition	3.29
	Gentili et al, 2020	Naturalistic risk-taking	High-level control condition	0.64
	Han et al, 2014 (American sample)	Sacrificial harm	High-level control condition	1.7
	Jung et al, 2014	Guessing	High-level control condition	12.98
	Lelieveld et al, 2016	Deception	High-level control condition	0.28
	Roy et al, 2011	Gambling under risk	High-level control condition	17.69
	van Leijenhorst et al, 2006	Gambling under risk	High-level control condition	0.68
	Volz et al, 2003	Guessing	High-level control condition	2.78
	Volz et al, 2004	Probabilistic learning under ambiguity	High-level control condition	1.5
	Volz et al, 2005	Probabilistic learning under ambiguity	High-level control condition	6.21
	Weber and Huettel, 2008	Gambling under risk	High-level control condition	7.44
**Insula (R)**	**18 out of 63 experiments contributed:**			
	Abidi et al, 2018	Medical decision-making under risk	High-level control condition	0.27
	Bhanji et al, 2010	Gambling under ambiguity	High-level control condition	3.9
	Bjork et al, 2007	Naturalistic risk-taking	High-level control condition	11.62
	Bjork et al, 2008	Naturalistic risk-taking	High-level control condition	0.14
	Callan et al, 2009	Spatial navigation under ambiguity	High-level control condition	1.03

	Cui et al, 2021	Harm	High-level control condition	0.1
	D'Cruz et al, 2011	Probabilistic learning under ambiguity	High-level control condition	16.05
	de Achaval et al, 2013	Sacrificial harm	High-level control condition	0.14
	Duc et al, 2013	Harm	High-level control condition	2.07
	Gentili et al, 2020	Naturalistic risk-taking	High-level control condition	10.01
	Roy et al, 2011	Gambling under risk	High-level control condition	19.45
	Sommer et al, 2014	Harm	High-level control condition	6.67
	van Leijenhorst et al, 2006	Gambling under risk	High-level control condition	0.8
	Volz et al, 2003	Guessing	High-level control condition	3.64
	Volz et al, 2004	Probabilistic learning under ambiguity	High-level control condition	5.43
	Volz et al, 2005	Probabilistic learning under ambiguity	High-level control condition	10
	Wang et al, 2015	Altruism	High-level control condition	0.27
	Weber and Huettel, 2008	Gambling under risk	High-level control condition	8.36
**Superior Frontal Gyrus, Paracingulate Gyrus (R/L)**	**23 out of 63 experiments contributed:**			
	Bahnemann et al, 2010	Harm	High-level control condition	1.87
	Duc et al, 2013	Harm	High-level control condition	0.49
	Elliott et al, 1997	Guessing	High-level control condition	0.26
	Han et al, 2014 (American sample)	Sacrificial harm	High-level control condition	2.75
	Han et al, 2014 (Korean sample)	Sacrificial harm	High-level control condition	11.47
	Harenski et al, 2012	Sacrificial harm	High-level control condition	5.17
	Harrison et al, 2012	Sacrificial harm	High-level control condition	0.42
	Heekeren et al, 2003	Harm	High-level control condition	3.88
	Heekeren et al, 2005	Harm	High-level control condition	1.83
	Li et al, 2011	Sacrificial harm	High-level control condition	4.6
	Lim et al, 2017	Sacrificial harm	High-level control condition	0.88
	Mestres-Misse et al, 2017	Probabilistic learning under ambiguity	High-level control condition	6.9
	Ngo et al, 2019	Harm	High-level control condition	7.03
	Niemi et al, 2018	Altruism	High-level control condition	10.67
	Parkinson et al, 2011	Harm	High-level control condition	6.92
	Reniers et al, 2012	Harm	High-level control condition	6.57
	Schleim et al, 2011	Sacrificial harm	High-level control condition	7.02
	Schneider et al, 2013	Harm	High-level control condition	5.11
	Sommer et al, 2010	Harm	High-level control condition	7.69

	Sommer et al, 2014	Harm	High-level control condition	5.63
	Volz et al, 2004	Probabilistic learning under ambiguity	High-level control condition	0.88
	Wang et al, 2015	Altruism	High-level control condition	0.57
	Young et al, 2007	Harm	High-level control condition	1.26
**Paracingulate Gyrus (L)**	**12 out of 63 experiments contributed:**			
	Bjork et al, 2007	Naturalistic risk-taking	High-level control condition	0.32
	Bjork et al, 2008	Naturalistic risk-taking	High-level control condition	10.18
	Cui et al, 2021	Harm	High-level control condition	2.21
	D'Cruz et al, 2011	Probabilistic learning under ambiguity	High-level control condition	14.19
	Ernst et al, 2004	Gambling under risk	High-level control condition	13.18
	Farrar et al, 2018	Guessing	High-level control condition	4
	Gentili et al, 2020	Naturalistic risk-taking	High-level control condition	0.21
	Lee et al, 2021 (Taiwanese sample)	Gambling under risk	High-level control condition	18.3
	Lelieveld et al, 2016	Deception	High-level control condition	19.21
	Parkinson et al, 2011	Harm	High-level control condition	0.14
	Roy et al, 2011	Gambling under risk	High-level control condition	17.77
	Volz et al, 2005	Probabilistic learning under ambiguity	High-level control condition	0.19

Supplementary Table 7. Task contributions in *choice response* category across domains

Cluster name	Study	Task type	Average contribution (%)
**Choice response across domains**			
**Caudate (L)**	**19 out of 64 experiments contributed:**		
	Aberg et al, 2021	Probabilistic learning under ambiguity	6.59
	Campbell-Meiklejohn et al, 2008	Gambling under risk	2.9
	Chang et al, 2011	Harm	6.76
	Cohen and Ranganath, 2005	Gambling under risk	6.29
	Cohen et al, 2005	Gambling under risk	2.34
	Ernst et al, 2004	Gambling under risk	6.89
	Fukunaga et al, 2018	Gambling under risk	4.88
	Genevsky et al, 2013	Altruism	5.48
	Güroğlu et al, 2014	Harm	3.16
	Kireev et al, 2013	Deception	7.31
	Losecaat Vermeer et al, 2014	Gambling under ambiguity	4.7
	Matthews et al, 2004	Gambling under ambiguity	6.24
	Park et al, 2017	Altruism	0.23
	Pletzer et al, 2016	Naturalistic risk-taking/Gambling under risk	7.6
	Sun et al, 2016	Sacrificial harm	5.84
	Symmonds et al, 2011	Gambling under risk	5.9
	Tusche et al, 2016	Altruism	3.96
	Vorobyev et al, 2015	Spatial navigation under risk	12.51
	Xue et al, 2009	Gambling under risk	0.34
**Caudate (R)**	**18 out of 64 experiments contributed:**		
	Campbell-Meiklejohn et al, 2008	Gambling under risk	0.54
	Chang et al, 2011	Harm	12.17
	Cohen and Ranganath, 2005	Gambling under risk	9.21
	Cohen et al, 2005	Gambling under risk	6.8
	Ernst et al, 2004	Gambling under risk	10.22
	Genevsky et al, 2013	Altruism	2.34

	Güroğlu et al, 2014	Harm	3.56
	Liu et al, 2007	Gambling under risk	9.18
	Losecaat Vermeer et al, 2014	Gambling under ambiguity	10.26
	Park et al, 2017	Altruism	2.79
	Pletzer et al, 2016	Naturalistic risk-taking/Gambling under risk	8.46
	Spence et al, 2008	Deception	0.3
	Strombach et al, 2015	Altruism	0.19
	Sun et al, 2016	Sacrificial harm	5.99
	Symmonds et al, 2011	Gambling under risk	1.06
	Vorobyev et al, 2015	Spatial navigation under risk	8.11
	Wright et al, 2012	Gambling under risk	1.6
	Zhang et al, 2019	Gambling under risk	7.17
**Insular Cortex (L)**	**15 out of 64 experiments contributed:**		
	Kahane et al, 2012	Sacrificial harm	0.39
	Kireev et al, 2013	Deception	3.16
	Kolling et al, 2014	Gambling under risk	6.79
	Laureiro-Martinez et al, 2014	Probabilistic learning under ambiguity	4.34
	Laureiro-Martinez et al, 2015	Probabilistic learning under ambiguity	8
	Li et al, 2021	Probabilistic learning under ambiguity	10.59
	Losecaat Vermeer et al, 2014	Gambling under ambiguity	9.61
	Park et al, 2017	Altruism	4.98
	Pletzer et al, 2016	Naturalistic risk-taking/Gambling under risk	13.17
	Rigoli et al, 2019	Gambling under ambiguity	8.86
	Spence et al, 2008	Deception	0.68
	Strombach et al, 2015	Altruism	9.65
	Sun et al, 2016	Sacrificial harm	5.53
	Ty et al, 2017	Sacrificial harm	5.35
	Zhang et al, 2019	Gambling under risk	8.84
**Paracingulate Gyrus (R/L)**	**18 out of 64 experiments contributed:**		
	Abe et al, 2014	Deception	0.39
	Addicott et al, 2014	Probabilistic learning under ambiguity	0.22
	Bednarski et al, 2012	Naturalistic risk-taking	1.74
	Burnette et al, 2021	Naturalistic risk-taking	2.78

	Christakou et al, 2009	Naturalistic risk-taking	10
	Cohen and Ranganath, 2005	Gambling under risk	0.17
	Cohen et al, 2005	Gambling under risk	0.12
	Gloy et al, 2020	Guessing	7.43
	Kolling et al, 2014	Gambling under risk	10.14
	Laureiro-Martinez et al, 2014	Probabilistic learning under ambiguity	0.55
	Laureiro-Martinez et al, 2015	Probabilistic learning under ambiguity	16.5
	Liu et al, 2017	Gambling under risk	0.28
	Losecaat Vermeer et al, 2014	Gambling under ambiguity	11.6
	Pletzer et al, 2016	Naturalistic risk-taking/Gambling under risk	9.04
	Vassena et al, 2014	Gambling under risk	0.35
	Wright et al, 2012	Gambling under risk	15.45
	Xue et al, 2009	Gambling under risk	6.48
	Zhang et al, 2019	Gambling under risk	6.61
**Insular Cortex (R)**	**15 out of 64 experiments contributed:**		
	Güroğlu et al, 2014	Harm	0.26
	Kolling et al, 2014	Gambling under risk	0.14
	Laureiro-Martinez et al, 2014	Probabilistic learning under ambiguity	5.95
	Laureiro-Martinez et al, 2015	Probabilistic learning under ambiguity	0.78
	Li et al, 2021	Probabilistic learning under ambiguity	13.72
	Losecaat Vermeer et al, 2014	Gambling under ambiguity	11.17
	Park et al, 2017	Altruism	12.2
	Paulus et al, 2003	Naturalistic risk-taking	10.97
	Pletzer et al, 2016	Naturalistic risk-taking/Gambling under risk	12.5
	Rigoli et al, 2019	Gambling under ambiguity	10.69
	Spence et al, 2008	Deception	0.23
	Sun et al, 2016	Sacrificial harm	5.68
	Symmonds et al, 2011	Gambling under risk	0.12
	Wright et al, 2013a	Gambling under risk	6.43
	Zhang et al, 2019	Gambling under risk	9.16
**Precentral Gyrus (L)**	**12 out of 64 experiments contributed:**		
	Aberg et al, 2021	Probabilistic learning under ambiguity	13.69
	Addicott et al, 2014	Probabilistic learning under ambiguity	8.14

	Burnette et al, 2021	Naturalistic risk-taking	8.84
	Daw et al, 2006	Probabilistic learning under ambiguity	1.38
	Ernst et al, 2004	Gambling under risk	4.93
	Gloy et al, 2020	Guessing	8.58
	Laureiro-Martinez et al, 2015	Probabilistic learning under ambiguity	12.5
	Liu et al, 2017	Gambling under risk	9.94
	Losecaat Vermeer et al, 2014	Gambling under ambiguity	2.19
	Park et al, 2017	Altruism	12.03
	Wright et al, 2013b	Gambling under risk	13.27
	Zhang et al, 2019	Gambling under risk	4.43
**Superior Frontal Gyrus (R)**	**11 out of 64 experiments contributed:**		
	Aberg et al, 2021	Probabilistic learning under ambiguity	17.13
	Addicott et al, 2014	Probabilistic learning under ambiguity	10.76
	Ernst et al, 2004	Gambling under risk	0.36
	Kireev et al, 2013	Deception	8.22
	Laureiro-Martinez et al, 2014	Probabilistic learning under ambiguity	17.88
	Laureiro-Martinez et al, 2015	Probabilistic learning under ambiguity	16.2
	Li et al, 2021	Probabilistic learning under ambiguity	9.26
	Strombach et al, 2015	Altruism	0.52
	Vorobyev et al, 2015	Spatial navigation under risk	3.47
	Wiehler et al, 2021	Probabilistic learning under ambiguity	15.71
	Wright et al, 2013b	Gambling under risk	0.45

Supplementary Table 8. Task contributions in *task engagement* category for morality, risk, and ambiguity domains

Cluster name	Study	Task type	Control task type	Average contribution (%)
**Task engagement: Morality domain**				
**Superior Frontal Gyrus**	**23 out of 48 experiments contributed:**			
	Bahnemann et al, 2010	Harm	High-level control condition	3.35
	Duc et al, 2013	Harm	High-level control condition	2.82
	Han et al, 2014 (American sample)	Sacrificial harm	High-level control condition	3.88
	Han et al, 2014 (Korean sample)	Sacrificial harm	High-level control condition	8.67
	Harenski et al, 2012	Sacrificial harm	High-level control condition	3.71
	Harrison et al, 2012	Sacrificial harm	High-level control condition	1.96
	Heekeren et al, 2003	Harm	High-level control condition	3.06
	Heekeren et al, 2005	Harm	High-level control condition	1.96
	Kireev et al, 2013	Deception	Low-level control condition	0.25
	Li et al, 2011	Sacrificial harm	High-level control condition	6.67
	Lim et al, 2017	Sacrificial harm	High-level control condition	1.4
	Ngo et al, 2019	Harm	High-level control condition	4.86
	Niemi et al, 2018	Altruism	High-level control condition	8.13
	Parkinson et al, 2011	Harm	High-level control condition	7.03
	Reniers et al, 2012	Harm	High-level control condition	4.44
	Schleim et al, 2011	Sacrificial harm	High-level control condition	6.24
	Schneider et al, 2013	Harm	High-level control condition	6.4
	Simmank et al, 2020	Sacrificial harm	Low-level control condition	5.97
	Sommer et al, 2010	Harm	High-level control condition	5.44
	Sommer et al, 2014	Harm	High-level control condition	5.66
	Verdejo-Garcia et al, 2014	Sacrificial harm	Low-level control condition	5.01
	Wang et al, 2015	Altruism	High-level control condition	1.79
	Young et al, 2007	Harm	High-level control condition	1.23
**Angular Gyrus (L)**	**24 out of 48 experiments contributed:**			
	Bacha-Trams et al, 2017	Sacrificial harm	Low-level control condition (baseline)	5.13

	Bahnemann et al, 2010	Harm	High-level control condition	4.33
	Borg et al, 2006	Sacrificial harm	High-level control condition	0.97
	Chakroff et al, 2016	Harm	High-level control condition	0.95
	FeldmanHall et al, 2014	Sacrificial harm	High-level control condition	4.1
	Han et al, 2014 (American sample)	Sacrificial harm	High-level control condition	0.36
	Han et al, 2014 (Korean sample)	Sacrificial harm	High-level control condition	3.1
	Harenski et al, 2012	Sacrificial harm	High-level control condition	6.01
	Harrison et al, 2012	Sacrificial harm	High-level control condition	6.21
	Heekeren et al, 2003	Harm	High-level control condition	4.7
	Heekeren et al, 2005	Harm	High-level control condition	2.48
	Lim et al, 2017	Sacrificial harm	High-level control condition	4.22
	Moll et al, 2001	Harm	High-level control condition	5.84
	Ngo et al, 2019	Harm	High-level control condition	2.86
	Parkinson et al, 2011	Harm	High-level control condition	6.09
	Prehn et al, 2008	Harm	High-level control condition	2.55
	Pujol et al, 2012	Sacrificial harm	High-level control condition	4.93
	Schleim et al, 2011	Sacrificial harm	High-level control condition	6.17
	Schneider et al, 2013	Harm	High-level control condition	6.28
	Simmank et al, 2020	Sacrificial harm	Low-level control condition	4.69
	Sommer et al, 2010	Harm	High-level control condition	3.84
	Sommer et al, 2014	Harm	High-level control condition	4.4
	Verdejo-Garcia et al, 2014	Sacrificial harm	Low-level control condition	5.15
	Zijlmans et al, 2018	Sacrificial harm	High-level control condition	4.58
**Precuneus (R/L)**	**20 out of 48 experiments contributed:**			
	Bahnemann et al, 2010	Harm	High-level control condition	7.15
	Goucher-Lambert et al, 2017	Harm	High-level control condition	0.14
	Han et al, 2014 (American sample)	Sacrificial harm	High-level control condition	2.95
	Han et al, 2014 (Korean sample)	Sacrificial harm	High-level control condition	2.19
	Harenski et al, 2012	Sacrificial harm	High-level control condition	6.95
	Harrison et al, 2012	Sacrificial harm	High-level control condition	8.16
	Heekeren et al, 2005	Harm	High-level control condition	1.79
	Li et al, 2011	Sacrificial harm	High-level control condition	6.42
	Lim et al, 2017	Sacrificial harm	High-level control condition	0.56

	Moll et al, 2001	Harm	High-level control condition	1.46
	Ngo et al, 2019	Harm	High-level control condition	6
	Niemi et al, 2018	Altruism	High-level control condition	6.24
	Parkinson et al, 2011	Harm	High-level control condition	5.12
	Pujol et al, 2012	Sacrificial harm	High-level control condition	6.5
	Schleim et al, 2011	Sacrificial harm	High-level control condition	7
	Schneider et al, 2013	Harm	High-level control condition	7.59
	Sommer et al, 2010	Harm	High-level control condition	5.35
	Sommer et al, 2014	Harm	High-level control condition	5.49
	Verdejo-Garcia et al, 2014	Sacrificial harm	Low-level control condition	6.43
	Zijlmans et al, 2018	Sacrificial harm	High-level control condition	6.51
**Temporal Pole (R)**	**9 experiments out of 48 contributed:**			
	Abe et al, 2014	Deception	High-level control condition	17.46
	Bahnemann et al, 2010	Harm	High-level control condition	16
	Heekeren et al, 2003	Harm	High-level control condition	9.46
	Heekeren et al, 2005	Harm	High-level control condition	13.06
	Li et al, 2011	Sacrificial harm	High-level control condition	5.02
	Moll et al, 2001	Harm	High-level control condition	8.86
	Prehn et al, 2008	Harm	High-level control condition	7.86
	Sommer et al, 2010	Harm	High-level control condition	9.96
	White et al, 2017	Harm	High-level control condition	12.21
**Task engagement: Risk domain**				
**Lateral Occipital Cortex (R)**	**11 out of 24 experiments contributed:**			
	Bjork et al, 2007	Naturalistic risk-taking	High-level control condition	0.98
	Bjork et al, 2008	Naturalistic risk-taking	High-level control condition	9.82
	Ernst et al, 2004	Gambling under risk	Low-level control condition	7.73
	Gathmann et al, 2014	Gambling under risk	Low-level control condition	11.38
	Gentili et al, 2020	Naturalistic risk-taking	High-level control condition	13.21
	Rogers et al, 1999	Gambling under risk	Low-level control condition	8.08
	Roy et al, 2011	Gambling under risk	High-level control condition	12.6
	van Leijenhorst et al, 2006	Gambling under risk	High-level control condition	9.14
	Vassena et al, 2014	Gambling under risk	Low-level control condition	3.71

	Vorobyev et al, 2015	Spatial navigation under risk	Low-level control condition	12.72
	Weber and Huettel, 2008	Gambling under risk	High-level control condition	10.5
**Paracingulate Gyrus (R/L)**	**9 out of 24 experiments contributed:**			
	Bjork et al, 2007	Naturalistic risk-taking	High-level control condition	10.23
	Bjork et al, 2008	Naturalistic risk-taking	High-level control condition	7.76
	Brevers et al, 2016	Naturalistic risk-taking	Low-level control condition (baseline)	12.66
	Ernst et al, 2002	Naturalistic risk-taking	Low-level control condition	0.45
	Ernst et al, 2004	Gambling under risk	Low-level control condition	25.85
	Gentili et al, 2020	Naturalistic risk-taking	High-level control condition	0.25
	Roy et al, 2011	Gambling under risk	High-level control condition	24.84
	Vassena et al, 2014	Gambling under risk	Low-level control condition	6.51
	Weber and Huettel, 2008	Gambling under risk	High-level control condition	11.17
**Middle Frontal Gyrus (L)**	**11 out of 24 experiments contributed:**			
	Bjork et al, 2007	Naturalistic risk-taking	High-level control condition	11.2
	Bjork et al, 2008	Naturalistic risk-taking	High-level control condition	18.37
	Ernst et al, 2004	Gambling under risk	Low-level control condition	13.84
	Gentili et al, 2020	Naturalistic risk-taking	High-level control condition	0.21
	Gowin et al, 2014	Naturalistic risk-taking	Low-level control condition (baseline)	0.14
	Roy et al, 2011	Gambling under risk	High-level control condition	16.47
	Studer et al, 2012	Gambling under risk	Low-level control condition	8.1
	van Leijenhorst et al, 2006	Gambling under risk	High-level control condition	0.18
	Vassena et al, 2014	Gambling under risk	Low-level control condition	11.22
	Von Siebenthal et al, 2020	Gambling under risk	Low-level control condition (baseline)	7.04
	Weber and Huettel, 2008	Gambling under risk	High-level control condition	13.23
**Middle Frontal Gyrus (R)**	**11 out of 24 experiments contributed:**			
	Bjork et al, 2007	Naturalistic risk-taking	High-level control condition	0.68
	Bjork et al, 2008	Naturalistic risk-taking	High-level control condition	16.77
	Gentili et al, 2020	Naturalistic risk-taking	High-level control condition	0.11
	Gowin et al, 2014	Naturalistic risk-taking	Low-level control condition (baseline)	0.58
	Kano et al, 2011	Naturalistic risk-taking	Low-level control condition	8.72
	Roy et al, 2011	Gambling under risk	High-level control condition	17.01

	van Leijenhorst et al, 2006	Gambling under risk	High-level control condition	6.88
	Vassena et al, 2014	Gambling under risk	Low-level control condition	18.58
	Von Siebenthal et al, 2020	Gambling under risk	Low-level control condition (baseline)	11.78
	Vorobyev et al, 2015	Spatial navigation under risk	Low-level control condition	0.12
	Weber and Huettel, 2008	Gambling under risk	High-level control condition	18.68
Insular Cortex (R)	**11 out of 24 experiments contributed:**			
	Abidi et al, 2018	Medical decision-making under risk	High-level control condition	0.46
	Bjork et al, 2007	Naturalistic risk-taking	High-level control condition	20.73
	Ernst et al, 2002	Naturalistic risk-taking	Low-level control condition	0.77
	Gentili et al, 2020	Naturalistic risk-taking	High-level control condition	11.16
	Kano et al, 2011	Naturalistic risk-taking	Low-level control condition	7.94
	Rogers et al, 1999	Gambling under risk	Low-level control condition	5.41
	Roy et al, 2011	Gambling under risk	High-level control condition	30.2
	van Leijenhorst et al, 2006	Gambling under risk	High-level control condition	1.09
	Vassena et al, 2014	Gambling under risk	Low-level control condition	8.84
	Vorobyev et al, 2015	Spatial navigation under risk	Low-level control condition	0.47
	Weber and Huettel, 2008	Gambling under risk	High-level control condition	12.85
**Task engagement: Ambiguity domain**				
**Paracingulate Gyrus (R/L)**	**16 out of 27 experiments contributed:**			
	Behrens et al, 2007	Probabilistic learning under ambiguity	Low-level control condition (baseline)	2.12
	Causse et al, 2013	Spatial navigation under ambiguity	Low-level control condition (baseline)	10.97
	d'Acremont et al, 2013	Probabilistic learning under ambiguity	Low-level control condition (baseline)	5.11
	D'Cruz et al, 2011	Probabilistic learning under ambiguity	High-level control condition	0.91
	Elliott et al, 1999	Guessing	Low-level control condition	0.12
	Farrar et al, 2018	Guessing	High-level control condition	5.2
	Gloy et al, 2020	Guessing	Low-level control condition (baseline)	12.61
	Jung et al, 2014	Guessing	High-level control condition	11.16
	Krug et al, 2014	Guessing	Low-level control condition	13.62

	Miller et al, 2005	Guessing	Low-level control condition	0.51
	Ohira, et al, 2010	Gambling under ambiguity	Low-level control condition	7.03
	Shao et al, 2016	Guessing	Low-level control condition	5.1
	Volz et al, 2003	Guessing	High-level control condition	5.13
	Volz et al, 2004	Probabilistic learning under ambiguity	High-level control condition	4.65
	Volz et al, 2005	Probabilistic learning under ambiguity	High-level control condition	12.41
	Yoshida & Ishii, 2006	Spatial navigation under ambiguity	Low-level control condition	3.35
**Orbitofrontal Cortex (R)**	**14 out of 27 experiments contributed:**			
	Behrens et al, 2007	Probabilistic learning under ambiguity	Low-level control condition (baseline)	12.47
	Bhanji et al, 2010	Gambling under ambiguity	High-level control condition	6.32
	Callan et al, 2009	Spatial navigation under ambiguity	High-level control condition	0.91
	Causse et al, 2013	Spatial navigation under ambiguity	Low-level control condition (baseline)	9.57
	d'Acremont et al, 2013	Probabilistic learning under ambiguity	Low-level control condition (baseline)	9.91
	D'Cruz et al, 2011	Probabilistic learning under ambiguity	High-level control condition	11.11
	Elliott et al, 1999	Guessing	Low-level control condition	0.47
	Gloy et al, 2020	Guessing	Low-level control condition (baseline)	12.9
	Jung et al, 2014	Guessing	High-level control condition	3.29
	Miller et al, 2005	Guessing	Low-level control condition	3.73
	Paulus et al, 2001	Guessing	Low-level control condition	6.97
	Volz et al, 2003	Guessing	High-level control condition	7.12
	Volz et al, 2004	Probabilistic learning under ambiguity	High-level control condition	3.95
	Volz et al, 2005	Probabilistic learning under ambiguity	High-level control condition	11.21
**Thalamus (R)**	**11 out of 27 experiments contributed:**			
	Behrens et al, 2007	Probabilistic learning under ambiguity	Low-level control condition (baseline)	13.09
	Causse et al, 2013	Spatial navigation under ambiguity	Low-level control condition (baseline)	10.11
	D'Cruz et al, 2011	Probabilistic learning under ambiguity	High-level control condition	11.34
	Elliott et al, 1999	Guessing	Low-level control condition	5.41
	Farrar et al, 2018	Guessing	High-level control condition	12.73
	Mestres-Misse et al, 2017	Probabilistic learning under ambiguity	High-level control condition	6.37
	Paulus et al, 2001	Guessing	Low-level control condition	5.26

	Volz et al, 2003	Guessing	High-level control condition	10.85
	Volz et al, 2004	Probabilistic learning under ambiguity	High-level control condition	6.15
	Wiehler et al, 2021	Probabilistic learning under ambiguity	Low-level control condition (baseline)	17.71
	Yoshida & Ishii, 2006	Spatial navigation under ambiguity	Low-level control condition	0.97
**Insular Cortex (L)**	**8 out of 27 experiments contributed:**			
	Behrens et al, 2007	Probabilistic learning under ambiguity	Low-level control condition (baseline)	22.54
	Causse et al, 2013	Spatial navigation under ambiguity	Low-level control condition (baseline)	13.25
	D'Cruz et al, 2011	Probabilistic learning under ambiguity	High-level control condition	15.32
	Gloy et al, 2020	Guessing	Low-level control condition (baseline)	0.68
	Jung et al, 2014	Guessing	High-level control condition	4.81
	Krug et al, 2014	Guessing	Low-level control condition	21.88
	Volz et al, 2004	Probabilistic learning under ambiguity	High-level control condition	7.78
	Volz et al, 2005	Probabilistic learning under ambiguity	High-level control condition	13.64
**Middle Frontal Gyrus (R)**	**8 out of 27 experiments contributed:**			
	Behrens et al, 2007	Probabilistic learning under ambiguity	Low-level control condition (baseline)	3.53
	d'Acremont et al, 2013	Probabilistic learning under ambiguity	Low-level control condition (baseline)	16.89
	D'Cruz et al, 2011	Probabilistic learning under ambiguity	High-level control condition	20.79
	Elliott et al, 1999	Guessing	Low-level control condition	0.71
	Feinstein et al, 2006	Guessing	High-level control condition	9.65
	Gloy et al, 2020	Guessing	Low-level control condition (baseline)	20.88
	Krug et al, 2014	Guessing	Low-level control condition	15.67
	Volz et al, 2004	Probabilistic learning under ambiguity	High-level control condition	11.87
**Inferior Frontal Gyrus (R)**	**8 out of 27 experiments contributed:**			
	Causse et al, 2013	Spatial navigation under ambiguity	Low-level control condition (baseline)	7.76
	d'Acremont et al, 2013	Probabilistic learning under ambiguity	Low-level control condition (baseline)	3.22
	Jung et al, 2014	Guessing	High-level control condition	15.55
	Krug et al, 2014	Guessing	Low-level control condition	13.86

	Miller et al, 2005	Guessing	Low-level control condition	20.57
	Ohira, et al, 2010	Gambling under ambiguity	Low-level control condition	13.36
	Volz et al, 2004	Probabilistic learning under ambiguity	High-level control condition	16.98
	Volz et al, 2005	Probabilistic learning under ambiguity	High-level control condition	8.6
**Superior Parietal Lobule (L)**	**7 experiments out of 27 contributed:**			
	Behrens et al, 2007	Probabilistic learning under ambiguity	Low-level control condition (baseline)	12.92
	Causse et al, 2013	Spatial navigation under ambiguity	Low-level control condition (baseline)	14.43
	Farrar et al, 2018	Guessing	High-level control condition	14.11
	Gloy et al, 2020	Guessing	Low-level control condition (baseline)	14.03
	Jung et al, 2014	Guessing	High-level control condition	20.89
	Volz et al, 2004	Probabilistic learning under ambiguity	High-level control condition	2.76
	Volz et al, 2005	Probabilistic learning under ambiguity	High-level control condition	20.83

Supplementary Table 9. Task contributions in *choice response* category for risk domain

Cluster name	Study	Task type	Average contribution (%)
**Choice response: Risk domain**			
**Paracingulate Gyrus (R/L)**	**13 out of 31 experiments contributed:**		
	Bednarski et al, 2012	Naturalistic risk-taking	4.41
	Burnette et al, 2021	Naturalistic risk-taking	0.83
	Christakou et al, 2009	Naturalistic risk-taking	8.53
	Cohen and Ranganath, 2005	Gambling under risk	7
	Cohen et al, 2005	Gambling under risk	5.76
	Kolling et al, 2014	Gambling under risk	6.38
	Liu et al, 2017	Gambling under risk	0.21
	Pletzer et al, 2016	Naturalistic risk-taking/Gambling under risk	10.67
	Vassena et al, 2014	Gambling under risk	1.3
	Wright et al, 2012	Gambling under risk	17.79
	Wright et al, 2013a	Gambling under risk	7.68
	Xue et al, 2009	Gambling under risk	12.02
	Zhang et al, 2019	Gambling under risk	17.39
**Caudate (R)**	**10 out of 31 experiments contributed:**		
	Campbell-Meiklejohn et al, 2008	Gambling under risk	0.2
	Cohen and Ranganath, 2005	Gambling under risk	15.75
	Cohen et al, 2005	Gambling under risk	9.76
	Ernst et al, 2004	Gambling under risk	15
	Liu et al, 2007	Gambling under risk	13.05
	Pletzer et al, 2016	Naturalistic risk-taking/Gambling under risk	16.22
	Symmonds et al, 2011	Gambling under risk	0.35
	Vorobyev et al, 2015	Spatial navigation under risk	13.83
	Wright et al, 2012	Gambling under risk	1.89
	Zhang et al, 2019	Gambling under risk	13.86
**Caudate (L)**	**9 out of 31 experiments contributed:**		

Campbell-Meiklejohn et al, 2008	Gambling under risk	1.76
Cohen and Ranganath, 2005	Gambling under risk	14.99
Cohen et al, 2005	Gambling under risk	5.65
Ernst et al, 2004	Gambling under risk	13.4
Fukunaga et al, 2018	Gambling under risk	7.58
Pletzer et al, 2016	Naturalistic risk-taking/Gambling under risk	18.1
Symmonds et al, 2011	Gambling under risk	11.33
Vorobyev et al, 2015	Spatial navigation under risk	26.54
Xue et al, 2009	Gambling under risk	0.62

Supplementary Figure 1. Task engagement category across domains (combined experiments with low- and high-level control conditions) and domain contributions

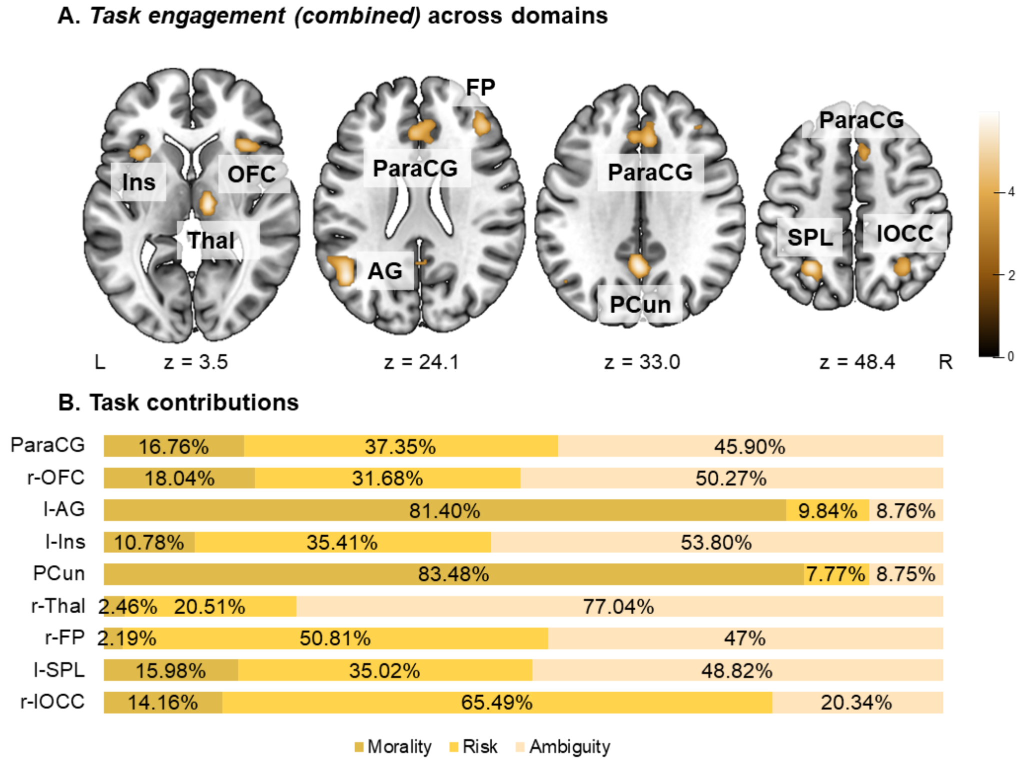

Supplementary Figure 2. Functional decoding results for *task engagement (combined) and choice response* categories

Task engagement (combined)

	ParaCG F-I	ParaCG R-I	r-OFC F-I	r-OFC R-I	l-AG F-I	l-AG R-I	l-Ins F-I	l-Ins R-I	Prec F-I	Prec R-I	r-Thal F-I	r-Thal R-I	r-FP F-I	r-FP R-I	l-SPL F-I	l-SPL R-I	r-lOCC F-I	r-lOCC R-I
Action.Execution												0.09						
Action.Execution.Speech												0.02						
Action.Inhibition			1.59	0.04													2.06	0.01
Action.Imagination																		
Action.Observation																	3.17	0.03
Action.Motor Learning																		
Cognition.Attention																	1.49	0.18
Cognition.Language.Phonology	1.71																	
Cognition.Language.Speech																		
Cognition.Language.Semantics																		
Cognition.Language.Orthography																		
Cognition.Memory.Explicit																		
Cognition.Memory.Working	1.43	0.06											3.6	0.17	1.65	0.07		
Cognition.Reasoning	1.4	0.09	1.46	0.1											1.64	0.11	1.81	0.12
Cognition.Social Cognition					4.77	0.11			5.21	0.12								
Cognition.Spatial																	3.7	0.05
Emotion.Negative.Anxiety			2.09	0.01														
Emotion.Negative.Sadness									3.09									
Emotion.Negative.Punishment/Loss																		
Emotion.Positive.Reward/Gain			1.66	0.09														
Emotion.Valence									5.56	0.04								
Perception.Somesthesis.Pain		0.03	1.75	0.04				0.03			2.61	0.07						
Perception.Olfaction																		
Perception.Gustation																		
Perception.Vision.Colour															2.7	0.01	2.89	0.01
Perception.Vision.Motion																		
Perception.Vision.Shape																	2.45	0.07
Interoception.Sexuality																		
Interoception.Thermoregulation			2.5	0.005				0.005				0.008						

Choice response

	l-CN F-I	l-CN R-I	r-CN F-I	r-CN R-I	l-Ins F-I	l-Ins R-I	ParaCG F-I	ParaCG R-I	r-Ins F-I	r-Ins R-I	l-PreCG F-I	l-PreCG R-I	r-SFG F-I	r-SFG R-I
Action.Execution													1.95	0.11
Action.Execution.Speech														
Action.Inhibition										0.03				
Action.Imagination													3.03	0.02
Action.Observation														
Action.Motor Learning													12.16	0.03
Cognition.Attention	2.42	0.04	1.57	0.16										
Cognition.Language.Phonology					1.89	0.03	2.4							
Cognition.Language.Speech					1.47	0.06					1.56	0.07		
Cognition.Language.Semantics					1.46	0.09								
Cognition.Language.Orthography											2.47	0.04		
Cognition.Memory.Explicit					1.38									
Cognition.Memory.Working											1.86	0.09	2.6	0.11
Cognition.Reasoning	2.66	0.15	2.62	0.15	1.36		1.85	0.13	1.56	0.11				
Cognition.Social Cognition														
Cognition.Spatial													3.97	0.05
Emotion.Negative.Anxiety														
Emotion.Negative.Sadness														
Emotion.Negative.Punishment/Loss			8.94	0.02										
Emotion.Positive.Reward/Gain	4.65	0.23	4.49	0.21	1.39		1.85	0.13	1.74	0.1				
Emotion.Valence														
Perception.Somesthesis.Pain									1.64	0.04				
Perception.Olfaction	3.19													
Perception.Gustation			3.29	0.03										
Perception.Vision.Colour														
Perception.Vision.Motion													3.49	0.06
Perception.Vision.Shape													2.37	0.07
Interoception.Sexuality	2.42	0.04	2.41	0.03										
Interoception.Thermoregulation					2.85	0.007				0.006				

Supplementary Methods and Results:

Methods: Resting-state functional connectivity

Resting-state fMRI images of 192 healthy volunteers (age range 20 – 75 years, mean age 40.36 ± 16.68 years, 67 males) were obtained from the Enhanced Nathan Kline Institute – Rockland Sample (Nooner et al., 2012). The local ethics committee of the Heinrich-Heine University in Düsseldorf approved re-analysis of the data. During RS acquisition, subjects were instructed to look at a fixation cross, not think about anything in particular and not to fall asleep. Images were acquired on a Siemens TimTrio 3T scanner using BOLD contrast [gradient-echo EPI pulse sequence, TR = 1.4 s, TE = 30 ms, flip angle = 65°, voxel size = 2.0 mm × 2.0 mm × 2.0 mm, 64 slices].

Physiological and movement artifacts were removed from the RS data by using FIX (FMRIB's ICA-based Xnoiseifier, version 1.061 as implemented in FSL 5.0.9; (Griffanti et al., 2014; Salimi-Khorshidi et al., 2014), which decomposes the data into independent components (ICs) and identifies noise components using a large number of distinct spatial and temporal features via pattern classification. Unique variance related to the identified artefactual ICs is then regressed from the data together with 24 movement parameters (including derivatives and 2nd order effects as previously described and evaluated; cf. Satterthwaite et al. (2013)).

Data were further preprocessed using SPM12 (Wellcome Trust Centre for Neuroimaging, London) and in-house Matlab scripts. The first four scans were excluded prior to further analyses, the remaining EPI images corrected for head movement using a two-pass (alignment to the initial volume followed by alignment to the mean after the first pass) affine registration. The mean EPI image for each subject was then spatially normalized to the ICBM-152 reference space using the “unified segmentation” approach (Ashburner & Friston, 2005). The resulting deformation was applied to the individual EPI volumes, which were subsequently smoothed with a 5-mm FWHM Gaussian kernel to improve the signal-to-noise ratio and to compensate for residual anatomic variations.

To prepare for functional connectivity analysis from regions-of-interest to the rest of the brain, we have converted the statistical maps of our meta-analytical results in the *task engagement* and *choice response* categories separately for each domain into separate masks for each resulting cluster of convergent activation. Then, the time-course across all voxels in all clusters resulting from the meta-analyses separately for each domain was extracted per subject by computing the first eigenvariate of the time-series of all voxels within the network of interest. To reduce spurious correlations, variance explained by the mean white matter and cerebral spinal fluid signal were removed from the time series, which was subsequently band-pass filtered preserving frequencies between 0.01 and 0.08 Hz. The processed time-course of each seed was then correlated with the (identically processed) time-series of all other grey-matter voxels in the brain using linear (Pearson's) correlation. The resulting correlation coefficients were transformed into Fisher's z-scores, which were entered in a second-level ANOVA for group analysis including age and gender as covariates of no interest. Results were thresholded at $p < 0.05$ corrected for multiple comparisons on the cluster level. The results for each domain were then entered into a conjunction analysis to investigate overlaps of connectivity profiles between domains.

Results: Resting-state functional connectivity of moral, risky, and ambiguous networks in *task engagement*

In this meta-analysis, we aimed to investigate whether a domain-general decision-making circuit underlies different value-based decision-making domains, namely, morality, risk, and ambiguity. As we only discovered three clusters of convergent activity in *task engagement (low-level control condition only)* and no overlapping convergent activation in *task engagement (combined)* between the morality and risk as well as the morality and ambiguity domains in the conjunction analyses, we cannot conclude that a shared domain-general circuit underlies value-based decision-making processing across various domains. However, our results also might have been impacted by differences in the content of experimental tasks, differences in content of the control task and the extent of low-level control conditions

(e.g. resting-state scan vs. motorvisual guided repeat of the task), selected significance thresholds in the original studies and other methodological choices in the original studies, included in this meta-analysis. To test indirectly if a domain-general neural decision-making circuit exists, we additionally performed a resting-state functional connectivity analysis. We created ROI masks from all clusters found in the morality, risk, and ambiguity domains in the *task engagement* category, and performed a functional connectivity analysis from these clusters to the rest of the brain, using the NKI-Rockland sample as an independent yet representative model of adult brain functional connectivity. For each domain we have summed up the connectivity results from each cluster and then performed a three-way conjunction of these results from the three domains. Our results revealed that the domain-specific clusters involved in the three decision-making domains (moral, risky, and ambiguous), are all functionally connected to a large network of brain areas, generally involved in decision-making (executive, language functions). The networks of all three domains are connected to bilateral OFC, IFG, MFG, SMG, AG, lOCC, Cerebellum Crus II and Crus I, Frontal Pole, Hippocampus, Amygdala, CN, NAcc, ACG, PCG, and Precuneus (see Figure 4). For details on the size of the clusters and centre of gravity coordinates as well as connectivity maps for each domain separately.

Supplementary Table 6. Resting state functional connectivity, conjunction of moral, risky, and ambiguous domains in task effect category

Cluster no.	Hemisphere	Brain area	Size (voxels)	x	y	z
1	L	Orbitofrontal cortex, Frontal Operculum Cortex, Inferior Frontal Gyrus, Middle Frontal Gyrus, Precentral Gyrus	3251	-22	10	-24
2	R	Orbitofrontal cortex, Frontal Pole, Inferior Frontal Gyrus, Middle Frontal Gyrus, Superior Frontal Gyrus, Amygdala	2855	24	14	-24
3	R	Middle Temporal Gyrus, Supramarginal Gyrus, Angular Gyrus, Lateral Occipital Cortex	1550	62	-30	-26
4	L	Supramarginal Gyrus, Angular Gyrus, Lateral Occipital Cortex	555	-58	-46	30
5	L	Cerebellum Crus II, Crus I	505	-22	-82	-48
6	R	Caudate, Accumbens	437	10	4	14
7	L	Caudate, Accumbens	282	-10	8	-8
8	R	Cerebellum Crus II, Crus I	254	44	-64	-54

9	R	Frontal Pole	235	14	56	-18
10	L	Cerebellum IX	171	-4	-60	-54
11	L	Inferior Temporal Gyrus, Middle Temporal Gyrus, temporooccipital part	169	-60	-44	-24
12	R	Posterior Cingulate Gyrus	137	2	-38	22
13	R	Cerebellum Crus I	126	10	-78	-40
14	R/L	Brain stem	116	2	-24	-36
15	R	Precuneus	96	22	-62	24
16	L	Hippocampus, Parahippocampal Gyrus, Amygdala	88	-18	-34	-10
17	R/L	Thalamus	62	-4	-8	-4
18	L	Posterior Cingulate Gyrus	54	-4	-38	22
19	L	Superior Temporal Gyrus	26	-68	-40	10
20	R	Anterior Cingulate Gyrus	22	6	30	14
21	R	Subcallosal Cortex	17	8	12	26
22	L	Precuneus	17	-16	-66	26
23	L	Precuneus	17	-8	-72	36
24	L	Planum Temporale	16	-54	-28	4
25	R	Hippocampus	11	20	-38	-2

Note: Cluster size threshold applied at 10 voxels.

Supplementary Figure 3: Conjunction of resting-state functional connectivity results from morality, risk, and ambiguity domains in *task engagement.* Yellow clusters depict shared connectivity targets by convergent activation clusters in morality, risk, and ambiguity domains in task engagement category.

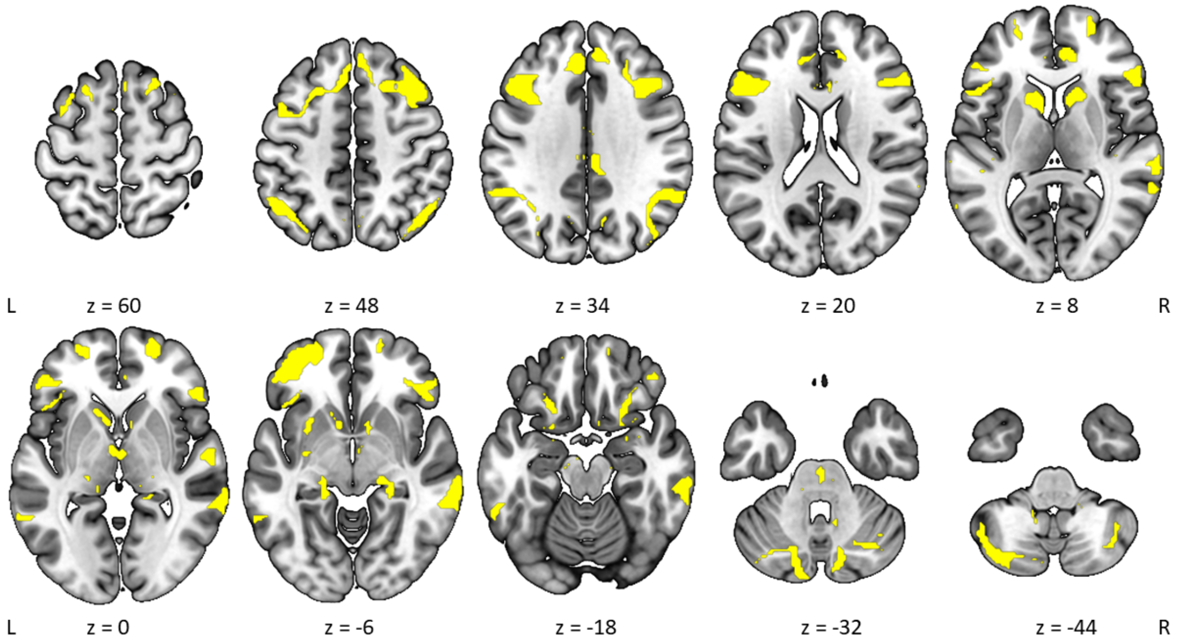

Supplementary Figure 4. Resting-state functional connectivity results for combined nodes in morality domain for *task engagement*

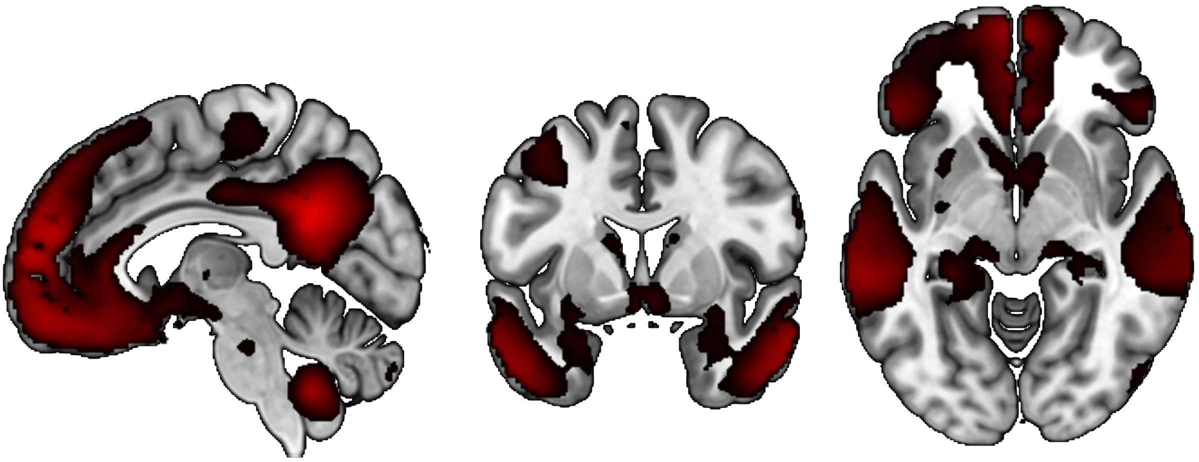

Supplementary Figure 5. Resting-state functional connectivity results for combined nodes in risk domain for *task engagement*

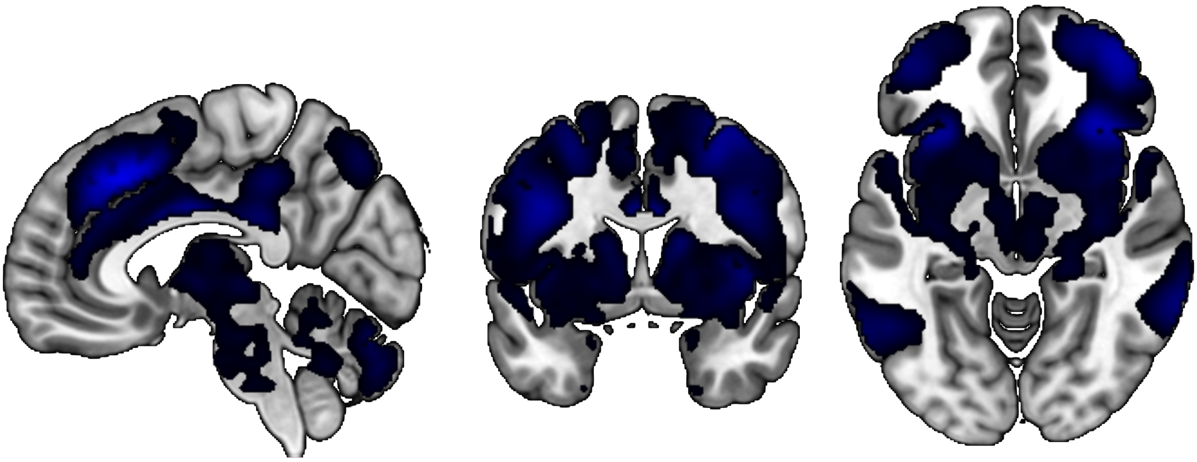

Supplementary Figure 6. Resting-state functional connectivity results for combined nodes in ambiguity domain for *task engagement*

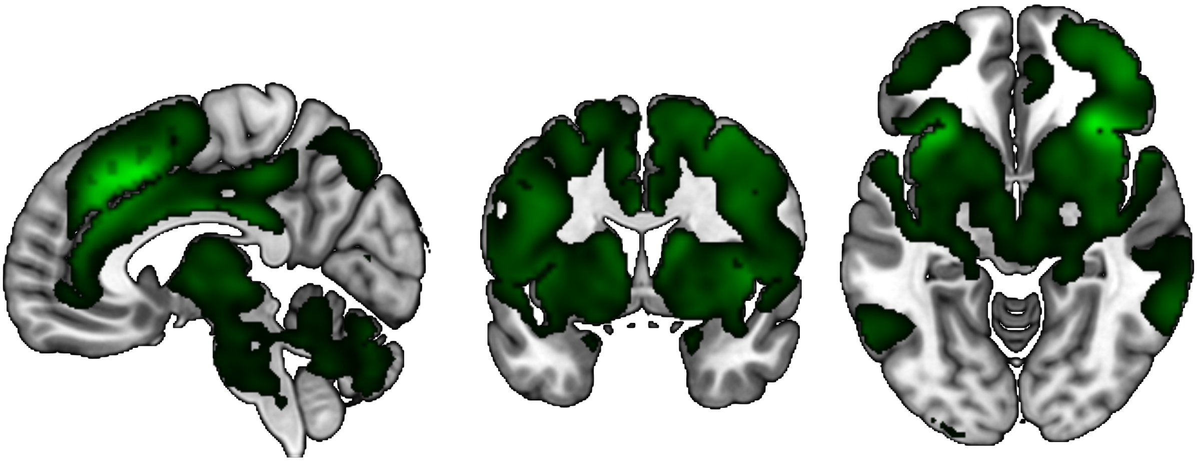

Supplementary Figure 6. Comparison of our results with other recent meta-analyses

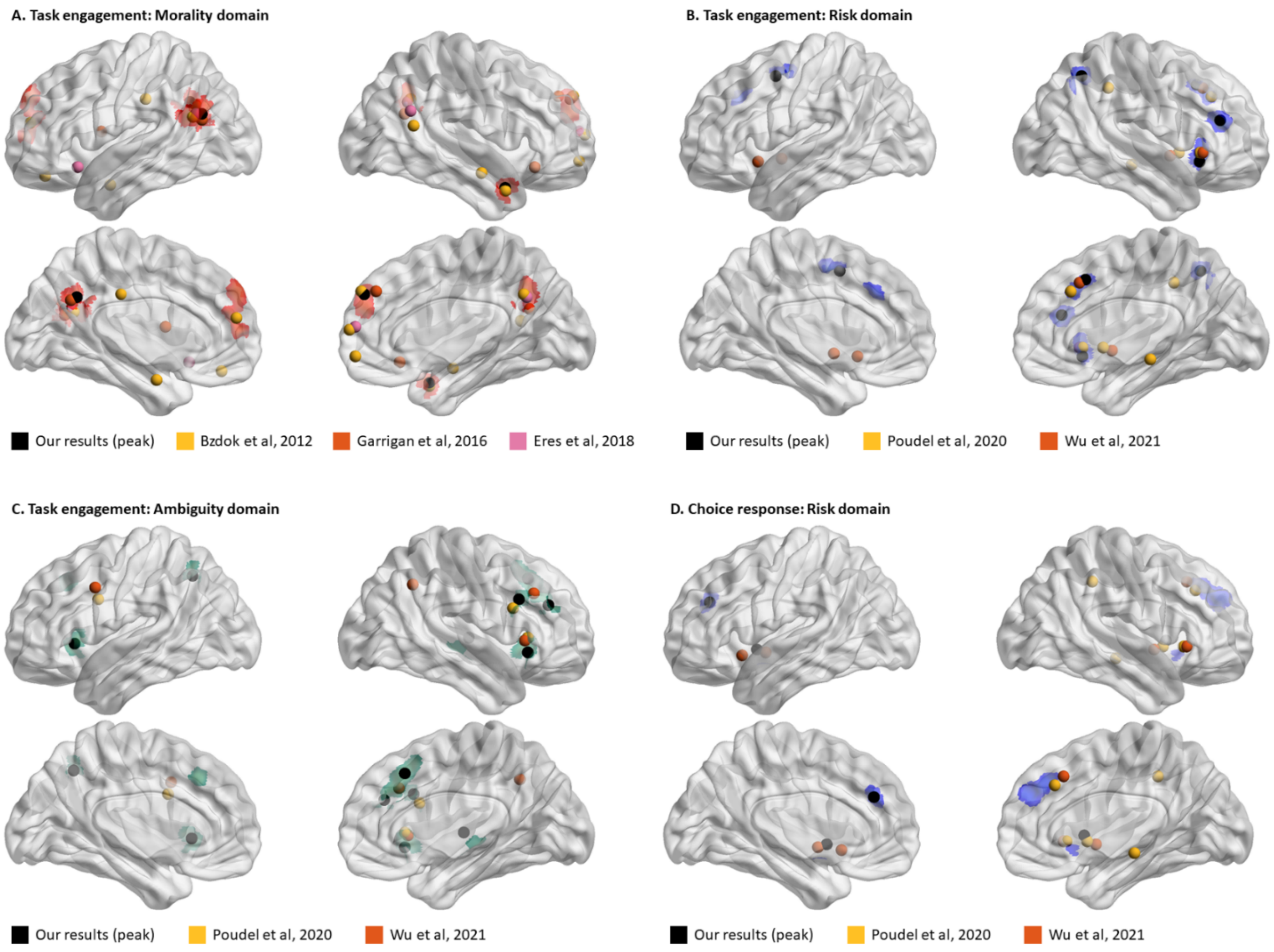

Supplementary Figure 4. *Comparison of results of this meta-analysis and previous meta-analyses in the fields of moral cognition, risk, and ambiguity processing. Our results are depicted as convergence of activation peaks (in black) as well as surface overlays. Overlays of subcortical clusters are not visible and represented by spheres only. Peak coordinates of resulting convergence of activity clusters in other meta-analyses are depicted in spheres of corresponding colour: in morality domain for task engagement, peak convergence coordinates from Bzdok et al. (2012) are depicted in yellow, Garrigan et al. (2016) in red, and Eres et al. (2018) in pink. In risk domain for task engagement and choice response, as well as ambiguity domain for task engagement, peak convergence coordinates from Poudel et al. (2020) are depicted in yellow, and Wu et al. (2021) are depicted in red. Please note that other meta-analyses did not differentiate between task engagement and choice response categories, therefore, in risk domain for task engagement (B) and choice response (D) their peak convergence coordinates are the same, while our correspond to the different results we obtained from task engagement and choice response meta-analyses in the risk domain.*

References

Ashburner, J., & Friston, K. J. (2005). Unified segmentation. *Neuroimage*, *26*(3), 839-851. https://doi.org/10.1016/j.neuroimage.2005.02.018

Bedini, M., & Baldauf, D. (2021). Structure, function and connectivity fingerprints of the frontal eye field versus the inferior frontal junction: A comprehensive comparison. *Eur J Neurosci*, *54*(4), 5462-5506. https://doi.org/10.1111/ejn.15393

Bzdok, D., Schilbach, L., Vogeley, K., Schneider, K., Laird, A. R., Langner, R., & Eickhoff, S. B. (2012). Parsing the neural correlates of moral cognition: ALE meta-analysis on morality, theory of mind, and empathy. *Brain Struct Funct*, *217*(4), 783-796. https://doi.org/10.1007/s00429-012-0380-y

Derrfuss, J., Brass, M., Neumann, J., & von Cramon, D. Y. (2005). Involvement of the inferior frontal junction in cognitive control: meta-analyses of switching and Stroop studies. *Hum Brain Mapp*, *25*(1), 22-34. https://doi.org/10.1002/hbm.20127

Derrfuss, J., Brass, M., von Cramon, D. Y., Lohmann, G., & Amunts, K. (2009). Neural activations at the junction of the inferior frontal sulcus and the inferior precentral sulcus: Interindividual variability, reliability, and association with sulcal morphology. *Hum Brain Mapp*, *30*(1), 299-311. https://doi.org/10.1002/hbm.20501

Eres, R., Louis, W. R., & Molenberghs, P. (2018). Common and distinct neural networks involved in fMRI studies investigating morality: an ALE meta-analysis. *Soc Neurosci*, *13*(4), 384-398. https://doi.org/10.1080/17470919.2017.1357657

Fang, Z., Jung, W. H., Korczykowski, M., Luo, L. J., Prehn, K., Xu, S. H., Detre, J. A., Kable, J. W., Robertson, D. C., & Rao, H. Y. (2017). Post-conventional moral reasoning is associated with increased ventral striatal activity at rest and during task. *Sci Rep-Uk*, *7*. https://doi.org/10.1038/s41598-017-07115-w

Garrigan, B., Adlam, A. L. R., & Langdon, P. E. (2016). The neural correlates of moral decision-making: A systematic review and meta-analysis of moral evaluations and response decision judgements. *Brain Cogn*, *108*, 88-97. https://doi.org/10.1016/j.bandc.2016.07.007

Griffanti, L., Salimi-Khorshidi, G., Beckmann, C. F., Auerbach, E. J., Douaud, G., Sexton, C. E., Zsoldos, E., Ebmeier, K. P., Filippini, N., Mackay, C. E., Moeller, S., Xu, J., Yacoub, E., Baselli, G., Ugurbil, K., Miller, K. L., & Smith, S. M. (2014). ICA-based artefact removal and accelerated fMRI acquisition for improved resting state network imaging. *Neuroimage*, *95*, 232-247. https://doi.org/10.1016/j.neuroimage.2014.03.034

Hartwigsen, G., Neef, N. E., Camilleri, J. A., Margulies, D. S., & Eickhoff, S. B. (2018). Functional Segregation of the Right Inferior Frontal Gyrus: Evidence From Coactivation-Based Parcellation. *Cereb Cortex*, *29*(4), 1532-1546. https://doi.org/10.1093/cercor/bhy049

Iyer, E. S., Weinberg, A., & Bagot, R. C. (2021). Ambiguity and conflict: Dissecting uncertainty in decision-making. *Behav Neurosci*. https://doi.org/10.1037/bne0000489

Lauriola, M., Levin, I. P., & Hart, S. S. (2007). Common and distinct factors in decision making under ambiguity and risk: A psychometric study of individual differences. *Organizational Behavior and Human Decision Processes*, *104*(2), 130-149. https://doi.org/https://doi.org/10.1016/j.obhdp.2007.04.001

McNair, S., Okan, Y., Hadjichristidis, C., & de Bruin, W. B. (2019). Age differences in moral judgment: Older adults are more deontological than younger adults. *J Behav Decis Making*, *32*(1), 47-60. https://doi.org/10.1002/bdm.2086

Müller, V. I., Cieslik, E. C., Laird, A. R., Fox, P. T., Radua, J., Mataix-Cols, D., Tench, C. R., Yarkoni, T., Nichols, T. E., Turkeltaub, P. E., Wager, T. D., & Eickhoff, S. B. (2018). Ten simple rules for neuroimaging meta-analysis. *Neurosci Biobehav Rev*, *84*, 151-161. https://doi.org/10.1016/j.neubiorev.2017.11.012

Nooner, K. B., Colcombe, S. J., Tobe, R. H., Mennes, M., Benedict, M. M., Moreno, A. L., Panek, L. J., Brown, S., Zavitz, S. T., Li, Q., Sikka, S., Gutman, D., Bangaru, S., Schlachter, R. T., Kamiel, S.

M., Anwar, A. R., Hinz, C. M., Kaplan, M. S., Rachlin, A. B., . . . Milham, M. P. (2012). The NKI-Rockland Sample: A Model for Accelerating the Pace of Discovery Science in Psychiatry. *Front Neurosci*, *6*, 152. https://doi.org/10.3389/fnins.2012.00152

Pleskac, T. J. (2008). Decision making and learning while taking sequential risks. *J Exp Psychol Learn Mem Cogn*, *34*(1), 167-185. https://doi.org/10.1037/0278-7393.34.1.167

Poudel, R., Riedel, M. C., Salo, T., Flannery, J. S., Hill-Bowen, L. D., Eickhoff, S. B., Laird, A. R., & Sutherland, M. T. (2020). Common and distinct brain activity associated with risky and ambiguous decision-making. *Drug Alcohol Depend*, *209*, 107884. https://doi.org/10.1016/j.drugalcdep.2020.107884

Pushkarskaya, H., Liu, X., Smithson, M., & Joseph, J. E. (2010). Beyond risk and ambiguity: Deciding under ignorance. *Cogn Affect Behav Ne*, *10*(3), 382-391. https://doi.org/10.3758/Cabn.10.3.382

Ruland, S. H., Palomero-Gallagher, N., Hoffstaedter, F., Eickhoff, S. B., Mohlberg, H., & Amunts, K. (2022). The inferior frontal sulcus: Cortical segregation, molecular architecture and function. *Cortex*, *153*, 235-256. https://doi.org/10.1016/j.cortex.2022.03.019

Salimi-Khorshidi, G., Douaud, G., Beckmann, C. F., Glasser, M. F., Griffanti, L., & Smith, S. M. (2014). Automatic denoising of functional MRI data: combining independent component analysis and hierarchical fusion of classifiers. *Neuroimage*, *90*, 449-468. https://doi.org/10.1016/j.neuroimage.2013.11.046

Satterthwaite, T. D., Elliott, M. A., Gerraty, R. T., Ruparel, K., Loughead, J., Calkins, M. E., Eickhoff, S. B., Hakonarson, H., Gur, R. C., Gur, R. E., & Wolf, D. H. (2013). An improved framework for confound regression and filtering for control of motion artifact in the preprocessing of resting-state functional connectivity data. *Neuroimage*, *64*, 240-256. https://doi.org/10.1016/j.neuroimage.2012.08.052

Schonberg, T., Fox, C. R., & Poldrack, R. A. (2011). Mind the gap: bridging economic and naturalistic risk-taking with cognitive neuroscience. *Trends Cogn Sci*, *15*(1), 11-19. https://doi.org/10.1016/j.tics.2010.10.002

Sebastian, A., Pohl, M. F., Klöppel, S., Feige, B., Lange, T., Stahl, C., Voss, A., Klauer, K. C., Lieb, K., & Tüscher, O. (2013). Disentangling common and specific neural subprocesses of response inhibition. *Neuroimage*, *64*, 601-615. https://doi.org/10.1016/j.neuroimage.2012.09.020

Wu, S., Sun, S., Camilleri, J. A., Eickhoff, S. B., & Yu, R. (2021). Better the devil you know than the devil you don't: Neural processing of risk and ambiguity. *Neuroimage*, *236*, 118109. https://doi.org/10.1016/j.neuroimage.2021.118109
